# Supplementary material for: Scope, context and quality of telerehabilitation guidelines for physical disabilities: a scoping review
Source: BMJ Open. 2021 Aug 12;11(8):e049603. doi: 10.1136/bmjopen-2021-049603 (PMC8361705; doi:10.1136/bmjopen-2021-049603)
Supplement: Supplementary data [file bmjopen-2021-049603supp002.pdf]

Search Strategy Terms

The below search strategy uses a logic grid and is based on the PCC framework; additional columns were added to aid in search sensitivity. As advised by an expert librarian, "concept 3" column was only used to narrow searches that produced an overwhelming quantity of results.

Population 1	*(Population 2)*	*Concept 1*	*Concept 2*	*Concept 3*	*Context*
Physical condition	Covid*	Tele*	Training	Movement	Healthcare
Physical disability	Coronavirus	Virtual	Guidance	Mobility	Health care
Physical impairment	2019-nCoV	Online	Assessment	Function	Clinic
	SARS-CoV-2	Video	Tool*	Gait	Social care
	Severe acute respiratory syndrome coronavirus 2	Digital	Protocol	Walking	Hospital
		Multimedia	Battery	Balance	Practice
		Mobile		Motor	
		Phone		Strength	
		mHealth		Power	
		eHealth		Tone	
				Range	
				Contracture	
				Fatigue	
				Stamina	
				Dexterity	
				Physical capacity	

Search term strategy:

1. Each column was searched individually, e.g. physical condition OR physical disability OR physical impairment
2. All individual searches based on each column were combined, e.g. (physical condition OR physical disability OR physical impairment)AND (Tele* OR Virtual OR Online OR Video OR Digital OR Multimedia OR Mobile OR Phone OR mHealth OR eHealth)
3. The search strategy proceeded as follows (after searching for each column individually):
    a. Column 1 AND column 3 AND column 4 AND column 5
    b. If there were too many hits based on a., column 6 wasto be added

   c. Column 2 was only to be used as an add-on after searches to keep a separate record of covid-related literature for the narrative synthesis
4. Unpublished literature searches used a combination of column 1 and 3 in the first instance, where searches were tailored according to individual database searching tools.
5. Date limiter: 2015 onwards.
