# Supplementary material for: Scope, context and quality of telerehabilitation guidelines for physical disabilities: a scoping review
Source: BMJ Open. 2021 Aug 12;11(8):e049603. doi: 10.1136/bmjopen-2021-049603 (PMC8361705; doi:10.1136/bmjopen-2021-049603)
Supplement: Supplementary data [file bmjopen-2021-049603supp003.pdf]

Record of Online Searches

Database: PubMed
Date: 20/07/20

ID	Search Term(s)	Hits
1	(physical condition) OR (physical disability) OR (physical impairment)	641,602
2	(tele*) OR (virtual) OR (online) OR (video) OR (digital) OR (multimedia) OR (mobile) OR (phone) OR (mhealth) OR (ehealth)	862,859
3	(training) OR (guidance) OR (assessment) OR (tool*) OR (protocol) OR (battery)	5,791,855
4	(movement) OR (mobility) OR (function) OR (gait) OR (walking) OR (balance) OR (motor) OR (strength) OR (power) OR (tone) OR (range) OR (contracture) OR (fatigue) OR (stamina) OR (dexterity) OR (physical capacity)	15,600,817
5	(ID 1) AND (ID 2) AND (ID 3) AND (ID 4) with date limiter: 2015 onwards	5,322

Database: CINHAL
Date: 03/08/20

ID	Search Term(s)	Hits
1	(physical condition) OR (physical disability) OR (physical impairment)	52,385
2	(tele*) OR (virtual) OR (online) OR (video) OR (digital) OR (multimedia) OR (mobile) OR (phone) OR (mhealth) OR (ehealth)	267,357
3	(training) OR (guidance) OR (assessment) OR (tool*) OR (protocol) OR (battery)	1,100,793
4	(movement) OR (mobility) OR (function) OR (gait) OR (walking) OR (balance) OR (motor) OR (strength) OR (power) OR (tone) OR (range) OR (contracture) OR (fatigue) OR (stamina) OR (dexterity) OR (physical capacity)	902,661
5	(ID 1) AND (ID 2) AND (ID 3) AND (ID 4) with date limiter: 2015 onwards	197

Database: PsychInfo
Date: 03/08/20

ID	Search Term(s)	Hits
1	(physical condition) OR (physical disability) OR (physical impairment)	148,089
2	(tele*) OR (virtual) OR (online) OR (video) OR (digital) OR (multimedia) OR (mobile) OR (phone) OR (mhealth) OR (ehealth)	240,422

3	(training) OR (guidance) OR (assessment) OR (tool*) OR (protocol) OR (battery)	1,057,337
4	(movement) OR (mobility) OR (function) OR (gait) OR (walking) OR (balance) OR (motor) OR (strength) OR (power) OR (tone) OR (range) OR (contracture) OR (fatigue) OR (stamina) OR (dexterity) OR (physical capacity)	1,159,877
5	(ID 1) AND (ID 2) AND (ID 3) AND (ID 4) with date limiter: 2015 onwards	557

Database: Cochrane Library (Reviews Only)
Date: 03/08/20

ID	Search Term(s)	Hits
1	(physical condition OR physical disability OR physical impairment) AND<br><br>(tele* OR virtual OR online OR video OR digital OR multimedia OR mobile OR phone OR mhealth OR ehealth) AND<br><br>(training OR guidance OR assessment OR tool* OR protocol OR battery) AND<br><br>(movement OR mobility OR function OR gait OR walking OR balance OR motor OR strength OR power OR tone OR range OR contracture OR fatigue OR stamina OR dexterity OR physical capacity)<br><br>with date limiter: 2015 onwards	17

Database: EMBASE
Date: 05/08/20

ID	Search Term(s)	Hits
1	physical condition OR physical disability OR physical impairment	31,388
2	tele* OR virtual OR online OR video OR digital OR multimedia OR mobile OR phone OR mhealth OR ehealth	973,017
3	training OR guidance OR assessment OR tool* OR protocol OR battery	457,1408
4	movement OR mobility OR function OR gait OR walking OR balance OR motor OR strength OR power OR tone OR range OR contracture OR fatigue OR stamina OR dexterity OR physical capacity	700,1843
5	(ID 1) AND (ID 2) AND (ID 3) AND (ID 4) with date limiter: 2015 onwards	157

Database: Web of Science
Date: 05/08/20

ID	Search Term(s)	Hits
1	TS=(physical condition OR physical disability OR physical impairment)	277,584
2	TS=(tele* OR virtual OR online OR video OR digital OR multimedia OR mobile OR phone OR mhealth OR ehealth)	2,360,472
3	TS=(training OR guidance OR assessment OR tool* OR protocol OR battery)	4,974,907
4	TS=(movement OR mobility OR function OR gait OR walking OR balance OR motor OR strength OR power OR tone OR range OR contracture OR fatigue OR stamina OR dexterity OR physical capacity)	12,757,447
5	(ID 1) AND (ID 2) AND (ID 3) AND (ID 4) with date limiter: 2015 onwards	1,720

Database: PEDro
Date: 06/08/20

ID	Search Term(s)	Hits
1	Tele* AND training with date limiter: 2015 onwards	79
2	Tele* AND guidance with date limiter: 2015 onwards	12
3	mHealth AND training with date limiter: 2015 onwards	8
4	mHealth AND guidance with date limiter: 2015 onwards	1
5	eHealth AND training with date limiter: 2015 onwards	1
6	eHealth AND guidance with date limiter: 2015 onwards	0

Database: UK Health Forum
Date: 11/08/20

ID	Search Term(s)	Hits
1	Tele	0
2	Telehealth	0
3	Telemedicine	0
4	Telerehabilitation	0
5	Tele rehabilitation	0
6	mhealth	0
7	ehealth	1

8	Virtual	0
9	Online	4
10	Video	0
11	Digital	0
12	Multimedia	0
13	Mobile	0
14	Phone	0

Database: National Archives (UK)
Date: 06/08/20

ID	Search Term(s)	Hits
1	Tele* with date limiter: 2015 onwards	37
2	mhealth with date limiter: 2015 onwards	0
3	ehealth with date limiter: 2015 onwards	1

Within each search of this database, hits represent websites that can be searched using National Archives. Each database was searched using “training” or “guidance”; no hits were returned in any searches.

Database: WHO
Date: 11/08/20

ID	Search Term(s)	Hits
1	Telehealth AND training with date limiter: 2015 onwards	118
2	Telemedicine AND training with date limiter: 2015 onwards	353
3	Telemedicine AND guidance with date limiter: 2015 onwards	294
4	Telehealth AND guidance with date limiter: 2015 onwards	111
5	mHealth AND training with date limiter: 2015 onwards	384
6	mHealth AND guidance with date limiter: 2015 onwards	344

Database: NHS England
Date: 11/08/20

ID	Search Term(s)	Hits
1	Telehealth with date limiter: 2015 onwards	19

2	Telemedicine with date limiter: 2015 onwards	19
3	ehealth with date limiter: 2015 onwards	18
4	mhealth with date limiter: 2015 onwards	22

Database: Charted Society of Physiotherapy
Date: 19/08/20

**ID**	**Search Term(s)**	**Hits**
1	Tele*	12
2	mHealth	4
3	eHealth	23
4	Tele* AND training	3538
5	Tele* AND guidance	2849
