# Supplementary material for: Scope, context and quality of telerehabilitation guidelines for physical disabilities: a scoping review
Source: BMJ Open. 2021 Aug 12;11(8):e049603. doi: 10.1136/bmjopen-2021-049603 (PMC8361705; doi:10.1136/bmjopen-2021-049603)
Supplement: Supplementary data [file bmjopen-2021-049603supp004.pdf]

Procedural Guidance for Care Delivery – Derived from Recommendations Analysis

While the information below has been derived from articles that explored movement impairment, none of the recommended actions were specific for movement impairment interventions.

Procedural Guidance	Description
Pre-Delivery<br>▪ Environment Considerations<br>▪ Session Planning<br>▪ Technology Considerations<br>▪ Preparations with Client	In preparation for a telerehabilitation session, including actions such as planning the session environment, preparing the required technology, contacting the client, etc.
During Delivery<br>▪ Start<br>▪ Throughout<br>▪ End	During a telerehabilitation session, including actions such as introducing the agenda for that session, noting technical and safety concerns, summarising the session, etc.
Post-Delivery	After a telerehabilitation session, including actions such as documenting key messages of the session, deciding whether the patient needs further/additional support, and taking breaks when needed.

Pre-Delivery

Thirteen articles[1-13] suggested actions to take before engaging in a telerehabilitation session. These actions can be divided into Preparations with the Client, Environment Considerations, Session Planning, and Technology Considerations.

- Preparations with the Client: Contact the client to assess whether they are clinically appropriate to engage in telerehabilitation (e.g. whether face-to-face or urgent care is needed instead). If telerehabilitation is deemed appropriate, ensure the client has all necessary equipment (including suitable data/WIFI accessibility) and, if required, test any equipment with the client. Provide any information the client needs to attend the telerehabilitation session (e.g. link to logon to session, passwords, times, private and well-lit room, or other resources.). Ensure the client's phone number is available in case of technological issues during the session. Consider the client's context (e.g. hard of hearing or anxious about using technology) and address the context as appropriate if possible.
- Environment Considerations: Ensure the location from which one provides the telerehabilitation is quiet, private, not cluttered and will not be disturbed. Curtains should be drawn to reduce any on-screen glare from the sun. Clients should ensure the location is well-lit without relying on the sun. Test any technology (especially video and audio) to check they are working properly. If conducting a group session, ensure the size of the class is appropriate for the activities planned.
- Session Planning: Evaluate the client's functional status prior to the session and develop a clear agenda. Consider whether a client would benefit from a group or one-to-one session. If planning a group session, ensure there is enough time to address everyone's technological issues (e.g. connectivity issues, audio issues, etc.). Consider how many other staff members you may need (e.g. an extra member for support during a group session).

- Technology Considerations: Ensure any technology used is appropriate for the agenda of that session. Ensure there are back-up devices that can be used (e.g. telephone) if the main technology fails. Professional and patient clients should only open the software needed for the session and all other software should be closed. Ensure internet-using technology is wired to or close to the WIFI router, and that all required equipment are close-by. Position any cameras so that the whole face is within direct view. Ensure that all providers know how to use the required technology (e.g. knowing how to record or take screen shots) prior to the session.

During Delivery
Seven articles[1, 5, 7-11] suggested actions that should occur during the telerehabilitation session, which can be divided into the Start of the session, During the session, and the End of the session.

- Start: Welcome the client and ensure audio and video are working correctly. Confirm the identity of everyone attending the session. If a trusted other (e.g. carer) is also attending, check that the client is comfortable with that trusted other. If possible, lock the meeting to prevent others from entering. During a group session, ensure everyone in the group introduces themselves (including all staff). Summarise the session's agenda and introduce the roles of any trusted other or staff (e.g. assisting with note taking). Ensure the client(s) know what to do if the session is cut. Ask the client if they have any questions or concerns before engaging in the telerehabilitation session. Ensure the client is still clinically appropriate to engage in telerehabilitation (i.e. they do not need face-to-face or urgent care). If taking screen shots or recording, ensure the client knows and you have their consent. Finally, ask the client to show their environment to ensure any rehabilitation activities can be conducted safely and accurately.
- During: Document activities as per your professional governing body requirements, yet also include any technical issues and safety concerns from the client. Be patient during technical glitches (e.g. blurred video or audio cutting out). Understand that communication is harder for the client and ensure communication is intentional (e.g. verbal cues to let the client know you are listening). Ensure that you encourage the client to engage (e.g. by asking questions). The professional should inform the client when and why they might be pre-occupied (e.g. taking notes or looking for a resource).
- End: Carefully and clearly summarise key messages from the session. Let the client know if another session is required and when that will take place. Similarly, let the client know the required details if they need to be referred to another service. Confirm and record that the client is comfortable in engaging in further telerehabilitation sessions. Ensure the client knows self-care activities and their regime. Ask the client if anything needs to be clarified. Provide the client contact details if they need to contact the provider again. When ending the call, let the client know explicitly and then end the call.

Post Delivery
Three articles[5, 10, 11] suggested actions that should occur after a telerehabilitation session. Re-evaluate the client's functional ability. Document key messages of the session as required. Consider if a follow-up call or other structural support is needed for the client. Ensure to take breaks between telerehabilitation sessions to reduce any fatigue and look after vocals.

References

1. AHPScot, *A guide to Musculoskeletal (MSK) Digital Health consultations during Coronavirus (COVID-19)*. 2020.
2. Anton, D., et al., *A Telerehabilitation System for the Selection, Evaluation and Remote Management of Therapies.* Sensors (Basel). **18**(5).
3. Block, V.A., et al., *Remote Physical Activity Monitoring in Neurological Disease: A Systematic Review.* PLoS One, 2016. **11**(4): p. e0154335.
4. Calvaresi, D., et al., *Real-time multi-agent systems for telerehabilitation scenarios.* Artif Intell Med. **96**: p. 217-231.
5. Health and Social Care Board, *AHP Virtual Consultation Guidance*. 2020.
6. Paneroni, M., et al., *Is Telerehabilitation a Safe and Viable Option for Patients with COPD? A Feasibility Study.* Copd. **12**(2): p. 217-25.
7. Charted Society of Physiotherapy, *COVID-19: guide for rapid implementation of remote consultations*. 2020.
8. Royal College of Occupational Therapists, *Virtual Assessments/Reviews: The Script.* 2020.
9. Royal College of Speech and Language Therapists, *MAINTAINING SECURITY FOR TELEHEALTH SESSIONS*. 2020.
10. East Lancashire Hospitals NHS Trust, *Telepractice dysphagia assessmentsGuidelines for use with a patient in their own home/nursing home*. 2020.
11. Middleton, A., et al., *COVID-19 Pandemic and Beyond: Considerations and Costs of Telehealth Exercise Programs for Older Adults With Functional Impairments Living at Home-Lessons Learned from a Pilot Case Study*. Phys Ther.
12. Charted Society of Physiotherapy, *Telephone guidance for musculoskeletal practice*. 2020.
13. Charted Society of Physiotherapy, *Digital tools to support service delivery*. 2020.
