# Supplementary material for: Scope, context and quality of telerehabilitation guidelines for physical disabilities: a scoping review
Source: BMJ Open. 2021 Aug 12;11(8):e049603. doi: 10.1136/bmjopen-2021-049603 (PMC8361705; doi:10.1136/bmjopen-2021-049603)
Supplement: Author's manuscript [file bmjopen-2021-049603.draft_revisions.pdf]

BMJ Open is committed to open peer review. As part of this commitment we make the peer review history of every article we publish publicly available.

When an article is published we post the peer reviewers' comments and the authors' responses online. We also post the versions of the paper that were used during peer review. These are the versions that the peer review comments apply to.

The versions of the paper that follow are the versions that were submitted during the peer review process. They are not the versions of record or the final published versions. They should not be cited or distributed as the published version of this manuscript.

BMJ Open is an open access journal and the full, final, typeset and author-corrected version of record of the manuscript is available on our site with no access controls, subscription charges or pay-per-view fees (http://bmjopen.bmj.com).

If you have any questions on BMJ Open's open peer review process please email info.bmjopen@bmj.com

BMJ Open

The scope, context, and quality of telerehabilitation guidelines for physical disabilities: a scoping review

Journal:	*BMJ Open*
Manuscript ID	bmjopen-2021-049603
Article Type:	Original research
Date Submitted by the Author:	01-Feb-2021
Complete List of Authors:	Anil, Krithika; University of Plymouth, School of Health Professions<br>Freeman, Jennifer; Peninsula Allied Hlth Ctr, School of Health Professions<br>Buckingham, Sarah; University of Plymouth, School of Health Professions<br>Demain, Sara; Plymouth University, School of Health Professions<br>Gunn, H; University of Plymouth, School of Health Professions<br>Jones, Ray; Plymouth University, School of Nursing and Midwifery<br>Logan, Angela; Royal Devon and Exeter NHS Foundation Trust<br>Marsden, Jonathan; Plymouth University, School of Health Professions<br>Playford , Diane ; University of Warwick, Warwick Medical School<br>Sein, Kim; University of Plymouth, School of Health Professions<br>Kent, Bridie; Plymouth University, Nursing and Midwifery, Innovations in Health and Social Care: A JBI Centre of Excellence
Keywords:	COVID-19, Telemedicine < BIOTECHNOLOGY & BIOINFORMATICS, Physiology < NATURAL SCIENCE DISCIPLINES, Protocols & guidelines < HEALTH SERVICES ADMINISTRATION & MANAGEMENT, REHABILITATION MEDICINE

SCHOLARONE™
Manuscripts

I, the Submitting Author has the right to grant and does grant on behalf of all authors of the Work (as defined in the below author licence), an exclusive licence and/or a non-exclusive licence for contributions from authors who are: i) UK Crown employees; ii) where BMJ has agreed a CC-BY licence shall apply, and/or iii) in accordance with the terms applicable for US Federal Government officers or employees acting as part of their official duties; on a worldwide, perpetual, irrevocable, royalty-free basis to BMJ Publishing Group Ltd ("BMJ") its licensees and where the relevant Journal is co-owned by BMJ to the co-owners of the Journal, to publish the Work in this journal and any other BMJ products and to exploit all rights, as set out in our licence.

The Submitting Author accepts and understands that any supply made under these terms is made by BMJ to the Submitting Author unless you are acting as an employee on behalf of your employer or a postgraduate student of an affiliated institution which is paying any applicable article publishing charge ("APC") for Open Access articles. Where the Submitting Author wishes to make the Work available on an Open Access basis (and intends to pay the relevant APC), the terms of reuse of such Open Access shall be governed by a Creative Commons licence – details of these licences and which Creative Commons licence will apply to this Work are set out in our licence referred to above.

Other than as permitted in any relevant BMJ Author's Self Archiving Policies, I confirm this Work has not been accepted for publication elsewhere, is not being considered for publication elsewhere and does not duplicate material already published. I confirm all authors consent to publication of this Work and authorise the granting of this licence.

For peer review only

Title Page

Manuscript Title: The scope, context, and quality of telerehabilitation guidelines for physical disabilities: a scoping review

Word Count: 3910

Authors: Krithika Anil[1] (0000-0002-8027-1665), Jennifer A Freeman[2], Sarah Buckingham[1], Sara Demain[3], Hilary Gunn[4], Ray B Jones[5], Angela Logan[6], Jonathan Marsden[7], E Diane Playford[8], Kim Sein[1], Bridie Kent[9,10]

[1] Research Fellow; Peninsula Allied Health Centre, School of Health Professions, University of Plymouth, Derriford Road, Plymouth, PL6 8BH, United Kingdom

[2] Professor of Physiotherapy and Rehabilitation, Peninsula Allied Health Centre, School of Health Professions, University of Plymouth, Derriford Road, Plymouth, PL6 8BH, United Kingdom

[3] Head of School of Health Professions, Peninsula Allied Health Centre, School of Health Professions, University of Plymouth, Derriford Road, Plymouth, PL6 8BH, United Kingdom

[4] Associate Professor in Physiotherapy, Peninsula Allied Health Centre, School of Health Professions, University of Plymouth, Derriford Road, Plymouth, PL6 8BH, United Kingdom

[5] Professor of Health Informatics, School of Nursing and Midwifery, University of Plymouth, Drake Circus, Plymouth, PL4 8AA, United Kingdom

[6] Consultant Physiotherapist, Stroke Rehabilitation, Royal Devon and Exeter NHS Foundation Trust, Barrack Road, Exeter, Devon, EX2 5DW, United Kingdom

[7] Professor and Chair in Rehabilitation, Peninsula Allied Health Centre, School of Health Professions, University of Plymouth, Derriford Road, Plymouth, PL6 8BH, United Kingdom

[8] Honorary Professor of Neurological Rehabilitation, Warwick Medical School, Gibbet Hill Campus, University of Warwick, CV4 7AL, United Kingdom

[9] Professor of Leadership in Nursing, School of Nursing and Midwifery, University of Plymouth, Drake Circus, Plymouth PL4 8AA, United Kingdom

[10] Professor of Leadership in Nursing, Director of the University of Plymouth Centre for Innovations in Health and Social Care: A JBI Centre of Excellence, University of Plymouth, Drake Circus, Plymouth PL4 8AA, United Kingdom

Corresponding author: Krithika Anil
Contact email: krithika.anil@plymouth.ac.uk

For peer review only - http://bmjopen.bmj.com/site/about/guidelines.xhtml

ABSTRACT

Objective To identify the available guidance and training to implement telerehabilitation movement assessments for people (adults and children) with a physical disability, including those recovering from COVID-19

Design Rapid scoping review

Included Sources and Articles PubMed, CINAHL, PsychInfo, Cochrane, EMBASE, Web of Science, PEDro, UK Health Forum, WHO, National Archives, and NHS England, searched using the Participant-Concept-Context (PCC) framework from 2015 to August 2020. Primary studies that recruited individuals with physical disabilities and guidance documents aimed at providers to implement movement-related telerehabilitation were included.

Results 23 articles (11 primary research studies, 3 systematic reviews and 9 guidance documents) were included out of 7857 that were identified from the literature search. Two main issues were found: (1) telerehabilitation guidance (from both research studies and guidance documents) was not specific to movement-related assessment, and (1) most primary research studies provided neither guidance nor training of movement-specific assessment to practitioners. Of the COVID-19-related guidance, two articles reported COVID-19 management that only referred to identifying COVID-19 status without references to specific movement-related guidance.

Conclusions Telerehabilitation guidance and training have existed pre-COVID-19, yet the lack of specific movement-related information and provider support is surprising. This gap must be addressed to optimise effective implementation of remote assessments for those with physical disabilities.

Review Registration Open Science Framework: osf.io/vm6sp

Key words: Telerehabilitation, physical disability, remote consultation, COVID-19, scoping review, guidance

STRENGTHS AND LIMITATIONS

- A wide variety of articles were reviewed due to extensive searches for grey literature and no language restrictions
- Despite no language restrictions, included articles were biased towards the English language
- Some articles within this review may have been updated since the initial search and others have likely emerged since

INTRODUCTION

Since the outbreak of the COVID-19 pandemic, health services across the world have rapidly adapted to a new way of working. They have embraced the need to use a "digital first" approach to maintain delivery of care as best as possible, whilst protecting both staff and patients[1]. In the field of rehabilitation this approach is typically referred as "telerehabilitation", defined as: The delivery of rehabilitation services via information and communication technologies[2]. This includes a broad range of services from assessment and monitoring to education and consultation. During COVID-19, the use of telephone and video consultations in particular has escalated and although, compared to the initial United Kingdom lockdown, there has been some return to face-to-face services in more recent months, there is a general acknowledgement that remote delivery of services will be an integral part of everyday practice within future health and social care systems. Ensuring that the delivery of such services is both effective and equitable is pertinent, given the identified "tsunami" of rehabilitation need[3 4] for people with a wide range of conditions from across the life-span, including those recovering from COVID-19[5].

Key to targeting an effective and personalized rehabilitation plan is the need for a comprehensive, detailed, and valid assessment[6], with ongoing monitoring and evaluation as needs change. When the primary concerns relate to physical disability, an assessment of aspects such as dynamic posture, balance and movement is fundamental. This therefore raises important questions related to how

For peer review only - http://bmjopen.bmj.com/site/about/guidelines.xhtml

these assessments can be undertake remotely, whilst ensuring they are both safe and effective. For instance, is it possible to adequately and safely assess the balance of an individual who is falling/at risk of falls or the source of shoulder pain? Or the relative impact of spasticity and weakness on movement? Do the additional practical challenges such as slow internet speeds and static and restricted camera angles mean that an assessment of dynamic movement is too restricted to inform management plans? Are the clinical skills required for this type of remote assessment different to those utilised for face-to-face assessment? And if so, is there specific guidance and training related to these aspects of remotely based physical assessments to assist practitioners with implementing this effectively and efficiently? It was our observation that questions such as these have been raised by rehabilitation organisations, professional networks and individual practitioners and require urgent answers, hence the need for this rapid scoping review.

Review question and objectives

What guidance and training is available to implement telerehabilitation movement assessment for people (children and adults) with a physical disability, including those recovering from COVID-19? The review objectives were to identify:

- The contexts and types of telerehabilitation assessments delivered in a health and social care setting for people with a physical disability (including those recovering from COVID-19)
- Any specific guidance and/or training available for delivering telerehabilitation movement-related assessments
- Specific recommendations regarding movement-related assessments for effective implementation of telerehabilitation guidance

METHODS

This scoping review formed part of an overall research project aimed at developing and evaluating a training package to support the remote assessment and management of people with physical disability (UK RI-NIHR, MRC, Covid-19: MR/V021060/1). It was not appropriate to involve patients or the public in the design, or conduct, or reporting of this research.

Type of Review

This scoping review used the JBI approach[7]. This approach seeks to explore the breadth or extent of the literature, map and summarise the evidence, and inform future research[8]. The PRISMA checklist for scoping reviews was used for the review (see supplementary material titled "SM – PRISMA Statement for Scoping Reviews"). There is no registration database specific for scoping reviews, thus the review was uploaded to Open Science Framework: osf.io/vm6sp.p9

Patient and Public Involvement

No patients involved.

Inclusion Criteria

The inclusion criteria were developed using the PCC (participant-concept-context) framework[9].

Participants

The review considered articles that included individuals of all ages with a physical disability. Physical disability was defined as any physical condition that affects a person's mobility, physical capacity, stamina, or dexterity. The review included studies of participants with a physical disability who had

For peer review only - http://bmjopen.bmj.com/site/about/guidelines.xhtml

any duration and severity of physical disability. There was no age restriction. Guidance and policy documents aimed at undertaking telerehabilitation with individuals with physical disabilities were also included.

Concept

Studies and policies that examined or described guidance and training regarding implementation of telerehabilitation movement assessments were considered. *Guidance* included checklists, tools, outcome measurement batteries, protocols, guidelines, risk assessments, exemplars, and appraisals. *Training* included interactions, communications, and resources with the aim to improve and/or inform the process of delivering telerehabilitation. Guidance and training must have involved movement evaluation; that is, the evaluation of how people move around, perform, and function. This included self-administered and practitioner administered telerehabilitation. Studies and policies that did not involve movement evaluation were excluded.

Context

This review considered studies that provided guidance and training in health and social care contexts, that is, telerehabilitation must have been provided by a practitioner, system, or institute.

Types of Literature

The following study designs were included: randomised controlled trials (RCTs), non-RCTs, quasi-experimental, pre- and post- studies, case studies, observational studies, systematic reviews, and qualitative studies. Grey literature regarding policies from recognised UK-based national and international institutes were also considered. Opinion pieces, non-systematic literature reviews, and other grey literature were excluded. No language restrictions were implemented.

Date Restriction

Publications from 2015 to Aug 2020 were included in this review because technologies and related-guidance older than this period were considered out-of-date due to the rapid speed of technology development in areas such as robotics, artificial intelligence, and telepresence[10 11].

Search Strategy

An initial limited search of PubMed and CINAHL was undertaken to estimate the volume of relevant literature and to identify key words to assist in developing search terms. A second search using the developed search terms was undertaken (supported by a review-specialist librarian) and adapted across each included information source (PubMed, CINAHL, PsychInfo, Cochrane, EMBASE, Web of Science, PEDro, UK Health Forum, WHO, National Archives, NHS England). Due to the rapid nature of this review, study authors were not contacted for further information in cases of ongoing or uncompleted studies. Please see supplementary materials (SM1 and SM2) for search strategy terms.

Study Selection

Following the search, all identified references were imported into Endnote[12] (a reference management software). After removal of duplications within EndNote, references were uploaded to the online Rayyan tool[13]; this tool is designed specifically for the organisation of studies for a systematic review and can also be used to coordinate study selection amongst review team members. Titles and abstracts were screened for assessment against the inclusion criteria for the review. The full text of potentially eligible studies were retrieved and assessed in detail against the inclusion criteria. Full text studies that did not meet the inclusion criteria were excluded and are displayed in a PRISMA flowchart (see Figure 1). Any disagreements that arose between the reviewers were resolved through discussion. As this review is a rapid scoping review no quality or risk of bias assessment was undertaken.

For peer review only - http://bmjopen.bmj.com/site/about/guidelines.xhtml

Data Extraction

Data extraction of included studies was conducted by the review team. Data was charted using a customised Excel spreadsheet. The data extracted specific information pertaining to the review objectives and key information for each article were charted as per JBI Guidance[7]. This included telerehabilitation contexts, types of telerehabilitation, guidance and training, and any recommendations for implementing telerehabilitation. Any disagreements between reviewers were resolved through discussion.

Data Synthesis

The narrative synthesis of the findings from included studies, policies, and guidance documents was structured according to the review objectives. Findings were presented narratively, aided by appropriate tables and figures. Recommendations in the literature were analysed using descriptive qualitative analysis[14] to identify common themes. Only recommendations focusing on clinical implementation or technological improvements were analysed (recommendations for future research within the included studies were not analysed).

Mentions of the 'provider' refer to the individual providing the telerehabilitation, such as health or social care practitioners. Mentions of the 'client' refer to the individual receiving the telerehabilitation, i.e. the patient.

RESULTS

Twenty-three articles were included in the scoping review analysis (Figure 1; see Table 1 for article characteristics). Eleven[15-25] of the included articles were original research studies and three were systematic review studies[26-28]. The remaining nine[29-37] were guidance documents produced by health institutions or health experts. Guidance documents are highlighted in grey and systematic review articles are highlighted in yellow in Table 1. Eleven articles were specified for adults[15-19 21-24 26 27] (≥18 years), one article was specified for children[20] (≤18 years), and 11 articles did not specify age[25 28-37].

[Insert Figure 1 (Legend: Figure 1 PRISMA flow diagram that charts the study identification process)]

For peer review only - http://bmjopen.bmj.com/site/about/guidelines.xhtml

Table 1 Characteristics of the included articles

Author(s)	Date	Aim	Country Origin	Design	Total N	Physical Condition
Al Rajeh et al[15]	2019	Explore the use of telehealth and assess the perceptions of clinicians employing telehealth and specifically alarm settings on technology	UK	Cross-sectional	65	Chronic obstructive pulmonary disease
Amatya et al[26]	2015	Investigate the effectiveness and safety of telerehabilitation	Europe and USA	Systematic review	564	Multiple sclerosis
Anton et al[17]	2018	Develop and examine the Kinext Telerehabilitation System for physical conditions	Spain and Australia	Feasibility	18	Shoulder disorder; Hip replacement
Block et al[27]	2016	Perform a systematic review of studies using remote physical activity monitoring in neurological diseases	USA	Systematic review	943	Neurological diseases
Calvaresi et al[18]	2019	Examine the feasibility of using Artificial Intelligence for telerehabilitation	Italy	Mixed-methods	10	General physical conditions
Giesbrecht & Miller[22]	2019	Evaluate the effect of a mHealth wheelchair skills training program on clinical outcomes among older adult manual wheelchair clients	Canada	RCT	18	Physical disability needing wheelchair
Grau-Pellicer et al[23]	2020	Investigate the effectiveness of a mHealth app	Spain	RCT	41	Ischaemic or haemorrhagic stroke
Guerra de Oliveira Gondim et al[24]	2017	Evaluate the effects of individualized orientation and monitoring by telephone in a self-supervised home therapeutic exercise program on signs and symptoms of Parkinson's disease and quality of life	Brazil	RCT	28	Parkinson's disease

For peer review only - http://bmjopen.bmj.com/site/about/guidelines.xhtml

Hasenohrl et al[19]	2020	Assess the feasibility and acceptance of orthopaedists prescribing individualised therapeutic exercises via a smartphone app to patients	Austria	Qualitative	27	Back pain
Levac et al[20]	2018	Develop and conduct a usability evaluation of the Fun, Interactive Therapy Board (FITBoard), a movement toy for children with disabilities	USA	Observational	7	General movement conditions
Teleswallowing Ltd[31]	2020	Provide evidence-based guidance on designing and establishing telerehabilitation services for dysphagia	UK	Guidance	N/A	Dysphagia
Mani et al[28]	2017	Systematically explore and summarise the validity and reliability of a telerehabilitation -based physiotherapy assessment	Malaysia	Systematic review	NS	MSK conditions
Middleton et al[16]	2020	Describe the process and cost of delivering a synchronous telehealth exercise programme	USA	Observational	1	Stroke, hypertension, and diabetes
Paneroni et al[21]	2015	Test the feasibility, adherence and satisfaction of a home-based reinforcement telerehabilitation programme	Italy	Observational	36	Chronic obstructive pulmonary disease
Charted Society of Physiotherapy[33]	2020	Provide guidance for physiotherapists on digital tools to support service delivery	UK	Guidance	N/A	General physical conditions
Charted Society of Physiotherapy[32]	2020	Provide guidance for physiotherapists on telephone triage of patients with musculoskeletal conditions	UK	Guidance	N/A	MSK conditions

For peer review only - http://bmjopen.bmj.com/site/about/guidelines.xhtml

Charted Society of Physiotherapy[34]	2020	Provide practical advice for physiotherapists and support workers to implement remote consultations	UK	Guidance	N/A	General physical conditions
Royal College of Speech and Language Therapists[36]	2020	Provide brief tips for telerehabilitation implementation revolved around security	UK	Guidance	N/A	General physical conditions
East Lancashire Hospitals NHS Trust[37]	2020	Provide brief tips for telerehabilitation implementation for dysphagia	UK	Guidance	N/A	Dysphagia
Venkataraman et al[25]	2016	Compare Berg Balance Scale ratings using videos with differing transmission characteristics with direct in-person rating	USA	Psychometrics study	45	General motor control condition
Health and Social Care Board NI[30]	2020	Amalgamate evidence and guidance on the conduct of virtual consultations so that it is easily accessible for AHPs	UK	Guidance	N/A	General physical conditions
AHPScot[29]	2020	Guide AHPs in NHS Scotland during remote consultations (telephone or video) with MSK patients during the current COVID19 pandemic	UK	Guidance	N/A	MSK conditions
Royal College of Occupational Therapists[35]	2020	Guide occupational therapists regarding implementation of virtual assessments and reviews	UK	Guidance	N/A	General physical conditions

Grey highlighted rows indicate a guidance document
N/A = Not applicable
NS = Not specified
AHPs = Allied health professionals
MSK = Musculoskeletal
UK = United Kingdom
USA = United States of America

For peer review only - http://bmjopen.bmj.com/site/about/guidelines.xhtml

The 14 included research studies and systematic reviews had the following general aims: one study assessed use and perceptions of telerehabilitation[15]; one study examined costs of implementing telerehabilitation[16]; one study assessed the development of a telerehabilitation technology[17]; three studies systematically reviewed telerehabilitation literature[26-28]; four studies assessed the feasibility of a telerehabilitation technology[18-21]; and four studies examined the effectiveness of the telerehabilitation technology[22-25].

Contexts

Client setting was reported in 13 of the 23 articles, where only one article did not include a home setting and instead only used general clinical sites[20] (Table 2). This article[20] described the development and preliminary usability, from the provider's perspective, of a tablet-based interactive movement tool. Provider setting was reported in six articles and all were telerehabilitation technologies used by providers in secondary care (e.g. an out-patient clinic;[17 19 21-23 25]). The client support environment (i.e. who was with the client) was reported in 13 articles (Table 2), where most clients were alone (n=5). Only one article specifically reported that carers were present during the consultation or intervention. Table 3 shows the wide range of providers using telerehabilitation in people with physical disability, most being a physiotherapist. Of note, all non-qualified assistants were accompanied by a qualified health professional throughout the telerehabilitation sessions.

For peer review only - http://bmjopen.bmj.com/site/about/guidelines.xhtml

Table 2 Cross-tabulation of Client Support and Client Setting information extracted from included articles

			**Client Support Environment**			
		Article Type	*Not specified*	*Client with group*	*Client alone*	*Client with carer*
**Client Setting**	*Not specified*	Primary Research	2[15 17]	-	1[25]	-
		Reviews	-	1[28]	1[28]	-
		Guidance Documents	6[31 32 34-37]	-	-	-
	At home	Primary Research	-	-	4[16 19 21 22]	1[24]
		Reviews	-	-	-	-
		Guidance Documents	-	-	-	-
	Home and community	Primary Research	1[18]	-	-	-
		Reviews	-	2[26 27]	2[26 27]	-
		Guidance Documents	1[33]	2[29 30]	2[29 30]	-
	General clinic sites	Primary Research	1[20]	-	-	-
		Reviews	-	-	-	-
		Guidance Documents	-	-	-	-
	Rehabilitation unit and at home	Primary Research	-	1[23]	-	-
		Reviews	-	-	-	-
		Guidance Documents	-	-	-	-

*Four articles (2 reviews[26 27] and 2 guidance documents[29 30]) had participants complete the telerehabilitation alone and in a group - these articles have been added to both the “Client was Alone” and “Client with Group” categories.

For peer review only

Table 3 List of telerehabilitation administrators extracted from included articles

Telerehabilitation Administrator*	N[a]	N[b]	N[c]
Physiotherapist	9[15-18 20 21 23-25]	1[28]	1[34]
Occupational therapist	3[20 22 25]	-	1[35]
Medic	3[15 19 21]	1[27]	-
Nurse	2[15 21]	-	-
Speech and language therapist	-	-	2[31 37]
General clinicians	-	-	2[32 33]
AHPs	-	-	2[29 30]
Not specified	1[36]	1[26]	-
Assistants	2[16 25]	-	-
Physiologist	1[15]	-	-

AHP = Allied Health Professional, specific profession not stated
*Telerehabilitation administrators overlap in articles, therefore total N does not sum to total included articles
[a]N for primary research studies
[b]N for systematic review studies
[c]N for guidance documents

Presence of COVID-19

Only articles published in 2020[16 19 23 29-37] (n = 12) were considered for COVID-19-specific information. Of these, two articles refer to COVID-19-related management; guidance documents developed by AHPScot [29] and the Health and Social Care Board Northern Ireland[30]. The COVID-19-related recommendations from these guides are detailed in the Recommendations section. It is noteworthy that these guidance documents refer to COVID-19 in general and are not specifically related to telerehabilitation use in people with movement impairment, despite these documents generally considering the use of telerehabilitation for this purpose. Teleswallowing Ltd.[31], Middleton et al[16], and two articles from the Charted Society of Physiotherapy[33 34] also refer to COVID-19 but only state that telerehabilitation is useful to decrease the spread of COVID-19; these articles do not refer to management of patients with COVID-19.

Types of Telerehabilitation

Table 4 shows the aims of the telerehabilitation interventions examined in the primary research studies. Nine guides[29-37], 3 systematic reviews[26-28], and 2 primary studies[18 25] are excluded from Table 4 as they did not evaluate a specific telerehabilitation technology. Most (5 of the 9 studies) telerehabilitation interventions aimed to assist in management of a movement condition; however, some cited additional aims such as optimising adherence[15], improving quality of life[24], monitoring, and assessing client satisfaction[23]. One intervention provided training in using a wheelchair[22].

We aimed to summarise the various platforms used for delivery of telerehabilitation but this proved difficult, as some articles did not have sufficient detail. For example, one article[16] reports a tablet app but does not specify whether video was involved or whether it just used a chat/telephone function. Accepting these limitations, the most common delivery system appeared to be via video (e.g. video call, Skype, video-recording, etc.), followed by apps (on tablets or mobiles) and telephone calls. Fifteen of the telerehabilitation platforms were synchronous[16 17 19 20 22-24 28-31 33 35-37] (i.e. in real-time) and seven

For peer review only - http://bmjopen.bmj.com/site/about/guidelines.xhtml

platforms used both synchronous and asynchronous[15 18 21 26 27 32 34] (i.e. not in real-time) methods. Only one article used only an asynchronous platform where providers assessed physical ability from a video-recording of the client[25].

Table 4 List of telerehabilitation aims and platforms reported in the included articles

Telerehabilitation Aim	N	References
MCM	5	[16 17 19-21]
MCM and adherence	1	[15]
MCM and quality of life	1	[24]
MCM and client satisfaction	1	[23]
Training for assistive device use	1	[22]

MCM = Movement condition management

Of the 11 primary research studies, five[15 17-19 22] specified neither number of sessions nor session duration, one specified just number and one specified just duration. The number of sessions ranged from 1 to 36 (n = 2 for 1 session[20 25]; n = 1 for 16 sessions[23]; n = 1 for 28 sessions[21]; n = 2 for 36 sessions[16 24]). Three studies included 60 minute sessions[20 23 24], one included 50 minute sessions[16], and one included 100 minute sessions[21]. The variability seen here is reflective of that seen in face-to-face rehabilitation sessions.

Guidance

Specific guidance on how to use telerehabilitation technology was supplied to providers in 12 articles[20 22 25 29-37], of which three were studies[20 22 25] and the remaining were guidance documents[29-37]. A guidebook or manual was provided in ten articles[20 22 25 29-31 33-35 37], while two provided a checklist[32 36]. No guidance was supplied in seven articles[15 16 19 21 23 24], and five studies did not mention whether guidance was provided or not[17 18 26-28].

Training

Guidance documents and systematic reviews were not included in training analysis as these articles had no participants to whom to supply training. Specific training on how to use telerehabilitation technology was supplied to providers in 4 out of 11 primary research studies[16 20 22 25]. The training was provided in-person in three of these studies [16 20 22], while the remaining study provided training via a video tutorial[25]. No training was supplied in five studies[15 19 21 23 24], and 2 studies did not mention training[17 18].

Recommendations

Six themes and 11 subthemes were identified within the recommendations: Administrative Advice, Challenges, Governance, Social Support, Technology Improvements, and COVID-19 (see Table 5). Recommendations were also analysed to extract procedural guidance that providers can perform in preparation for, during, and after a telerehabilitation session. This guidance is summarised in supplementary materials (SM3) as it extended the review beyond its key message.

For peer review only - http://bmjopen.bmj.com/site/about/guidelines.xhtml

Table 5 Summary of themes identified during qualitative analysis of article recommendations

Analysis Themes	Description
Administrative Recommendations<br>▪ General<br>▪ Client-Specific<br>▪ Technology-Specific<br>▪ Data Security	Advice revolved around administrative duties, such as record keeping, technology maintenance, or data security
Challenges<br>▪ Care Provision<br>▪ Social Factors<br>▪ Technology Issues	Challenges that providers may encounter when conducting telerehabilitation services
Governance	Ethical advice, such as client consent and/or assessing confidentiality
Support Mechanisms<br>▪ Providers<br>▪ Carers<br>▪ Provider Assistants<br>▪ Non-Clinical Staff	Advice that emphasised the important role of support from various sources involved in providing telerehabilitation care to the client
Technology Improvements	Suggestions that may improve the use and function of a telerehabilitation technology
COVID-19	Actions providers are advised to conduct to address COVID-19 risks

Administrative Recommendations

Three articles[30 32 36] provided advice on administrative duties that providers should perform when conducting telerehabilitation. The advice revolved around four sub-themes:

(1) General Advice involved general administrative duties such as maintaining record-keeping standards and identifying the specific resources a provider will need when engaging in telerehabilitation (e.g. number of sessions needed, appropriate length of session considering technology set-up, clinical outcome measures needed, etc.).

(2) Client-Specific Advice involved duties that required interactions with the client, such as documenting all client conversations and noting care that would have been provided if the intervention was delivered face to face but was not done so due to the restrictions of telerehabilitation.

(3) Technology-Specific Advice involved ensuring technology was up-to-date and that the latest version of any software was being used.

(4) Data Security emphasised that providers must minimise identifiable information that is shared digitally, ensure that all actions comply with information governance, and only use technology recognised by the institute through which they provide their care services.

Challenges

Six articles[15 19 23 25 28 30] highlighted challenges when conducting telerehabilitation, which involved three sub-themes:

(1) Care Provision considers the resources needed for telerehabilitation, highlighting that some clinical assessments may be too complicated to conduct effectively at a distance and that, if this complication is not recognised by inexperienced telerehabilitation providers, the quality of care provided may be reduced.

For peer review only - http://bmjopen.bmj.com/site/about/guidelines.xhtml

(2) Social Factors identified social challenges, where unsatisfactory video etiquette, minimal rapport-building, and socio-economic disadvantages have the potential to reduce the quality of care provided via telerehabilitation.
(3) Technology Issues relate to technology failures or interruptions, such as inadequate internet connection, low camera/audio quality, and false alarms (on wearable devices) that result in a reduced quality of care.

Governance
Six articles[29 30 34-37] provided recommendations relating to governance issues. The guidance highlighted that all clients must be informed about the following: telerehabilitation is voluntary; potential risks when engaging in telerehabilitation; what will happen with their data; and what reasonable actions they should take to ensure the security of their technology. This theme also emphasised the importance of consent, where informed consent must be taken explicitly from clients when video/audio recording or taking screen shots. If required, clients must be asked whether they consent for their trusted others (e.g. carer or family) to attend the session with them. However, one article [30] stated that consent was implied when clients enter the telerehabilitation session.

Support Mechanisms
Six articles[21 23 24 28 31 37] recommended support mechanisms to be set in place for clients when providing telerehabilitation. This support involved four sub-themes:
(1) Providers to offer support beyond the main goals of telerehabilitation, such as reassurance to clients who are anxious using technology for their rehabilitation.
(2) Carers to offer clients in-person support during delivery of telerehabilitation sessions. For instance, provision of physical assistance to safely enable the client to achieve correct posture or movement, or to assist the client with using the technology, such as positioning of the camera.
(3) Provider Assistants to assist qualified staff by visiting clients in person to assist telerehabilitation, such as undertaking outcome measures face-to-face, when required and safe to do so.
(4) Non-Clinical Staff, such as administrators and senior management, to provide essential support in successful coordination and implementation of telerehabilitation, emphasising the importance of effective communication between support staff and providers.

Technology Improvements
Eight articles[15 17-20 26-28] recommended technology improvements for telerehabilitation purposes. For instance, communications should be synchronous and technology must have a client-friendly interface that satisfies both the client and the provider. Wearable technologies relevant to posture and movement (e.g. to measure steps or heart rate) must be precise. Technology would especially benefit from improved monitoring capabilities, such as real-time movement tracking and activity adherence monitoring. If technology provided smart data (e.g. real-time measures of muscle strength), it should be accompanied by useful and actionable information. Reminders to engage in self-care activities would be useful for clients during their rehabilitation period. A built-in anatomical map to assist clients in showing the location of pain, tenderness, or other sensations was also suggested.

COVID-19
Two articles (both guidance documents[29 30]) made recommendations to address COVID-19 concerns. When establishing whether the client needs telerehabilitation or face-to-face care, professionals were advised to identify the client's COVID-19 status. Suggested identifications included: tested positive and self-isolating, has symptoms and self-isolating, has come into contact with someone who tested positive and is now self-isolating, shielding, or no current cautions outside of government restrictions. Of note, this is an area of rapid expansion in the literature.

For peer review only - http://bmjopen.bmj.com/site/about/guidelines.xhtml

DISCUSSION

The 23 articles included in this rapid scoping review revealed two main gaps in the literature: (1) telerehabilitation guidance (from research studies and guidance documents) is not specific to movement-related rehabilitation despite articles stating this explicitly as their aim, and (2) neither guidance nor training were given to providers in most research studies evaluating a telerehabilitation technology.

The Lack of Specific Guidance

The vast majority of the existing recommendations relate predominately to communication or governance elements of telerehabilitation, and lack specific guidance about issues pertinent to movement impairment. Several elements that are key to effectively implementing telerehabilitation for people with physical disability are missing. When assessing movement remotely, one would expect guidance on ensuring patient safety, triaging patients, and specific steps to decide whether a patient needs face-to-face care. More importantly, the identified guidance does not effectively address the limitations of undertaking physical assessments remotely; for example, if the patient is unable to move their body themselves if their movement is inadequate for assessment. Remote physical examinations are complex and need specific guidance on implementation; this is reflected in recent research that highlights physical examinations as one of the main challenges for remote assessments[38 39]. There was also minimal information on telerehabilitation related to COVID-19. This is unsurprising given the novelty of this infection. Overall, current telerehabilitation guidance for physical disabilities focuses on general tele-health guidance rather than being specific to movement impairments. Although issues such as data security, video etiquette, and organisation are important, they are not enough to assist providers in effective telerehabilitation implementation for people with movement impairments.

The Minimal Focus on Providers

Of the 11 included primary studies and 3 systematic reviews, only three[20 22 25] provided guidance to providers. Similarly, only four research studies[16 20 22 25] described the provision of training to providers. Neglecting providers' skills in using technology effectively (i.e. digital literacy) potentially undermines the usefulness of any telerehabilitation technology that was evaluated. Furthermore, studies mainly report the clients' and not the providers' context of using telerehabilitation (e.g. whether the provider was from primary, secondary, or tertiary care). The generalisability of the telerehabilitation technology examined in the study is thus unclear, as certain technologies may only be effective in particular contexts[40]. Future studies should carefully consider providers' digital literacy and context to better understand the effectiveness of telerehabilitation technology. Digital literacy and context are not the only important factors for telerehabilitation implementation. Palacholla et al's[41] scoping review revealed that healthcare providers need appropriate organisational infrastructures (e.g. effective IT support) and a sufficient workflow integration to effectively implement technology. They also reported that an easy-to-use interface is important, which aligns with the review's finding of needing a friendly client interface for technological improvements.

Digital Equity

Although digital equity was not part of this review's objectives, it is important to address. The included articles provide information based on the assumption that all clients (and providers) have both access and skills to engage in technology for remote assessments. Many of those currently digitally excluded are likely to become increasingly neglected from remote health services, thereby exacerbating existing health inequalities[42]. Future studies should acknowledge equity of access when examining telerehabilitation technologies. Future guidance documents should provide information for when clients do not have access to (or decline to use) telerehabilitation.

For peer review only - http://bmjopen.bmj.com/site/about/guidelines.xhtml

Comparisons with Other Studies

To our knowledge, this is the first scoping review of telerehabilitation guidance and training for assessing physical disabilities within daily clinical practice. Previous reviews mostly examine effectiveness within the context of research studies, and conclude that further provider and client training is needed to adequately implement telerehabilitation for physical assessments[43 44]. These studies conclude that telerehabiltiation technology has potential to deliver effective physical disability care, the current state of the art is insufficient for accurate remote movement assessment[45]. Our review confirms the need for further provider training, and additionally emphasises the lack of specific movement-related guidance.

Study Limitations

This scoping review has several limitations. Firstly, many telerehabilitation guidance documents for physical disabilities are likely unpublished. Although this review conducted an extensive search within grey literature sources, guidance documents and other relevant studies have likely been missed. Secondly, the search strategy was biased towards the English language. Despite no language restrictions during the search and the use of language translation software, relevant articles have likely been missed. Finally, this review was restricted by its rapid, cross-sectional approach. The authors acknowledge that some articles within this review may have been updated since the initial search and others have likely emerged since. This review may serve as a foundation for future research and development of guidance documents to provide information that addresses the gaps identified above.

CONCLUSION

There is a clear need for specific telerehabilitation guidance for physical disabilities, particularly for effective practice of remote physical assessments. Studies examining telerehabilitation technologies should deliver guidance and training to providers as well as document the context of providers if those technologies are to be effectively implemented. The catalyst that is the COVID-19 pandemic has forced the uptake of remote rehabilitation services, which are likely to persist beyond the pandemic. There is a pressing need for explicit, detailed telerehabilitation guidance, without which remote physical assessments may result in sub-optimal management.

Acknowledgments

We would like to sincerely thank Chris Johns from the University of Plymouth, who provided library support during the initial phase of this scoping review, and particularly assisted in developing the search strategy. Thanks to Sarah Chatfield who undertook the initial limited search of the literature.

Author Contributions

Krithika Anil conducted the literature search, organised the review team, and produced the initial manuscript. Jenny Freeman conceptualised the study idea and supervised study processes. All authors reviewed the articles from the literature search, extracted data, contributed to data interpretation, and critically evaluated the manuscript. The corresponding author attests that all listed authors meet authorship criteria and that no others meeting the criteria have been omitted.

Funding

This work was supported by UKRI-NIHR (MRC Section), Covid-19; Reference MR/V021060/1. The funder had no role in study design, data collection, data analysis, or writing of the report.

For peer review only - http://bmjopen.bmj.com/site/about/guidelines.xhtml

Competing Interests

None declared.

Data Sharing

No additional data available.

Dissemination to Participants and Related Patient and Public Communities

No participants were involved in this review. The research findings will be disseminated at relevant professional organisation and scientific conferences.

References

1. Peek N, Sujan M, Scott P. Digital health and care in pandemic times: impact of COVID-19. *BMJ Health and Care Informatics* 2020;27(1):e100166.
2. Brennan D, Tindall L, Theodoros D, et al. A blueprint for telerehabilitation guidelines. *Int J Telerehabil* 2010;2(2):31-34. doi: 10.5195/ijt.2010.6063
3. Charted Society of Physiotherapy. Rehabilitation and Covid-19 - CSP policy statement 2020 [Available from: https://www.csp.org.uk/professional-clinical/improvement-innovation/community-rehabilitation/rehab-covid-19-policy-statement.
4. British Society of Rehabilitation Medicine. Rehabilitation in the wake of Covid-19 - A phoenix from the ashes, 2020.
5. Wade DT. The future of rehabilitation in the United Kingdom National Health Service: Using the COVID-19 crisis to promote change, increasing efficiency and effectiveness: SAGE Publications Sage UK: London, England, 2020.
6. Wade DT. What attributes should a specialist in rehabilitation have? Seven suggested specialist Capabilities in Practice: SAGE Publications Sage UK: London, England, 2020.
7. Aromataris E, Munn Z. Chapter 1: JBI Systematic Reviews. In: Aromataris E, Munn Z, eds. JBI Manual for Evidence Synthesis: JBI 2020.
8. Tricco AC, Lillie E, Zarin W, et al. A scoping review on the conduct and reporting of scoping reviews. *BMC medical research methodology* 2016;16(1):15.
9. Peters MDJ, Marnie C, Tricco AC, et al. Updated methodological guidance for the conduct of scoping reviews. *JBI Evid Synth* 2020;18(10):2119-26. doi: 10.11124/jbies-20-00167 [published Online First: 2020/10/11]
10. Meyerson B. Top 10 emerging technologies of 2015 World Economic Forum2015 [Available from: https://www.weforum.org/agenda/2015/03/top-10-emerging-technologies-of-2015-2.
11. Wood L. Global Telepresence Robots Market (2020 to 2025) - Growth, Trends, and Forecast Business Wire2020 [Available from: https://www.businesswire.com/news/home/20201005005668/en/Global-Telepresence-Robots-Market-2020-to-2025---Growth-Trends-and-Forecast---ResearchAndMarkets.com.
12. Reuters T. EndNote X9. 2013
13. Ouzzani M, Hammady H, Fedorowicz Z, et al. Rayyan — a web and mobile app for systematic reviews. *Syst Rev* 2016(5):210. doi: 10.1186/s13643-016-0384-4
14. Kim H, Sefcik JS, Bradway C. Characteristics of Qualitative Descriptive Studies: A Systematic Review. *Res Nurs Health* 2017;40(1):23-42. doi: 10.1002/nur.21768 [published Online First: 2016/09/30]
15. Al Rajeh A, Steiner MC, Aldabayan Y, et al. Use, utility and methods of telehealth for patients with COPD in England and Wales: a healthcare provider survey. *BMJ Open Respir Res* 2019;6(1):e000345.
16. Middleton A, Simpson KN, Bettger JP, et al. COVID-19 Pandemic and Beyond: Considerations and Costs of Telehealth Exercise Programs for Older Adults With Functional Impairments Living at Home-Lessons Learned from a Pilot Case Study. *Phys Ther*
17. Anton D, Berges I, Bermúdez J, et al. A Telerehabilitation System for the Selection, Evaluation and Remote Management of Therapies. *Sensors (Basel)*;18(5)
18. Calvaresi D, Marinoni M, Dragoni AF, et al. Real-time multi-agent systems for telerehabilitation scenarios. *Artif Intell Med*;96:217-31.

For peer review only - http://bmjopen.bmj.com/site/about/guidelines.xhtml

19. Hasenohrl T, Windschnurer T, Dorotka R, et al. Prescription of individual therapeutic exercises via smartphone app for patients suffering from non-specific back pain A qualitative feasibility and quantitative pilot study. *Wien Klin Wochen*;132(5):115-23.
20. Levac D, Dumas HM, Meleis W. A Tablet-Based Interactive Movement Tool for Pediatric Rehabilitation: Development and Preliminary Usability Evaluation. *JMIR Rehabil Assist Technol*;5(2):e10307.
21. Paneroni M, Colombo F, Papalia A, et al. Is Telerehabilitation a Safe and Viable Option for Patients with COPD? A Feasibility Study. *Copd*;12(2):217-25.
22. Giesbrecht EM, Miller WC. Effect of an mHealth wheelchair skills training program for older adults: a feasibility randomized controlled trial. *Archives of Physical Medicine and Rehabilitation 2019 Nov;100(11):2159-2166* 2019
23. Grau-Pellicer M, Lalanza JF, Jovell-Fernandez E, et al. Impact of mHealth technology on adherence to healthy PA after stroke: a randomized study. *Topics in Stroke Rehabilitation 2020;27(5):354-368* 2020
24. Guerra de Oliveira Gondim IT, Cabral dos Santos Accioly Lins C, Jorge Asano NM, et al. Individualized guidance and telephone monitoring in a self-supervised home-based physiotherapeutic program in Parkinson. *Fisioterapia em Movimento [Physical Therapy in Movement] 2017 Jul-Sep;30(3):559-568* 2017
25. Venkataraman K, Morgan M, Amis KA, et al. Tele-Assessment of the Berg Balance Scale: Effects of Transmission Characteristics. *Arch Phys Med Rehabil*;98(4):659-64.e1.
26. Amatya B, Galea MP, Kesselring J, et al. Effectiveness of telerehabilitation interventions in persons with multiple sclerosis: A systematic review. *Mult Scler Relat Disord*;4(4):358-69.
27. Block VA, Pitsch E, Tahir P, et al. Remote Physical Activity Monitoring in Neurological Disease: A Systematic Review. *PLoS One* 2016;11(4):e0154335.
28. Mani S, Sharma S, Omar B, et al. Validity and reliability of Internet-based physiotherapy assessment for musculoskeletal disorders: a systematic review. *J Telemed Telecare*;23(3):379-91.
29. AHPScot. A guide to Musculoskeletal (MSK) Digital Health consultations during Coronavirus (COVID-19), 2020.
30. Health and Social Care Board. AHP Virtual Consultation Guidance, 2020.
31. Teleswallowing Ltd. Telehealth Service Delivery Guide, 2020.
32. Charted Society of Physiotherapy. Telephone guidance for musculoskeletal practice, 2020.
33. Charted Society of Physiotherapy. Digital tools to support service delivery, 2020.
34. Charted Society of Physiotherapy. COVID-19: guide for rapid implementation of remote consultations, 2020.
35. Royal College of Occupational Therapists. Virtual Assessments/Reviews: The Script. 2020
36. Royal College of Speech and Language Therapists. MAINTAINING SECURITY FOR TELEHEALTH SESSIONS, 2020.
37. East Lancashire Hospitals NHS Trust. Telepractice dysphagia assessmentsGuidelines for use with a patient in their own home/nursing home, 2020.
38. Seuren LM, Wherton J, Greenhalgh T, et al. Physical Examinations via Video for Patients With Heart Failure: Qualitative Study Using Conversation Analysis. *J Med Internet Res* 2020;22(2):e16694. doi: 10.2196/16694
39. Shaw SE, Seuren LM, Wherton J, et al. Video Consultations Between Patients and Clinicians in Diabetes, Cancer, and Heart Failure Services: Linguistic Ethnographic Study of Video-Mediated Interaction. *J Med Internet Res* 2020;22(5):e18378. doi: 10.2196/18378
40. Shaw J, Shaw S, Wherton J, et al. Studying Scale-Up and Spread as Social Practice: Theoretical Introduction and Empirical Case Study. *J Med Internet Res* 2017;19(7):e244. doi: 10.2196/jmir.7482
41. Palacholla RS, Fischer N, Coleman A, et al. Provider- and Patient-Related Barriers to and Facilitators of Digital Health Technology Adoption for Hypertension Management: Scoping Review. *JMIR cardio* 2019;3(1):e11951-e51. doi: 10.2196/11951
42. Majeed A, Maile EJ, Coronini-Cronberg S. Covid-19 is magnifying the digital divide: BMJ; 2020 [Available from: https://blogs.bmj.com/bmj/2020/09/01/covid-19-is-magnifying-the-digital-divide/ accessed Dec 2020.
43. Hosseiniravandi M, Kahlaee AH, Karim H, et al. Home-based telerehabilitation software systems for remote supervising: a systematic review. *International Journal of Technology Assessment in Health Care* 2020;36(2):113-25.
44. Peretti A, Amenta F, Tayebati SK, et al. Telerehabilitation: Review of the State-of-the-Art and Areas of Application. *JMIR rehabilitation and assistive technologies* 2017;4(2):e7.

For peer review only - http://bmjopen.bmj.com/site/about/guidelines.xhtml

45. Aderonmu JA. Emerging challenges in meeting physiotherapy needs during COVID-19 through telerehabilitation. *Bulletin of Faculty of Physical Therapy* 2020;25(1):16. doi: 10.1186/s43161-020-00018-4

For peer review only

For peer review only - http://bmjopen.bmj.com/site/about/guidelines.xhtml

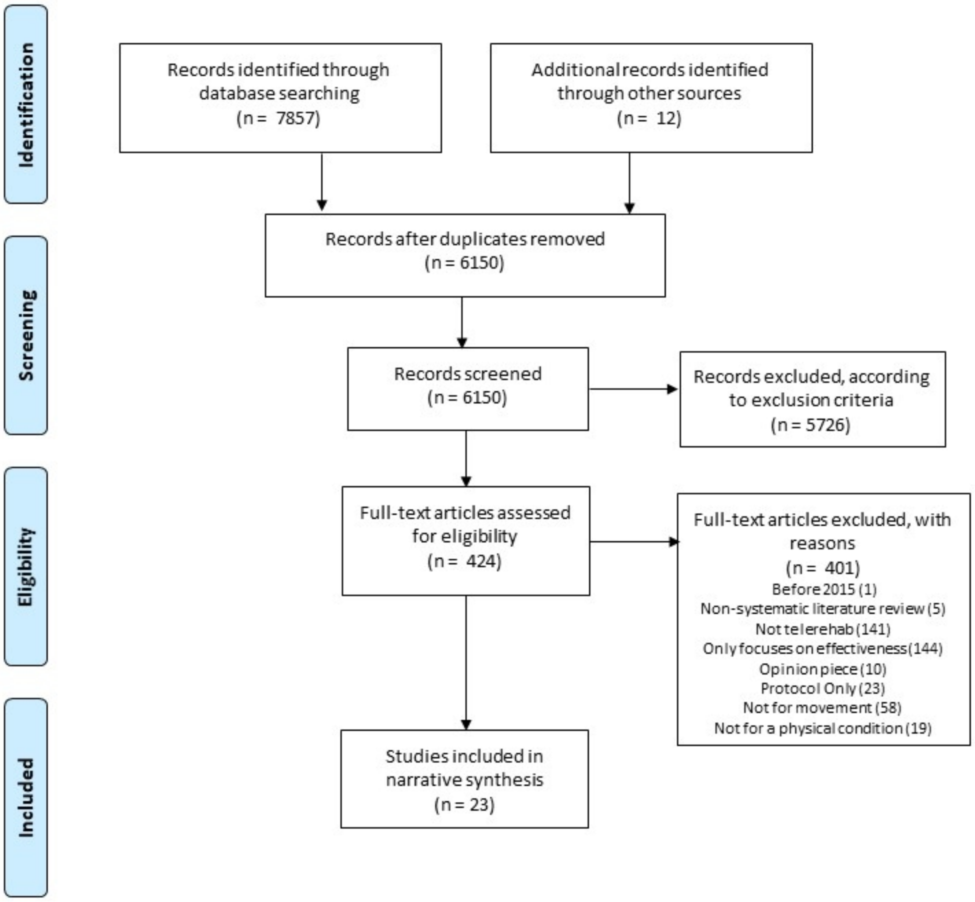

PRISMA flow diagram that charts the study identification process

192x173mm (96 x 96 DPI)

Protocol for a Rapid Scoping Review on Telerehabilitation

Review question and objectives

This review aims to answer the following question: what guidance and training is available to implement telerehabilitation movement assessment for people (minors and adults) with a physical disability, including those recovering from Covid-19? The following objectives will aid in answering the aforementioned review question:

- To identify the contexts, types of telerehabilitation assessments delivered in a health and social care setting for people with a disability (including those recovering from Covid-19)
- To identify any guidance and/or training available for delivering telerehabilitation assessments
- To identify specific recommendations related for effective implementation of telerehabilitation guidance

Methods

Inclusion Criteria

The inclusion criteria was developed using the PCC (participant, concept, context) framework.

Participants

The current review will consider studies that included participants with a physical disability (PD); this review does not have an age restriction. This review defines PD as any physical condition that affects a person's mobility, physical capacity, stamina, or dexterity; this includes PD originating from the aging process. The review will include studies of participants with a PD who have any duration and severity of PD.

Concept

This review will consider studies and policies that examine or describe guidance and training regarding implementation of telerehabilitation movement assessments. Guidance includes checklists, tools, outcome measurement batteries, protocols, guidelines, risk assessments, exemplars, and appraisals. Training includes interactions, communications, and resources with the aim to improve and/or inform the process of delivering telerehabilitation. Guidance and training must involve movement evaluation, that is, the evaluation of how human beings move around, perform, and function. This includes self-administrated and health professional administrated telerehabilitation. Studies and policies that do not involve movement evaluation will be excluded from this review.

Context

This review will consider studies that provide guidance and training in healthcare and social care contexts, that is, telerehabilitation must be provided by a healthcare or social care professional, system, or institute.

Types of Literature

This review will consider the following study designs: randomized controlled trials (RCTs), non-RCTs, quasi-experimental, pre- and post- studies, case studies, observational studies, systematic reviews, and qualitative studies. Grey literature regarding policies from recognized UK-based national and international institutes will also be considered. All studies and policies must be in the English language as team members are unable to translate other languages. Opinion pieces, non-systematic reviews, and other grey literature will be excluded.

Search Strategy

A three-step search strategy will be utilized. An initial limited search of PubMed and CINHAL was undertaken to estimate the volume of relevant literature and to identify key words to assist in developing search terms. A second search using the developed search terms will be undertaken and adapted across each included information source (see Information Sources below). The third strategy involves searching for additional studies within the reference list of all studies that met the inclusion criteria. Due to the rapid nature of this review, study authors will not be contacted for further information in cases of ongoing or uncompleted studies. Please see Appendix I for search strategy terms.

Information Sources

The databases to be searched are:

- PubMed
- CINHAL

For peer review only

- PsychoInfo
- Cochrane
- EMBASE
- Web of Science
- PEDro
- UK Health Forum
- WHO
- National Archives
- Public Health England

Study Selection

Following the search, all identified references will be imported into EndNote (a software that allows easy organisation of study references, including study titles and abstracts). After removal of duplications within EndNote, references will be uploaded to the online Raayan tool; this tool is designed specifically for the organisation of studies for a systematic review and can also be used to coordinate study selection amongst review team members. Titles and abstracts will be screened by two independent reviewers for assessment against the inclusion criteria for the review. The full text of potentially eligible studies will be retrieved and assessed in detail against the inclusion criteria by two independent reviewers. Full text studies that do not meet the inclusion criteria will be excluded and will be displayed in a PRISMA flowchart. Reasons for their exclusion will be provided in the appendix of the full systematic review. Any disagreements that arise between the reviewers will be resolved through discussion and with a third reviewer.

Assessment of Methodological Quality

Eligible studies will be critically appraised by two independent reviewers for methodological quality using the standardized critical appraisal instruments from the Joanna Briggs Institute. Any disagreements will be resolved through discussions, and through a third reviewer if necessary. All studies, regardless of their methodological quality, will be included for data extraction and synthesis, where possible. The results of critical appraisal will be reported in narrative and tabular form.

Data Extraction

Data extraction of included studies will be conducted by two independent reviewers. Narrative data extraction will be conducted using an Excel spreadsheet. The following data will be extracted for the narrative synthesis: [TBC when excel spreadsheet is done]. Any disagreements that arise between the reviewers will be resolved through discussion, or with a third reviewer.

Data Synthesis

The narrative synthesis of the findings from included studies will be structured according to the review objectives. Potential subgroups will be based on the study quality, age, synchronous and asynchronous telerehabilitation, telerehabilitation when recovering from COVID-19, and potential barriers. The findings will be presented narratively aided by appropriate tables and figures.

For peer review only

Appendix I: Search Strategy Terms

The below search strategy uses a logic grid and is based on the PCC framework; additional columns were added to aid in search sensitivity. As advised by an expert librarian, the third column will only be used to narrow searches that produce an overwhelming quantity of results.

Population 1	*(Population 2)*	*Concept 1*	*Concept 2*	*Concept 3*	*Context*
Physical condition	Covid*	Tele*	Training	Movement	Healthcare
Physical disability	Coronavirus	Virtual	Guidance	Mobility	Health care
Physical impairment	2019-nCoV	Online	Assessment	Function	Clinic
	SARS-CoV-2	Video	Tool*	Gait	Social care
	Severe acute respiratory syndrome coronavirus 2	Digital	Protocol	Walking	Hospital
		Multimedia	Battery	Balance	Practice
		Mobile		Motor	
		Phone		Strength	
		mHealth		Power	
		eHealth		Tone	
				Range	
				Contracture	
				Fatigue	
				Stamina	
				Dexterity	
				Physical capacity	

Search term strategy:

1. Each column will be searched individually, e.g. physical condition OR physical disability OR physical impairment
2. All individual searches based on each column will be combined, e.g. (physical condition OR physical disability OR physical impairment)AND (Tele* OR Virtual OR Online OR Video OR Digital OR Multimedia OR Mobile OR Phone OR mHealth OR eHealth)
3. The search strategy will proceed as follows (after searching for each column individually):
   a. Column 1 AND column 3 AND column 4 AND column 5
   b. If there are too many hits based on a., column 6 will be added
   c. Column 2 will only be used as an add-on after searches to keep a separate record of covid-related literature for the narrative synthesis
4. Unpublished literature searches will use a combination of column 1 and 3 in the first instance, where searches will be tailored according to individual database searching tools.
5. Date limiter: 2015 onwards.

For peer review only

Search Strategy Terms

The below search strategy uses a logic grid and is based on the PCC framework; additional columns were added to aid in search sensitivity. As advised by an expert librarian, “concept 3” column was only used to narrow searches that produced an overwhelming quantity of results.

Population 1	*(Population 2)*	*Concept 1*	*Concept 2*	*Concept 3*	*Context*
Physical condition	Covid*	Tele*	Training	Movement	Healthcare
Physical disability	Coronavirus	Virtual	Guidance	Mobility	Health care
Physical impairment	2019-nCoV	Online	Assessment	Function	Clinic
	SARS-CoV-2	Video	Tool*	Gait	Social care
	Severe acute respiratory syndrome coronavirus 2	Digital	Protocol	Walking	Hospital
		Multimedia	Battery	Balance	Practice
		Mobile		Motor	
		Phone		Strength	
		mHealth		Power	
		eHealth		Tone	
				Range	
				Contracture	
				Fatigue	
				Stamina	
				Dexterity	
				Physical capacity	

Search term strategy:

1. Each column was searched individually, e.g. physical condition OR physical disability OR physical impairment
2. All individual searches based on each column were combined, e.g. (physical condition OR physical disability OR physical impairment)AND (Tele* OR Virtual OR Online OR Video OR Digital OR Multimedia OR Mobile OR Phone OR mHealth OR eHealth)
3. The search strategy proceeded as follows (after searching for each column individually):
    a. Column 1 AND column 3 AND column 4 AND column 5
    b. If there were too many hits based on a., column 6 wasto be added

For peer review only

   c. Column 2 was only to be used as an add-on after searches to keep a separate record of covid-related literature for the narrative synthesis
4. Unpublished literature searches used a combination of column 1 and 3 in the first instance, where searches were tailored according to individual database searching tools.
5. Date limiter: 2015 onwards.

For peer review only

Record of Online Searches

Database: PubMed
Date: 20/07/20

ID	Search Term(s)	Hits
1	(physical condition) OR (physical disability) OR (physical impairment)	641,602
2	(tele*) OR (virtual) OR (online) OR (video) OR (digital) OR (multimedia) OR (mobile) OR (phone) OR (mhealth) OR (ehealth)	862,859
3	(training) OR (guidance) OR (assessment) OR (tool*) OR (protocol) OR (battery)	5,791,855
4	(movement) OR (mobility) OR (function) OR (gait) OR (walking) OR (balance) OR (motor) OR (strength) OR (power) OR (tone) OR (range) OR (contracture) OR (fatigue) OR (stamina) OR (dexterity) OR (physical capacity)	15,600,817
5	(ID 1) AND (ID 2) AND (ID 3) AND (ID 4) with date limiter: 2015 onwards	5,322

Database: CINHAL
Date: 03/08/20

ID	Search Term(s)	Hits
1	(physical condition) OR (physical disability) OR (physical impairment)	52,385
2	(tele*) OR (virtual) OR (online) OR (video) OR (digital) OR (multimedia) OR (mobile) OR (phone) OR (mhealth) OR (ehealth)	267,357
3	(training) OR (guidance) OR (assessment) OR (tool*) OR (protocol) OR (battery)	1,100,793
4	(movement) OR (mobility) OR (function) OR (gait) OR (walking) OR (balance) OR (motor) OR (strength) OR (power) OR (tone) OR (range) OR (contracture) OR (fatigue) OR (stamina) OR (dexterity) OR (physical capacity)	902,661
5	(ID 1) AND (ID 2) AND (ID 3) AND (ID 4) with date limiter: 2015 onwards	197

Database: PsychInfo
Date: 03/08/20

ID	Search Term(s)	Hits
1	(physical condition) OR (physical disability) OR (physical impairment)	148,089
2	(tele*) OR (virtual) OR (online) OR (video) OR (digital) OR (multimedia) OR (mobile) OR (phone) OR (mhealth) OR (ehealth)	240,422

For peer review only

3	(training) OR (guidance) OR (assessment) OR (tool*) OR (protocol) OR (battery)	1,057,337
4	(movement) OR (mobility) OR (function) OR (gait) OR (walking) OR (balance) OR (motor) OR (strength) OR (power) OR (tone) OR (range) OR (contracture) OR (fatigue) OR (stamina) OR (dexterity) OR (physical capacity)	1,159,877
5	(ID 1) AND (ID 2) AND (ID 3) AND (ID 4) with date limiter: 2015 onwards	557

Database: Cochrane Library (Reviews Only)
Date: 03/08/20

ID	Search Term(s)	Hits
1	(physical condition OR physical disability OR physical impairment) AND<br>(tele* OR virtual OR online OR video OR digital OR multimedia OR mobile OR phone OR mhealth OR ehealth) AND<br>(training OR guidance OR assessment OR tool* OR protocol OR battery) AND<br>(movement OR mobility OR function OR gait OR walking OR balance OR motor OR strength OR power OR tone OR range OR contracture OR fatigue OR stamina OR dexterity OR physical capacity)<br>with date limiter: 2015 onwards	17

Database: EMBASE
Date: 05/08/20

ID	Search Term(s)	Hits
1	physical condition OR physical disability OR physical impairment	31,388
2	tele* OR virtual OR online OR video OR digital OR multimedia OR mobile OR phone OR mhealth OR ehealth	973,017
3	training OR guidance OR assessment OR tool* OR protocol OR battery	457,1408
4	movement OR mobility OR function OR gait OR walking OR balance OR motor OR strength OR power OR tone OR range OR contracture OR fatigue OR stamina OR dexterity OR physical capacity	700,1843
5	(ID 1) AND (ID 2) AND (ID 3) AND (ID 4) with date limiter: 2015 onwards	157

Database: Web of Science
Date: 05/08/20

For peer review only

ID	Search Term(s)	Hits
1	TS=(physical condition OR physical disability OR physical impairment)	277,584
2	TS=(tele* OR virtual OR online OR video OR digital OR multimedia OR mobile OR phone OR mhealth OR ehealth)	2,360,472
3	TS=(training OR guidance OR assessment OR tool* OR protocol OR battery)	4,974,907
4	TS=(movement OR mobility OR function OR gait OR walking OR balance OR motor OR strength OR power OR tone OR range OR contracture OR fatigue OR stamina OR dexterity OR physical capacity)	12,757,447
5	(ID 1) AND (ID 2) AND (ID 3) AND (ID 4) with date limiter: 2015 onwards	1,720

Database: PEDro
Date: 06/08/20

ID	Search Term(s)	Hits
1	Tele* AND training with date limiter: 2015 onwards	79
2	Tele* AND guidance with date limiter: 2015 onwards	12
3	mHealth AND training with date limiter: 2015 onwards	8
4	mHealth AND guidance with date limiter: 2015 onwards	1
5	eHealth AND training with date limiter: 2015 onwards	1
6	eHealth AND guidance with date limiter: 2015 onwards	0

Database: UK Health Forum
Date: 11/08/20

ID	Search Term(s)	Hits
1	Tele	0
2	Telehealth	0
3	Telemedicine	0
4	Telerehabilitation	0
5	Tele rehabilitation	0
6	mhealth	0
7	ehealth	1

For peer review only

8	Virtual	0
9	Online	4
10	Video	0
11	Digital	0
12	Multimedia	0
13	Mobile	0
14	Phone	0

Database: National Archives (UK)
Date: 06/08/20

ID	Search Term(s)	Hits
1	Tele* with date limiter: 2015 onwards	37
2	mhealth with date limiter: 2015 onwards	0
3	ehealth with date limiter: 2015 onwards	1

Within each search of this database, hits represent websites that can be searched using National Archives. Each database was searched using "training" or "guidance"; no hits were returned in any searches.

Database: WHO
Date: 11/08/20

ID	Search Term(s)	Hits
1	Telehealth AND training with date limiter: 2015 onwards	118
2	Telemedicine AND training with date limiter: 2015 onwards	353
3	Telemedicine AND guidance with date limiter: 2015 onwards	294
4	Telehealth AND guidance with date limiter: 2015 onwards	111
5	mHealth AND training with date limiter: 2015 onwards	384
6	mHealth AND guidance with date limiter: 2015 onwards	344

Database: NHS England
Date: 11/08/20

ID	Search Term(s)	Hits
1	Telehealth with date limiter: 2015 onwards	19

For peer review only

2	Telemedicine with date limiter: 2015 onwards	19
3	ehealth with date limiter: 2015 onwards	18
4	mhealth with date limiter: 2015 onwards	22

Database: Charted Society of Physiotherapy
Date: 19/08/20

ID	Search Term(s)	Hits
1	Tele*	12
2	mHealth	4
3	eHealth	23
4	Tele* AND training	3538
5	Tele* AND guidance	2849

For peer review only

Procedural Guidance for Care Delivery – Derived from Recommendations Analysis

While the information below has been derived from articles that explored movement impairment, none of the recommended actions were specific for movement impairment interventions.

Procedural Guidance	Description
Pre-Delivery<br>▪ Environment Considerations<br>▪ Session Planning<br>▪ Technology Considerations<br>▪ Preparations with Client	In preparation for a telerehabilitation session, including actions such as planning the session environment, preparing the required technology, contacting the client, etc.
During Delivery<br>▪ Start<br>▪ Throughout<br>▪ End	During a telerehabilitation session, including actions such as introducing the agenda for that session, noting technical and safety concerns, summarising the session, etc.
Post-Delivery	After a telerehabilitation session, including actions such as documenting key messages of the session, deciding whether the patient needs further/additional support, and taking breaks when needed.

Pre-Delivery

Thirteen articles[1-13] suggested actions to take before engaging in a telerehabilitation session. These actions can be divided into Preparations with the Client, Environment Considerations, Session Planning, and Technology Considerations.

- Preparations with the Client: Contact the client to assess whether they are clinically appropriate to engage in telerehabilitation (e.g. whether face-to-face or urgent care is needed instead). If telerehabilitation is deemed appropriate, ensure the client has all necessary equipment (including suitable data/WIFI accessibility) and, if required, test any equipment with the client. Provide any information the client needs to attend the telerehabilitation session (e.g. link to logon to session, passwords, times, private and well-lit room, or other resources.). Ensure the client's phone number is available in case of technological issues during the session. Consider the client's context (e.g. hard of hearing or anxious about using technology) and address the context as appropriate if possible.
- Environment Considerations: Ensure the location from which one provides the telerehabilitation is quiet, private, not cluttered and will not be disturbed. Curtains should be drawn to reduce any on-screen glare from the sun. Clients should ensure the location is well-lit without relying on the sun. Test any technology (especially video and audio) to check they are working properly. If conducting a group session, ensure the size of the class is appropriate for the activities planned.
- Session Planning: Evaluate the client's functional status prior to the session and develop a clear agenda. Consider whether a client would benefit from a group or one-to-one session. If planning a group session, ensure there is enough time to address everyone's technological issues (e.g. connectivity issues, audio issues, etc.). Consider how many other staff members you may need (e.g. an extra member for support during a group session).

For peer review only

- Technology Considerations: Ensure any technology used is appropriate for the agenda of that session. Ensure there are back-up devices that can be used (e.g. telephone) if the main technology fails. Professional and patient clients should only open the software needed for the session and all other software should be closed. Ensure internet-using technology is wired to or close to the WIFI router, and that all required equipment are close-by. Position any cameras so that the whole face is within direct view. Ensure that all providers know how to use the required technology (e.g. knowing how to record or take screen shots) prior to the session.

During Delivery

Seven articles[1, 5, 7-11] suggested actions that should occur during the telerehabilitation session, which can be divided into the Start of the session, During the session, and the End of the session.

- Start: Welcome the client and ensure audio and video are working correctly. Confirm the identity of everyone attending the session. If a trusted other (e.g. carer) is also attending, check that the client is comfortable with that trusted other. If possible, lock the meeting to prevent others from entering. During a group session, ensure everyone in the group introduces themselves (including all staff). Summarise the session's agenda and introduce the roles of any trusted other or staff (e.g. assisting with note taking). Ensure the client(s) know what to do if the session is cut. Ask the client if they have any questions or concerns before engaging in the telerehabilitation session. Ensure the client is still clinically appropriate to engage in telerehabilitation (i.e. they do not need face-to-face or urgent care). If taking screen shots or recording, ensure the client knows and you have their consent. Finally, ask the client to show their environment to ensure any rehabilitation activities can be conducted safely and accurately.
- During: Document activities as per your professional governing body requirements, yet also include any technical issues and safety concerns from the client. Be patient during technical glitches (e.g. blurred video or audio cutting out). Understand that communication is harder for the client and ensure communication is intentional (e.g. verbal cues to let the client know you are listening). Ensure that you encourage the client to engage (e.g. by asking questions). The professional should inform the client when and why they might be pre-occupied (e.g. taking notes or looking for a resource).
- End: Carefully and clearly summarise key messages from the session. Let the client know if another session is required and when that will take place. Similarly, let the client know the required details if they need to be referred to another service. Confirm and record that the client is comfortable in engaging in further telerehabilitation sessions. Ensure the client knows self-care activities and their regime. Ask the client if anything needs to be clarified. Provide the client contact details if they need to contact the provider again. When ending the call, let the client know explicitly and then end the call.

Post Delivery

Three articles[5, 10, 11] suggested actions that should occur after a telerehabilitation session. Re-evaluate the client's functional ability. Document key messages of the session as required. Consider if a follow-up call or other structural support is needed for the client. Ensure to take breaks between telerehabilitation sessions to reduce any fatigue and look after vocals.

For peer review only

References

1. AHPScot, *A guide to Musculoskeletal (MSK) Digital Health consultations during Coronavirus (COVID-19)*. 2020.
2. Anton, D., et al., *A Telerehabilitation System for the Selection, Evaluation and Remote Management of Therapies.* Sensors (Basel). **18**(5).
3. Block, V.A., et al., *Remote Physical Activity Monitoring in Neurological Disease: A Systematic Review.* PLoS One, 2016. **11**(4): p. e0154335.
4. Calvaresi, D., et al., *Real-time multi-agent systems for telerehabilitation scenarios.* Artif Intell Med. **96**: p. 217-231.
5. Health and Social Care Board, *AHP Virtual Consultation Guidance*. 2020.
6. Paneroni, M., et al., *Is Telerehabilitation a Safe and Viable Option for Patients with COPD? A Feasibility Study.* Copd. **12**(2): p. 217-25.
7. Charted Society of Physiotherapy, *COVID-19: guide for rapid implementation of remote consultations*. 2020.
8. Royal College of Occupational Therapists, *Virtual Assessments/Reviews: The Script.* 2020.
9. Royal College of Speech and Language Therapists, *MAINTAINING SECURITY FOR TELEHEALTH SESSIONS*. 2020.
10. East Lancashire Hospitals NHS Trust, *Telepractice dysphagia assessmentsGuidelines for use with a patient in their own home/nursing home*. 2020.
11. Middleton, A., et al., *COVID-19 Pandemic and Beyond: Considerations and Costs of Telehealth Exercise Programs for Older Adults With Functional Impairments Living at Home-Lessons Learned from a Pilot Case Study.* Phys Ther.
12. Charted Society of Physiotherapy, *Telephone guidance for musculoskeletal practice*. 2020.
13. Charted Society of Physiotherapy, *Digital tools to support service delivery*. 2020.

For peer review only

Preferred Reporting Items for Systematic reviews and Meta-Analyses extension for Scoping Reviews (PRISMA-ScR) Checklist

SECTION	ITEM	PRISMA-ScR CHECKLIST ITEM	REPORTED ON PAGE #
**TITLE**			
Title	1	Identify the report as a scoping review.	
**ABSTRACT**			
Structured summary	2	Provide a structured summary that includes (as applicable): background, objectives, eligibility criteria, sources of evidence, charting methods, results, and conclusions that relate to the review questions and objectives.	
**INTRODUCTION**			
Rationale	3	Describe the rationale for the review in the context of what is already known. Explain why the review questions/objectives lend themselves to a scoping review approach.	
Objectives	4	Provide an explicit statement of the questions and objectives being addressed with reference to their key elements (e.g., population or participants, concepts, and context) or other relevant key elements used to conceptualize the review questions and/or objectives.	
**METHODS**			
Protocol and registration	5	Indicate whether a review protocol exists; state if and where it can be accessed (e.g., a Web address); and if available, provide registration information, including the registration number.	
Eligibility criteria	6	Specify characteristics of the sources of evidence used as eligibility criteria (e.g., years considered, language, and publication status), and provide a rationale.	
Information sources*	7	Describe all information sources in the search (e.g., databases with dates of coverage and contact with authors to identify additional sources), as well as the date the most recent search was executed.	
Search	8	Present the full electronic search strategy for at least 1 database, including any limits used, such that it could be repeated.	
Selection of sources of evidence†	9	State the process for selecting sources of evidence (i.e., screening and eligibility) included in the scoping review.	
Data charting process‡	10	Describe the methods of charting data from the included sources of evidence (e.g., calibrated forms or forms that have been tested by the team before their use, and whether data charting was done independently or in duplicate) and any processes for obtaining and confirming data from investigators.	
Data items	11	List and define all variables for which data were sought and any assumptions and simplifications made.	
Critical appraisal of individual sources of evidence§	12	If done, provide a rationale for conducting a critical appraisal of included sources of evidence; describe the methods used and how this information was used in any data synthesis (if appropriate).	
Synthesis of results	13	Describe the methods of handling and summarizing the data that were charted.	

For peer review only

St. Michael's
Inspired Care.
Inspiring Science.

SECTION	ITEM	PRISMA-ScR CHECKLIST ITEM	REPORTED ON PAGE #
**RESULTS**			
Selection of sources of evidence	14	Give numbers of sources of evidence screened, assessed for eligibility, and included in the review, with reasons for exclusions at each stage, ideally using a flow diagram.	
Characteristics of sources of evidence	15	For each source of evidence, present characteristics for which data were charted and provide the citations.	
Critical appraisal within sources of evidence	16	If done, present data on critical appraisal of included sources of evidence (see item 12).	
Results of individual sources of evidence	17	For each included source of evidence, present the relevant data that were charted that relate to the review questions and objectives.	
Synthesis of results	18	Summarize and/or present the charting results as they relate to the review questions and objectives.	
**DISCUSSION**			
Summary of evidence	19	Summarize the main results (including an overview of concepts, themes, and types of evidence available), link to the review questions and objectives, and consider the relevance to key groups.	
Limitations	20	Discuss the limitations of the scoping review process.	
Conclusions	21	Provide a general interpretation of the results with respect to the review questions and objectives, as well as potential implications and/or next steps.	
**FUNDING**			
Funding	22	Describe sources of funding for the included sources of evidence, as well as sources of funding for the scoping review. Describe the role of the funders of the scoping review.	

JBI = Joanna Briggs Institute; PRISMA-ScR = Preferred Reporting Items for Systematic reviews and Meta-Analyses extension for Scoping Reviews.

* Where *sources of evidence* (see second footnote) are compiled from, such as bibliographic databases, social media platforms, and Web sites.

† A more inclusive/heterogeneous term used to account for the different types of evidence or data sources (e.g., quantitative and/or qualitative research, expert opinion, and policy documents) that may be eligible in a scoping review as opposed to only studies. This is not to be confused with *information sources* (see first footnote).

‡ The frameworks by Arksey and O'Malley (6) and Levac and colleagues (7) and the JBI guidance (4, 5) refer to the process of data extraction in a scoping review as data charting.

§ The process of systematically examining research evidence to assess its validity, results, and relevance before using it to inform a decision. This term is used for items 12 and 19 instead of "risk of bias" (which is more applicable to systematic reviews of interventions) to include and acknowledge the various sources of evidence that may be used in a scoping review (e.g., quantitative and/or qualitative research, expert opinion, and policy document).

From: Tricco AC, Lillie E, Zarin W, O'Brien KK, Colquhoun H, Levac D, et al. PRISMA Extension for Scoping Reviews (PRISMAScR): Checklist and Explanation. Ann Intern Med. 2018;169:467–473. doi: 10.7326/M18-0850.

For peer review only - http://bmjopen.bmj.com/site/about/guidelines.xhtml

BMJ Open

The scope, context, and quality of telerehabilitation guidelines for physical disabilities: a scoping review

Journal:	*BMJ Open*
Manuscript ID	bmjopen-2021-049603.R1
Article Type:	Original research
Date Submitted by the Author:	19-May-2021

Complete List of Authors:	Anil, Krithika; University of Plymouth, School of Health Professions<br>Freeman, Jennifer; Peninsula Allied Hlth Ctr, School of Health Professions<br>Buckingham, Sarah; University of Plymouth, School of Health Professions<br>Demain, Sara; Plymouth University, School of Health Professions<br>Gunn, H; University of Plymouth, School of Health Professions<br>Jones, Ray; Plymouth University, School of Nursing and Midwifery<br>Logan, Angela; Royal Devon and Exeter NHS Foundation Trust<br>Marsden, Jonathan; Plymouth University, School of Health Professions<br>Playford , Diane ; University of Warwick, Warwick Medical School<br>Sein, Kim; University of Plymouth, School of Health Professions<br>Kent, Bridie; Plymouth University, Nursing and Midwifery, Innovations in Health and Social Care: A JBI Centre of Excellence

**Primary Subject Heading**:	Health services research
Secondary Subject Heading:	Health services research, Rehabilitation medicine
Keywords:	COVID-19, Telemedicine < BIOTECHNOLOGY & BIOINFORMATICS, Physiology < NATURAL SCIENCE DISCIPLINES, Protocols & guidelines < HEALTH SERVICES ADMINISTRATION & MANAGEMENT, REHABILITATION MEDICINE

SCHOLARONE™ Manuscripts

I, the Submitting Author has the right to grant and does grant on behalf of all authors of the Work (as defined in the below author licence), an exclusive licence and/or a non-exclusive licence for contributions from authors who are: i) UK Crown employees; ii) where BMJ has agreed a CC-BY licence shall apply, and/or iii) in accordance with the terms applicable for US Federal Government officers or employees acting as part of their official duties; on a worldwide, perpetual, irrevocable, royalty-free basis to BMJ Publishing Group Ltd ("BMJ") its licensees and where the relevant Journal is co-owned by BMJ to the co-owners of the Journal, to publish the Work in this journal and any other BMJ products and to exploit all rights, as set out in our licence.

The Submitting Author accepts and understands that any supply made under these terms is made by BMJ to the Submitting Author unless you are acting as an employee on behalf of your employer or a postgraduate student of an affiliated institution which is paying any applicable article publishing charge ("APC") for Open Access articles. Where the Submitting Author wishes to make the Work available on an Open Access basis (and intends to pay the relevant APC), the terms of reuse of such Open Access shall be governed by a Creative Commons licence – details of these licences and which Creative Commons licence will apply to this Work are set out in our licence referred to above.

Other than as permitted in any relevant BMJ Author's Self Archiving Policies, I confirm this Work has not been accepted for publication elsewhere, is not being considered for publication elsewhere and does not duplicate material already published. I confirm all authors consent to publication of this Work and authorise the granting of this licence.

For peer review only

Title Page

Manuscript Title: The scope, context, and quality of telerehabilitation guidelines for physical disabilities: a scoping review

Word Count: 3914

Authors: Krithika Anil[1] (0000-0002-8027-1665), Jennifer A Freeman[2], Sarah Buckingham[3], Sara Demain[4], Hilary Gunn[5], Ray B Jones[6], Angela Logan[7], Jonathan Marsden[8], E Diane Playford[9], Kim Sein[10], Bridie Kent[11,12]

[1] Research Fellow; Peninsula Allied Health Centre, School of Health Professions, University of Plymouth, Derriford Road, Plymouth, PL6 8BH, United Kingdom; krithika.anil@plymouth.ac.uk

[2] Professor of Physiotherapy and Rehabilitation, Peninsula Allied Health Centre, School of Health Professions, University of Plymouth, Derriford Road, Plymouth, PL6 8BH, United Kingdom; jenny.freeman@plymouth.ac.uk; +44 1752 588835

[3] Research Fellow; Peninsula Allied Health Centre, School of Health Professions, University of Plymouth, Derriford Road, Plymouth, PL6 8BH, United Kingdom; sarah.buckingham@plymouth.ac.uk

[4] Head of School of Health Professions, Peninsula Allied Health Centre, School of Health Professions, University of Plymouth, Derriford Road, Plymouth, PL6 8BH, United Kingdom; sara.demain@plymouth.ac.uk; +44 1752 587570

[5] Associate Professor in Physiotherapy, Peninsula Allied Health Centre, School of Health Professions, University of Plymouth, Derriford Road, Plymouth, PL6 8BH, United Kingdom; hilary.gunn100@plymouth.ac.uk; +44 1752 588825

[6] Professor of Health Informatics, School of Nursing and Midwifery, University of Plymouth, Drake Circus, Plymouth, PL4 8AA, United Kingdom; ray.jones@plymouth.ac.uk; +44 1752 586532

[7] Consultant Physiotherapist, Stroke Rehabilitation, Royal Devon and Exeter NHS Foundation Trust, Barrack Road, Exeter, Devon, EX2 5DW, United Kingdom; angie.logan@plymouth.ac.uk; +44 1752 587575

[8] Professor and Chair in Rehabilitation, Peninsula Allied Health Centre, School of Health Professions, University of Plymouth, Derriford Road, Plymouth, PL6 8BH, United Kingdom; jonathan.marsden@plymouth.ac.uk; +44 1752 587590

[9] Honorary Professor of Neurological Rehabilitation, Warwick Medical School, Gibbet Hill Campus, University of Warwick, CV4 7AL, United Kingdom; d.playford@warwick.ac.uk

[10] Research Fellow; Peninsula Allied Health Centre, School of Health Professions, University of Plymouth, Derriford Road, Plymouth, PL6 8BH, United Kingdom; kim.sein@plymouth.ac.uk

[11] Professor of Leadership in Nursing, School of Nursing and Midwifery, University of Plymouth, Drake Circus, Plymouth PL4 8AA, United Kingdom; bridie.kent@plymouth.ac.uk; +44 1752 588817

For peer review only

[12] Professor of Leadership in Nursing, Director of the University of Plymouth Centre for Innovations in Health and Social Care: A JBI Centre of Excellence, University of Plymouth, Drake Circus, Plymouth PL4 8AA, United Kingdom; bridie.kent@plymouth.ac.uk; +44 1752 588817

Corresponding author: Krithika Anil
Contact email: krithika.anil@plymouth.ac.uk

ABSTRACT

Objective To identify the available guidance and training to implement telerehabilitation movement assessments for people (adults and children) with a physical disability, including those recovering from COVID-19

Design Rapid scoping review

Included Sources and Articles PubMed, CINAHL, PsychInfo, Cochrane, EMBASE, Web of Science, PEDro, UK Health Forum, WHO, National Archives, and NHS England, searched using the Participant-Concept-Context (PCC) framework from 2015 to August 2020. Primary studies that recruited individuals with physical disabilities and guidance documents aimed at providers to implement movement-related telerehabilitation were included.

Results 23 articles (11 primary research studies, 3 systematic reviews and 9 guidance documents) were included out of 7857 that were identified from the literature search. Two main issues were found: (1) telerehabilitation guidance (from both research studies and guidance documents) was not specific to movement-related assessment, and (1) most primary research studies provided neither guidance nor training of movement-specific assessment to practitioners. Of the COVID-19-related guidance, two articles reported COVID-19 management that only referred to identifying COVID-19 status without references to specific movement-related guidance.

Conclusions Telerehabilitation guidance and training have existed pre-COVID-19, yet the lack of specific movement-related information and provider support is surprising. This gap must be addressed to optimise effective implementation of remote assessments for those with physical disabilities.

Review Registration Open Science Framework: osf.io/vm6sp

Key words: Telerehabilitation, physical disability, remote consultation, COVID-19, scoping review, guidance

STRENGTHS AND LIMITATIONS

- A wide variety of articles were reviewed due to extensive searches for grey literature and no language restrictions
- Despite no language restrictions, included articles were biased towards the English language
- Some articles within this review may have been updated since the initial search and others have likely emerged since

For peer review only - http://bmjopen.bmj.com/site/about/guidelines.xhtml

INTRODUCTION

Since the outbreak of the COVID-19 pandemic, health services across the world have rapidly adapted to a new way of working. They have embraced the need to use a "digital first" approach to maintain delivery of care as best as possible, whilst protecting both staff and patients[1]. In the field of rehabilitation this approach is typically referred as "telerehabilitation", defined as: The delivery of rehabilitation services via information and communication technologies[2]. This includes a broad range of services from assessment and monitoring to education and consultation. There is a general acknowledgement that, beyond the pandemic, remote delivery of services will be an integral part of everyday practice within future health and social care systems. Ensuring that the delivery of such services is both effective and equitable is pertinent, given the identified "tsunami" of rehabilitation need[3 4] for people with a wide range of conditions from across the life-span (including those recovering from COVID-19[5]). It is especially important to note that the practical application of telerehabilitation will not be the same across all conditions. More complicated conditions (e.g. those with co-morbidity) will likely require additional support than less complicated conditions. This additional consideration further demonstrates the need for comprehensive training and guidance for telerehabilitation.

Key to targeting an effective and personalized rehabilitation plan is the need for a comprehensive, detailed, and valid assessment[6], with ongoing monitoring and evaluation as needs change. When the primary concerns relate to physical disability, an assessment of aspects such as dynamic posture, balance and movement is fundamental. This therefore raises important questions related to how these assessments can be undertake remotely, whilst ensuring they are both safe and effective. For instance, is it possible to adequately and safely assess the balance of an individual who is falling/at risk of falls? Or the source of shoulder pain? Or the relative impact of spasticity and weakness on movement? Do the additional practical challenges such as slow internet speeds and static and restricted camera angles mean that an assessment of dynamic movement is too restricted to inform management plans? Are the clinical skills required for this type of remote assessment different to those utilised for face-to-face assessment? And if so, is there specific guidance and training related to these aspects of remotely based physical assessments to assist practitioners with implementing this effectively and efficiently? It was our observation that questions such as these have been raised by rehabilitation organisations, professional networks and individual practitioners and require urgent answers, hence the need for this rapid scoping review.

This scoping review formed part of an overall research project aimed at developing and evaluating a training package to support the remote assessment and management of people with physical disability (UK RI-NIHR, MRC, Covid-19: MR/V021060/1). It was not appropriate to involve patients or the public in the design, or conduct, or reporting of this research.

Review question and objectives

What guidance and training is available to implement telerehabilitation movement assessment for people (children and adults) with a physical disability, including those recovering from COVID-19? The review objectives were to identify:

- The contexts and types of telerehabilitation assessments delivered in a health and social care setting for people with a physical disability (including those recovering from COVID-19)
- Any specific guidance and/or training available for delivering telerehabilitation movement-related assessments
- Specific recommendations regarding movement-related assessments for effective implementation of telerehabilitation guidance

For peer review only - http://bmjopen.bmj.com/site/about/guidelines.xhtml

METHODS

Type of Review

This scoping review used the JBI approach[7]. This approach seeks to explore the breadth or extent of the literature, map and summarise the evidence, and inform future research[8]. The PRISMA checklist for scoping reviews was used for the review (see supplementary material titled "SM – PRISMA Statement for Scoping Reviews"). There is no registration database specific for scoping reviews, thus the review was uploaded to Open Science Framework: osf.io/vm6sp.p9

Patient and Public Involvement

No patients involved.

Inclusion Criteria

The inclusion criteria were developed using the PCC (participant-concept-context) framework[9].

Participants

The review considered articles that included individuals of all ages with a physical disability. Physical disability was defined as any physical condition that affects a person's mobility, physical capacity, stamina, or dexterity. The review included studies of participants with a physical disability who had any duration and severity of physical disability. There was no age restriction. Guidance and policy documents aimed at undertaking telerehabilitation with individuals with physical disabilities were also included.

Concept

Studies and policies that examined or described guidance and training regarding implementation of telerehabilitation movement assessments were considered. *Guidance* included checklists, tools, outcome measurement batteries, protocols, guidelines, risk assessments, exemplars, and appraisals. *Training* included interactions, communications, and resources with the aim to improve and/or inform the process of delivering telerehabilitation. Guidance and training must have involved movement evaluation; that is, the evaluation of how people move around, perform, and function. This included self-administered and practitioner administered telerehabilitation. Studies and policies that did not involve movement evaluation were excluded.

Context

This review considered studies that provided guidance and training in health and social care contexts, that is, telerehabilitation must have been provided by a practitioner, system, or institute.

Types of Literature

The following study designs were included: randomised controlled trials (RCTs), non-RCTs, quasi-experimental, pre- and post- studies, case studies, observational studies, systematic reviews, and qualitative studies. Grey literature regarding policies from recognised UK-based national and international institutes were also considered. Opinion pieces, non-systematic literature reviews, and other grey literature were excluded. No language restrictions were implemented.

Date Restriction

Publications from 2015 to Aug 2020 were included in this review because technologies and related-guidance older than this period were considered out-of-date due to the rapid speed of technology development in areas such as robotics, artificial intelligence, and telepresence[10 11].

For peer review only - http://bmjopen.bmj.com/site/about/guidelines.xhtml

For peer review only

Search Strategy

An initial limited search of PubMed and CINAHL was undertaken to estimate the volume of relevant literature and to identify key words to assist in developing search terms. A second search using the developed search terms was undertaken (supported by a review-specialist librarian) and adapted across each included information source (PubMed, CINAHL, PsychInfo, Cochrane, EMBASE, Web of Science, PEDro, UK Health Forum, WHO, National Archives, NHS England). Due to the rapid nature of this review, study authors were not contacted for further information in cases of ongoing or uncompleted studies. Please see supplementary materials (SM1 and SM2) for search strategy terms.

Study Selection

Following the search, all identified references were imported into Endnote[12] (a reference management software). After removal of duplications within EndNote, references were uploaded to the online Rayyan tool[13] (a review organisation tool). Titles and abstracts were screened for assessment against the inclusion criteria for the review. The full text of potentially eligible studies were retrieved and assessed in detail against the inclusion criteria. Full text studies that did not meet the inclusion criteria were excluded and are displayed in a PRISMA flowchart (see Figure 1). Any disagreements that arose between the reviewers were resolved through discussion. As this review is a rapid scoping review no quality or risk of bias assessment was undertaken.

Data Extraction

Data extraction of included studies was conducted by the review team. Data was charted using a customised Excel spreadsheet. The data extracted specific information pertaining to the review objectives and key information for each article were charted as per JBI Guidance[7]. This included telerehabilitation contexts, types of telerehabilitation, guidance and training, and any recommendations for implementing telerehabilitation. Any disagreements between reviewers were resolved through discussion.

Data Synthesis

The narrative synthesis of the findings from included studies, policies, and guidance documents was structured according to the review objectives. Findings were presented narratively, aided by appropriate tables and figures. Recommendations in the literature were analysed using descriptive qualitative analysis[14] to identify common themes. Only recommendations focusing on clinical implementation or technological improvements were analysed (recommendations for future research within the included studies were not analysed).

Mentions of the 'provider' refer to the individual providing the telerehabilitation, such as health or social care practitioners. Mentions of the 'client' refer to the individual receiving the telerehabilitation, i.e. the patient.

RESULTS

Twenty-three articles were included in the scoping review analysis (Figure 1; see Table 1 for article characteristics). Eleven[15-25] of the included articles were original research studies and three were systematic review studies[26-28]. The remaining nine[29-37] were guidance documents produced by health institutions or health experts. Guidance documents are highlighted in grey and systematic review articles are highlighted in yellow in Table 1. Eleven articles were specified for adults[15-19 21-24 26 27] (≥18 years), one article was specified for children[20] (≤18 years), and 11 articles did not specify age[25 28-37].

[Insert Figure 1 (Legend: Figure 1 PRISMA flow diagram that charts the study identification process)]

For peer review only - http://bmjopen.bmj.com/site/about/guidelines.xhtml

Table 1 Characteristics of the included articles

Author(s)	Date	Aim	Country Origin	Design	Total N	Physical Condition
Al Rajeh et al[15]	2019	Explore the use of telehealth and assess the perceptions of clinicians employing telehealth and specifically alarm settings on technology	UK	Cross-sectional	65	Chronic obstructive pulmonary disease
Amatya et al[26]	2015	Investigate the effectiveness and safety of telerehabilitation	Europe and USA	Systematic review	564	Multiple sclerosis
Anton et al[17]	2018	Develop and examine the Kinext Telerehabilitation System for physical conditions	Spain and Australia	Feasibility	18	Shoulder disorder; Hip replacement
Block et al[27]	2016	Perform a systematic review of studies using remote physical activity monitoring in neurological diseases	USA	Systematic review	943	Neurological diseases
Calvaresi et al[18]	2019	Examine the feasibility of using Artificial Intelligence for telerehabilitation	Italy	Mixed-methods	10	General physical conditions
Giesbrecht & Miller[22]	2019	Evaluate the effect of a mHealth wheelchair skills training program on clinical outcomes among older adult manual wheelchair clients	Canada	RCT	18	Physical disability needing wheelchair
Grau-Pellicer et al[23]	2020	Investigate the effectiveness of a mHealth app	Spain	RCT	41	Ischaemic or haemorrhagic stroke
Guerra de Oliveira Gondim et al[24]	2017	Evaluate the effects of individualized orientation and monitoring by telephone in a self-supervised home therapeutic exercise program on signs and symptoms of Parkinson’s disease and quality of life	Brazil	RCT	28	Parkinson's disease

For peer review only - http://bmjopen.bmj.com/site/about/guidelines.xhtml

Hasenohrl et al[19]	2020	Assess the feasibility and acceptance of orthopaedists prescribing individualised therapeutic exercises via a smartphone app to patients	Austria	Qualitative	27	Back pain
Levac et al[20]	2018	Develop and conduct a usability evaluation of the Fun, Interactive Therapy Board (FITBoard), a movement toy for children with disabilities	USA	Observational	7	General movement conditions
Teleswallowing Ltd[31]	2020	Provide evidence-based guidance on designing and establishing telerehabilitation services for dysphagia	UK	Guidance	N/A	Dysphagia
Mani et al[28]	2017	Systematically explore and summarise the validity and reliability of a telerehabilitation -based physiotherapy assessment	Malaysia	Systematic review	NS	MSK conditions
Middleton et al[16]	2020	Describe the process and cost of delivering a synchronous telehealth exercise programme	USA	Observational	1	Stroke, hypertension, and diabetes
Paneroni et al[21]	2015	Test the feasibility, adherence and satisfaction of a home-based reinforcement telerehabilitation programme	Italy	Observational	36	Chronic obstructive pulmonary disease
Charted Society of Physiotherapy[33]	2020	Provide guidance for physiotherapists on digital tools to support service delivery	UK	Guidance	N/A	General physical conditions
Charted Society of Physiotherapy[32]	2020	Provide guidance for physiotherapists on telephone triage of patients with musculoskeletal conditions	UK	Guidance	N/A	MSK conditions

For peer review only - http://bmjopen.bmj.com/site/about/guidelines.xhtml

Charted Society of Physiotherapy[34]	2020	Provide practical advice for physiotherapists and support workers to implement remote consultations	UK	Guidance	N/A	General physical conditions
Royal College of Speech and Language Therapists[36]	2020	Provide brief tips for telerehabilitation implementation revolved around security	UK	Guidance	N/A	General physical conditions
East Lancashire Hospitals NHS Trust[37]	2020	Provide brief tips for telerehabilitation implementation for dysphagia	UK	Guidance	N/A	Dysphagia
Venkataraman et al[25]	2016	Compare Berg Balance Scale ratings using videos with differing transmission characteristics with direct in-person rating	USA	Psychometrics study	45	General motor control condition
Health and Social Care Board NI[30]	2020	Amalgamate evidence and guidance on the conduct of virtual consultations so that it is easily accessible for AHPs	UK	Guidance	N/A	General physical conditions
AHPScot[29]	2020	Guide AHPs in NHS Scotland during remote consultations (telephone or video) with MSK patients during the current COVID19 pandemic	UK	Guidance	N/A	MSK conditions
Royal College of Occupational Therapists[35]	2020	Guide occupational therapists regarding implementation of virtual assessments and reviews	UK	Guidance	N/A	General physical conditions

Grey highlighted rows indicate a guidance document
N/A = Not applicable
NS = Not specified
AHPs = Allied health professionals
MSK = Musculoskeletal
UK = United Kingdom
USA = United States of America

For peer review only - http://bmjopen.bmj.com/site/about/guidelines.xhtml

The 14 included research studies and systematic reviews had the following general aims: one study assessed use and perceptions of telerehabilitation[15]; one study examined costs of implementing telerehabilitation[16]; one study assessed the development of a telerehabilitation technology[17]; three studies systematically reviewed telerehabilitation literature[26-28]; four studies assessed the feasibility of a telerehabilitation technology[18-21]; and four studies examined the effectiveness of the telerehabilitation technology[22-25].

Contexts

Client setting was reported in 13 of the 23 articles, where only one article did not include a home setting and instead only used general clinical sites[20] (Table 2). The client support environment (i.e. who was with the client) was reported in 13 articles (Table 2), where most clients were alone (n=5). Only one article specifically reported that carers were present during the consultation or intervention. Table 3 shows the wide range of providers using telerehabilitation in people with physical disability, most being a physiotherapist. Of note, all non-qualified assistants were accompanied by a qualified health professional throughout the telerehabilitation sessions. This article[20] described the development and preliminary usability, from the provider's perspective, of a tablet-based interactive movement tool. Provider setting was reported in six articles and all were telerehabilitation technologies used by providers in secondary care (e.g. an out-patient clinic;[17 19 21-23 25]).

For peer review only - http://bmjopen.bmj.com/site/about/guidelines.xhtml

Table 2 Cross-tabulation of Client Support and Client Setting information extracted from included articles

			**Client Support Environment**			
		Article Type	*Not specified*	*Client with group*	*Client alone*	*Client with carer*
**Client Setting**	*Not specified*	Primary Research	*2[15 17]*	-	*1[25]*	-
		Reviews	-	**1[28]*	**1[28]*	-
		Guidance Documents	*6[31 32 34-37]*	-	-	-
	At home	Primary Research	-	-	*4[16 19 21 22]*	*1[24]*
		Reviews	-	-	-	-
		Guidance Documents	-	-	-	-
	Home and community	Primary Research	*1[18]*	-	-	-
		Reviews	-	**2[26 27]*	**2[26 27]*	-
		Guidance Documents	*1[33]*	**2[29 30]*	**2[29 30]*	-
	General clinic sites	Primary Research	*1[20]*	-	-	-
		Reviews	-	-	-	-
		Guidance Documents	-	-	-	-
	Rehabilitation unit and at home	Primary Research	-	*1[23]*	-	-
		Reviews	-	-	-	-
		Guidance Documents	-	-	-	-

*Five articles (3 reviews[26-28] and 2 guidance documents[29 30]) had participants complete the telerehabilitation alone and in a group - these articles have been added to both the "Client was Alone" and "Client with Group" categories.

For peer review only - http://bmjopen.bmj.com/site/about/guidelines.xhtml

Table 3 List of telerehabilitation administrators extracted from included articles

Telerehabilitation Administrator*	N[a]	N[b]	N[c]
Physiotherapist	9[15-18 20 21 23-25]	1[28]	1[34]
Occupational therapist	3[20 22 25]	-	1[35]
Medic	3[15 19 21]	1[27]	-
Nurse	2[15 21]	-	-
Speech and language therapist	-	-	2[31 37]
General clinicians	-	-	2[32 33]
AHPs	-	-	2[29 30]
Not specified	1[36]	1[26]	-
Assistants	2[16 25]	-	-
Physiologist	1[15]	-	-

AHP = Allied Health Professional, specific profession not stated
*Telerehabilitation administrators overlap in articles, therefore total N does not sum to total included articles
[a]N for primary research studies
[b]N for systematic review studies
[c]N for guidance documents

Presence of COVID-19

Only articles published in 2020[16 19 23 29-37] (n = 12) were considered for COVID-19-specific information. Of these, two articles refer to COVID-19-related management; guidance documents developed by AHPScot [29] and the Health and Social Care Board Northern Ireland[30]. The COVID-19-related recommendations from these guides are detailed in the Recommendations section. It is noteworthy that these guidance documents refer to COVID-19 in general and are not specifically related to telerehabilitation use in people with movement impairment, despite these documents generally considering the use of telerehabilitation for this purpose. Teleswallowing Ltd.[31], Middleton et al[16], and two articles from the Charted Society of Physiotherapy[33 34] also refer to COVID-19 but only state that telerehabilitation is useful to decrease the spread of COVID-19; these articles do not refer to management of patients with COVID-19.

Types of Telerehabilitation

Table 4 shows the aims of the telerehabilitation interventions examined in the primary research studies. Nine guides[29-37], 3 systematic reviews[26-28], and 2 primary studies[18 25] are excluded from Table 4 as they did not evaluate a specific telerehabilitation technology. Most (5 of the 9 studies) telerehabilitation interventions aimed to assist in management of a movement condition; however, some cited additional aims such as optimising adherence[15], improving quality of life[24], monitoring, and assessing client satisfaction[23]. One intervention provided training in using a wheelchair[22].

We aimed to summarise the various platforms used for delivery of telerehabilitation but this proved difficult, as some articles did not have sufficient detail. For example, one article[16] reports a tablet app but does not specify whether video was involved or whether it just used a chat/telephone function. Accepting these limitations, the most common delivery system appeared to be via video (e.g. video call, Skype, video-recording, etc.), followed by apps (on tablets or mobiles) and telephone calls. Fifteen of the telerehabilitation platforms were synchronous[16 17 19 20 22-24 28-31 33 35-37] (i.e. in real-time) and seven

For peer review only - http://bmjopen.bmj.com/site/about/guidelines.xhtml

platforms used both synchronous and asynchronous[15 18 21 26 27 32 34] (i.e. not in real-time) methods. Only one article used only an asynchronous platform where providers assessed physical ability from a video-recording of the client[25].

Table 4 List of telerehabilitation aims and platforms reported in the included articles

Telerehabilitation Aim	N	References
MCM	5	16 17 19-21
MCM and adherence	1	15
MCM and quality of life	1	24
MCM and client satisfaction	1	23
Training for assistive device use	1	22

MCM = Movement condition management

Of the 11 primary research studies, five[15 17-19 22] specified neither number of sessions nor session duration, one specified just number and one specified just duration. The number of sessions ranged from 1 to 36 (n = 2 for 1 session[20 25]; n = 1 for 16 sessions[23]; n = 1 for 28 sessions[21]; n = 2 for 36 sessions[16 24]). Three studies included 60 minute sessions[20 23 24], one included 50 minute sessions[16], and one included 100 minute sessions[21]. The variability seen here is reflective of that seen in face-to-face rehabilitation sessions.

Guidance

Specific guidance on how to use telerehabilitation technology was supplied to providers in 12 articles[20 22 25 29-37], of which three were studies[20 22 25] and the remaining were guidance documents[29-37]. A guidebook or manual was provided in ten articles[20 22 25 29-31 33-35 37], while two provided a checklist[32 36]. No guidance was supplied in seven articles[15 16 19 21 23 24], and five studies did not mention whether guidance was provided or not[17 18 26-28].

Training

Guidance documents and systematic reviews were not included in training analysis as these articles had no participants to whom to supply training. Specific training on how to use telerehabilitation technology was supplied to providers in 4 out of 11 primary research studies[16 20 22 25]. The training was provided in-person in three of these studies [16 20 22], while the remaining study provided training via a video tutorial[25]. No training was supplied in five studies[15 19 21 23 24], and 2 studies did not mention training[17 18].

Recommendations

Six themes and 11 subthemes were identified within the recommendations: Administrative Advice, Challenges, Governance, Social Support, Technology Improvements, and COVID-19 (see Table 5). Recommendations were also analysed to extract procedural guidance that providers can perform in preparation for, during, and after a telerehabilitation session. This guidance is summarised in supplementary materials (SM3) as it extended the review beyond its key message.

For peer review only - http://bmjopen.bmj.com/site/about/guidelines.xhtml

Table 5 Summary of themes identified during qualitative analysis of article recommendations

Analysis Themes	Description
Administrative Recommendations<br>▪ General<br>▪ Client-Specific<br>▪ Technology-Specific<br>▪ Data Security	Advice revolved around administrative duties, such as record keeping, technology maintenance, or data security
Challenges<br>▪ Care Provision<br>▪ Social Factors<br>▪ Technology Issues	Challenges that providers may encounter when conducting telerehabilitation services
Governance	Ethical advice, such as client consent and/or assessing confidentiality
Support Mechanisms<br>▪ Providers<br>▪ Carers<br>▪ Provider Assistants<br>▪ Non-Clinical Staff	Advice that emphasised the important role of support from various sources involved in providing telerehabilitation care to the client
Technology Improvements	Suggestions that may improve the use and function of a telerehabilitation technology
COVID-19	Actions providers are advised to conduct to address COVID-19 risks

Administrative Recommendations
Three articles[30 32 36] provided advice on administrative duties that providers should perform when conducting telerehabilitation. The advice revolved around four sub-themes:
(1) General Advice involved general administrative duties such as maintaining record-keeping standards and identifying the specific resources a provider will need when engaging in telerehabilitation (e.g. number of sessions needed, appropriate length of session considering technology set-up, clinical outcome measures needed, etc.).
(2) Client-Specific Advice involved duties that required interactions with the client, such as documenting all client conversations and noting care that would have been provided if the intervention was delivered face to face but was not done so due to the restrictions of telerehabilitation.
(3) Technology-Specific Advice involved ensuring technology was up-to-date and that the latest version of any software was being used.
(4) Data Security emphasised that providers must minimise identifiable information that is shared digitally, ensure that all actions comply with information governance, and only use technology recognised by the institute through which they provide their care services.

Challenges
Six articles[15 19 23 25 28 30] highlighted challenges when conducting telerehabilitation, which involved three sub-themes:
(1) Care Provision considers the resources needed for telerehabilitation, highlighting that some clinical assessments may be too complicated to conduct effectively at a distance and that, if this complication is not recognised by inexperienced telerehabilitation providers, the quality of care provided may be reduced.

For peer review only - http://bmjopen.bmj.com/site/about/guidelines.xhtml

(2) Social Factors identified social challenges, where unsatisfactory video etiquette, minimal rapport-building, and socio-economic disadvantages have the potential to reduce the quality of care provided via telerehabilitation.
(3) Technology Issues relate to technology failures or interruptions, such as inadequate internet connection, low camera/audio quality, and false alarms (on wearable devices) that result in a reduced quality of care.

Governance
Six articles[29 30 34-37] provided recommendations relating to governance issues. The guidance highlighted that all clients must be informed about the following: telerehabilitation is voluntary; potential risks when engaging in telerehabilitation; what will happen with their data; and what reasonable actions they should take to ensure the security of their technology. This theme also emphasised the importance of consent, where informed consent must be taken explicitly from clients when video/audio recording or taking screen shots. If required, clients must be asked whether they consent for their trusted others (e.g. carer or family) to attend the session with them. However, one article [30] stated that consent was implied when clients enter the telerehabilitation session.

Support Mechanisms
Six articles[21 23 24 28 31 37] recommended support mechanisms to be set in place for clients when providing telerehabilitation. This support involved four sub-themes:
(1) Providers to offer support beyond the main goals of telerehabilitation, such as reassurance to clients who are anxious using technology for their rehabilitation.
(2) Carers to offer clients in-person support during delivery of telerehabilitation sessions. For instance, provision of physical assistance to safely enable the client to achieve correct posture or movement, or to assist the client with using the technology, such as positioning of the camera.
(3) Provider Assistants to assist qualified staff by visiting clients in person to assist telerehabilitation, such as undertaking outcome measures face-to-face, when required and safe to do so.
(4) Non-Clinical Staff, such as administrators and senior management, to provide essential support in successful coordination and implementation of telerehabilitation, emphasising the importance of effective communication between support staff and providers.

Technology Improvements
Eight articles[15 17-20 26-28] recommended technology improvements for telerehabilitation purposes. For instance, communications should be synchronous and technology must have a client-friendly interface that satisfies both the client and the provider. Wearable technologies relevant to posture and movement (e.g. to measure steps or heart rate) must be precise. Technology would especially benefit from improved monitoring capabilities, such as real-time movement tracking and activity adherence monitoring. If technology provided smart data (e.g. real-time measures of muscle strength), it should be accompanied by useful and actionable information. Reminders to engage in self-care activities would be useful for clients during their rehabilitation period. A built-in anatomical map to assist clients in showing the location of pain, tenderness, or other sensations was also suggested.

COVID-19
Two articles (both guidance documents[29 30]) made recommendations to address COVID-19 concerns. When establishing whether the client needs telerehabilitation or face-to-face care, professionals were advised to identify the client's COVID-19 status. Suggested identifications included: tested positive and self-isolating, has symptoms and self-isolating, has come into contact with someone who tested positive and is now self-isolating, shielding, or no current cautions outside of government restrictions. Of note, this is an area of rapid expansion in the literature.

For peer review only - http://bmjopen.bmj.com/site/about/guidelines.xhtml

DISCUSSION

The 23 articles included in this rapid scoping review revealed two main gaps in the literature: (1) telerehabilitation guidance (from research studies and guidance documents) is not specific to movement-related rehabilitation despite articles stating this explicitly as their aim, and (2) neither guidance nor training were given to providers in most research studies evaluating a telerehabilitation technology.

The Lack of Specific Guidance

The vast majority of the existing recommendations relate predominately to communication or governance elements of telerehabilitation, and lack specific guidance about issues pertinent to movement impairment. Several elements that are key to effectively implementing telerehabilitation for people with physical disability are missing. When assessing movement remotely, one would expect guidance on ensuring patient safety, triaging patients, and specific steps to decide whether a patient needs face-to-face care. More importantly, the identified guidance does not effectively address the limitations of undertaking physical assessments remotely; for example, if the patient is unable to move their body themselves if their movement is inadequate for assessment. Remote physical examinations are complex and need specific guidance on implementation; this is reflected in recent research that highlights physical examinations as one of the main challenges for remote assessments[38 39]. There was also minimal information on telerehabilitation related to COVID-19. This is unsurprising given the novelty of this infection. Overall, current telerehabilitation guidance for physical disabilities focuses on general tele-health guidance rather than being specific to movement impairments. Although issues such as data security, video etiquette, and organisation are important, they are not enough to assist providers in effective telerehabilitation implementation for people with movement impairments.

The Minimal Focus on Providers

Of the 11 included primary studies and 3 systematic reviews, only three[20 22 25] provided guidance to providers. Similarly, only four research studies[16 20 22 25] described the provision of training to providers. Neglecting providers' skills in using technology effectively (i.e. digital literacy) potentially undermines the usefulness of any telerehabilitation technology that was evaluated[40 41]. Furthermore, studies mainly report the clients' and not the providers' context of using telerehabilitation (e.g. whether the provider was from primary, secondary, or tertiary care). The generalisability of the telerehabilitation technology examined in the study is thus unclear, as certain technologies may only be effective in particular contexts[42]. Future studies should carefully consider providers' digital literacy and context to better understand the effectiveness of telerehabilitation technology. Digital literacy and context are not the only important factors for telerehabilitation implementation. Healthcare providers need appropriate organisational infrastructures (e.g. effective IT support) and a sufficient workflow integration to effectively implement technology[43]. They also reported that an easy-to-use interface is important, which aligns with the review's finding of needing a friendly client interface for technological improvements.

Digital Equity

Although digital equity was not part of this review's objectives, it is important to address. The included articles provide information based on the assumption that all clients (and providers) have both access and skills to engage in technology for remote assessments. Many of those currently digitally excluded are likely to become increasingly neglected from remote health services, thereby exacerbating existing health inequalities[44]. Future studies should acknowledge equity of access when examining telerehabilitation technologies. Future guidance documents should provide information for when clients do not have access to (or decline to use) telerehabilitation.

For peer review only - http://bmjopen.bmj.com/site/about/guidelines.xhtml

Comparisons with Other Studies

To our knowledge, this is the first scoping review of telerehabilitation guidance and training for assessing physical disabilities within daily clinical practice. Previous reviews mostly examine effectiveness within the context of research studies, and conclude that further provider and client training is needed to adequately implement telerehabilitation for physical assessments[45 46]. These studies conclude that telerehabiltiation technology has potential to deliver effective physical disability care, the current state of the art is insufficient for accurate remote movement assessment[47]. Our review confirms the need for further provider training, and additionally emphasises the lack of specific movement-related guidance.

Study Limitations

This scoping review has several limitations. Firstly, many telerehabilitation guidance documents for physical disabilities are likely unpublished. Although this review conducted an extensive search within grey literature sources, guidance documents and other relevant studies have likely been missed. Secondly, the search strategy was biased towards the English language. Despite no language restrictions during the search and the use of language translation software, relevant articles have likely been missed. Finally, this review was restricted by its rapid, cross-sectional approach. The authors acknowledge that some articles within this review may have been updated since the initial search and others have likely emerged since. This review may serve as a foundation for future research and development of guidance documents to provide information that addresses the gaps identified above.

CONCLUSION

There is a clear need for specific telerehabilitation guidance for physical disabilities, particularly for effective practice of remote physical assessments. Studies examining telerehabilitation technologies should deliver guidance and training to providers as well as document the context of providers if those technologies are to be effectively implemented. The catalyst that is the COVID-19 pandemic has forced the uptake of remote rehabilitation services, which are likely to persist beyond the pandemic. The development and maintenance of efficient telerehabilitation will not only require detailed guidance, but also active performance monitoring for on-going improvement of existing guidance, without which remote physical assessments may result in sub-optimal management.

Acknowledgments

We would like to sincerely thank Chris Johns from the University of Plymouth, who provided library support during the initial phase of this scoping review, and particularly assisted in developing the search strategy. Thanks to Sarah Chatfield who undertook the initial limited search of the literature.

Author Contributions

Krithika Anil planned and conducted the literature search, organised the review team, analysed and reported the results, and produced the initial manuscript. Jennifer Freeman conceptualised the study idea and supervised study processes. All authors (i.e. Krithika Anil, Jennifer Freeman, Sarah Buckingham, Sara Demain, Hilary Gunn, Ray Jones, Angela Logan, Jonathan Marsden, Diane Playford, Kim Sein, and Bridie Kent) reviewed the articles from the literature search, extracted data, contributed to data interpretation, and critically evaluated the manuscript. The corresponding author, Krithika Anil, attests that all listed authors meet authorship criteria and that no others meeting the criteria have been omitted.

Funding

This work was supported by UKRI-NIHR (MRC Section), Covid-19; Reference MR/V021060/1. The funder had no role in study design, data collection, data analysis, or writing of the report.

For peer review only - http://bmjopen.bmj.com/site/about/guidelines.xhtml

Competing Interests

None declared.

Data Sharing

No additional data available.

Dissemination to Participants and Related Patient and Public Communities

No participants were involved in this review. The research findings will be disseminated at relevant professional organisation and scientific conferences.

Ethics Statement

This study is a scoping literature review and did not involve participant data collection. Therefore, ethical approval was not required.

References

1. Peek N, Sujan M, Scott P. Digital health and care in pandemic times: impact of COVID-19. *BMJ Health and Care Informatics* 2020;27(1):e100166.
2. Brennan D, Tindall L, Theodoros D, et al. A blueprint for telerehabilitation guidelines. *Int J Telerehabil* 2010;2(2):31-34. doi: 10.5195/ijt.2010.6063
3. Charted Society of Physiotherapy. Rehabilitation and Covid-19 - CSP policy statement 2020 [Available from: https://www.csp.org.uk/professional-clinical/improvement-innovation/community-rehabilitation/rehab-covid-19-policy-statement.
4. British Society of Rehabilitation Medicine. Rehabilitation in the wake of Covid-19 - A phoenix from the ashes, 2020.
5. Wade DT. The future of rehabilitation in the United Kingdom National Health Service: Using the COVID-19 crisis to promote change, increasing efficiency and effectiveness: SAGE Publications Sage UK: London, England, 2020.
6. Wade DT. What attributes should a specialist in rehabilitation have? Seven suggested specialist Capabilities in Practice: SAGE Publications Sage UK: London, England, 2020.
7. Aromataris E, Munn Z. Chapter 1: JBI Systematic Reviews. In: Aromataris E, Munn Z, eds. JBI Manual for Evidence Synthesis: JBI 2020.
8. Tricco AC, Lillie E, Zarin W, et al. A scoping review on the conduct and reporting of scoping reviews. *BMC medical research methodology* 2016;16(1):15.
9. Peters MDJ, Marnie C, Tricco AC, et al. Updated methodological guidance for the conduct of scoping reviews. *JBI Evid Synth* 2020;18(10):2119-26. doi: 10.11124/jbies-20-00167 [published Online First: 2020/10/11]
10. Meyerson B. Top 10 emerging technologies of 2015 World Economic Forum2015 [Available from: https://www.weforum.org/agenda/2015/03/top-10-emerging-technologies-of-2015-2.
11. Wood L. Global Telepresence Robots Market (2020 to 2025) - Growth, Trends, and Forecast Business Wire2020 [Available from: https://www.businesswire.com/news/home/20201005005668/en/Global-Telepresence-Robots-Market-2020-to-2025---Growth-Trends-and-Forecast---ResearchAndMarkets.com.
12. Reuters T. EndNote X9. 2013
13. Ouzzani M, Hammady H, Fedorowicz Z, et al. Rayyan — a web and mobile app for systematic reviews. *Syst Rev* 2016(5):210. doi: 10.1186/s13643-016-0384-4
14. Kim H, Sefcik JS, Bradway C. Characteristics of Qualitative Descriptive Studies: A Systematic Review. *Res Nurs Health* 2017;40(1):23-42. doi: 10.1002/nur.21768 [published Online First: 2016/09/30]

For peer review only - http://bmjopen.bmj.com/site/about/guidelines.xhtml

15. Al Rajeh A, Steiner MC, Aldabayan Y, et al. Use, utility and methods of telehealth for patients with COPD in England and Wales: a healthcare provider survey. *BMJ Open Respir Res* 2019;6(1):e000345.
16. Middleton A, Simpson KN, Bettger JP, et al. COVID-19 Pandemic and Beyond: Considerations and Costs of Telehealth Exercise Programs for Older Adults With Functional Impairments Living at Home-Lessons Learned from a Pilot Case Study. *Phys Ther*
17. Anton D, Berges I, Bermúdez J, et al. A Telerehabilitation System for the Selection, Evaluation and Remote Management of Therapies. *Sensors (Basel)*;18(5)
18. Calvaresi D, Marinoni M, Dragoni AF, et al. Real-time multi-agent systems for telerehabilitation scenarios. *Artif Intell Med*;96:217-31.
19. Hasenohrl T, Windschnurer T, Dorotka R, et al. Prescription of individual therapeutic exercises via smartphone app for patients suffering from non-specific back pain A qualitative feasibility and quantitative pilot study. *Wien Klin Wochen*;132(5):115-23.
20. Levac D, Dumas HM, Meleis W. A Tablet-Based Interactive Movement Tool for Pediatric Rehabilitation: Development and Preliminary Usability Evaluation. *JMIR Rehabil Assist Technol*;5(2):e10307.
21. Paneroni M, Colombo F, Papalia A, et al. Is Telerehabilitation a Safe and Viable Option for Patients with COPD? A Feasibility Study. *Copd*;12(2):217-25.
22. Giesbrecht EM, Miller WC. Effect of an mHealth wheelchair skills training program for older adults: a feasibility randomized controlled trial. *Archives of Physical Medicine and Rehabilitation 2019 Nov;100(11):2159-2166* 2019
23. Grau-Pellicer M, Lalanza JF, Jovell-Fernandez E, et al. Impact of mHealth technology on adherence to healthy PA after stroke: a randomized study. *Topics in Stroke Rehabilitation 2020;27(5):354-368* 2020
24. Guerra de Oliveira Gondim IT, Cabral dos Santos Accioly Lins C, Jorge Asano NM, et al. Individualized guidance and telephone monitoring in a self-supervised home-based physiotherapeutic program in Parkinson. *Fisioterapia em Movimento [Physical Therapy in Movement] 2017 Jul-Sep;30(3):559-568* 2017
25. Venkataraman K, Morgan M, Amis KA, et al. Tele-Assessment of the Berg Balance Scale: Effects of Transmission Characteristics. *Arch Phys Med Rehabil*;98(4):659-64.e1.
26. Amatya B, Galea MP, Kesselring J, et al. Effectiveness of telerehabilitation interventions in persons with multiple sclerosis: A systematic review. *Mult Scler Relat Disord*;4(4):358-69.
27. Block VA, Pitsch E, Tahir P, et al. Remote Physical Activity Monitoring in Neurological Disease: A Systematic Review. *PLoS One* 2016;11(4):e0154335.
28. Mani S, Sharma S, Omar B, et al. Validity and reliability of Internet-based physiotherapy assessment for musculoskeletal disorders: a systematic review. *J Telemed Telecare*;23(3):379-91.
29. AHPScot. A guide to Musculoskeletal (MSK) Digital Health consultations during Coronavirus (COVID-19), 2020.
30. Health and Social Care Board. AHP Virtual Consultation Guidance, 2020.
31. Teleswallowing Ltd. Telehealth Service Delivery Guide, 2020.
32. Charted Society of Physiotherapy. Telephone guidance for musculoskeletal practice, 2020.
33. Charted Society of Physiotherapy. Digital tools to support service delivery, 2020.
34. Charted Society of Physiotherapy. COVID-19: guide for rapid implementation of remote consultations, 2020.
35. Royal College of Occupational Therapists. Virtual Assessments/Reviews: The Script. 2020
36. Royal College of Speech and Language Therapists. MAINTAINING SECURITY FOR TELEHEALTH SESSIONS, 2020.
37. East Lancashire Hospitals NHS Trust. Telepractice dysphagia assessmentsGuidelines for use with a patient in their own home/nursing home, 2020.
38. Seuren LM, Wherton J, Greenhalgh T, et al. Physical Examinations via Video for Patients With Heart Failure: Qualitative Study Using Conversation Analysis. *J Med Internet Res* 2020;22(2):e16694. doi: 10.2196/16694
39. Shaw SE, Seuren LM, Wherton J, et al. Video Consultations Between Patients and Clinicians in Diabetes, Cancer, and Heart Failure Services: Linguistic Ethnographic Study of Video-Mediated Interaction. *J Med Internet Res* 2020;22(5):e18378. doi: 10.2196/18378
40. Kuek A, Hakkennes S. Healthcare staff digital literacy levels and their attitudes towards information systems. *Health Informatics Journal* 2019;26(1):592-612. doi: 10.1177/1460458219839613

For peer review only - http://bmjopen.bmj.com/site/about/guidelines.xhtml

41. Poncette A-S, Glauert DL, Mosch L, et al. Undergraduate Medical Competencies in Digital Health and Curricular Module Development: Mixed Methods Study. *J Med Internet Res* 2020;22(10):e22161. doi: 10.2196/22161
42. Shaw J, Shaw S, Wherton J, et al. Studying Scale-Up and Spread as Social Practice: Theoretical Introduction and Empirical Case Study. *J Med Internet Res* 2017;19(7):e244. doi: 10.2196/jmir.7482
43. Palacholla RS, Fischer N, Coleman A, et al. Provider- and Patient-Related Barriers to and Facilitators of Digital Health Technology Adoption for Hypertension Management: Scoping Review. *JMIR cardio* 2019;3(1):e11951-e51. doi: 10.2196/11951
44. Majeed A, Maile EJ, Coronini-Cronberg S. Covid-19 is magnifying the digital divide: BMJ; 2020 [Available from: https://blogs.bmj.com/bmj/2020/09/01/covid-19-is-magnifying-the-digital-divide/ accessed Dec 2020.
45. Hosseiniravandi M, Kahlaee AH, Karim H, et al. Home-based telerehabilitation software systems for remote supervising: a systematic review. *International Journal of Technology Assessment in Health Care* 2020;36(2):113-25.
46. Peretti A, Amenta F, Tayebati SK, et al. Telerehabilitation: Review of the State-of-the-Art and Areas of Application. *JMIR rehabilitation and assistive technologies* 2017;4(2):e7.
47. Aderonmu JA. Emerging challenges in meeting physiotherapy needs during COVID-19 through telerehabilitation. *Bulletin of Faculty of Physical Therapy* 2020;25(1):16. doi: 10.1186/s43161-020-00018-4

For peer review only

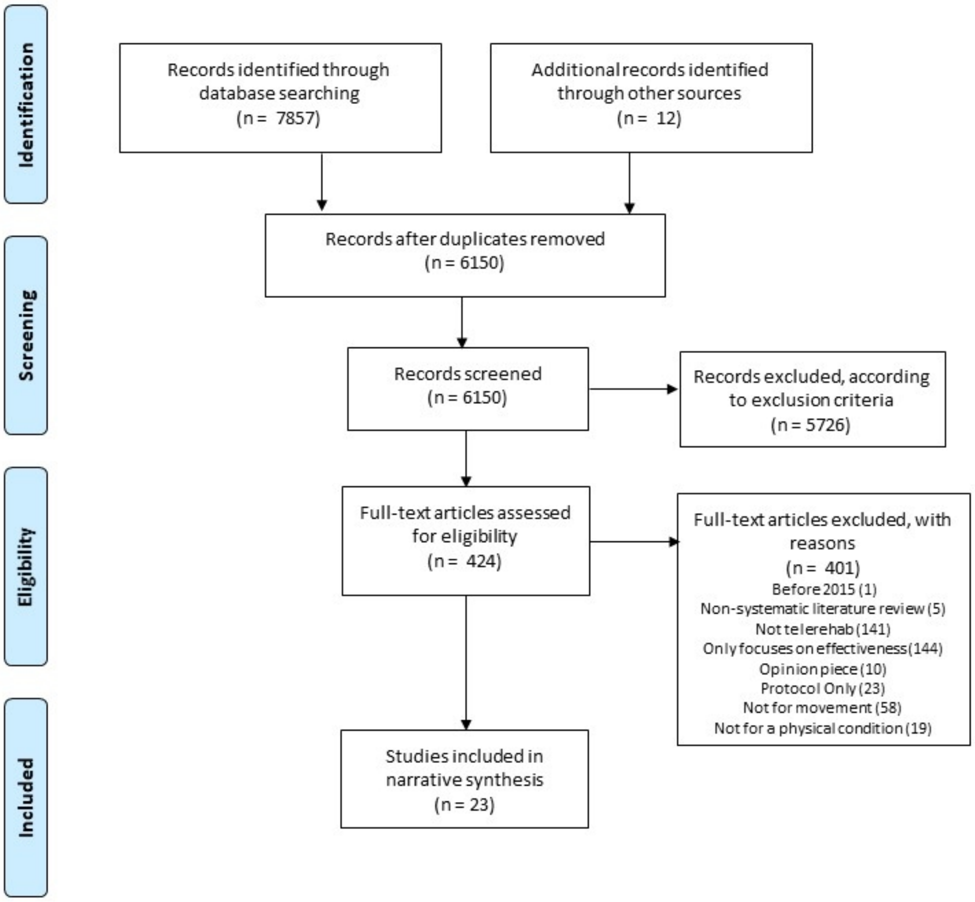

PRISMA flow diagram that charts the study identification process

192x173mm (96 x 96 DPI)

Search Strategy Terms

The below search strategy uses a logic grid and is based on the PCC framework; additional columns were added to aid in search sensitivity. As advised by an expert librarian, "concept 3" column was only used to narrow searches that produced an overwhelming quantity of results.

Population 1	*(Population 2)*	*Concept 1*	*Concept 2*	*Concept 3*	*Context*
Physical condition	Covid*	Tele*	Training	Movement	Healthcare
Physical disability	Coronavirus	Virtual	Guidance	Mobility	Health care
Physical impairment	2019-nCoV	Online	Assessment	Function	Clinic
	SARS-CoV-2	Video	Tool*	Gait	Social care
	Severe acute respiratory syndrome coronavirus 2	Digital	Protocol	Walking	Hospital
		Multimedia	Battery	Balance	Practice
		Mobile		Motor	
		Phone		Strength	
		mHealth		Power	
		eHealth		Tone	
				Range	
				Contracture	
				Fatigue	
				Stamina	
				Dexterity	
				Physical capacity	

Search term strategy:

1. Each column was searched individually, e.g. physical condition OR physical disability OR physical impairment
2. All individual searches based on each column were combined, e.g. (physical condition OR physical disability OR physical impairment)AND (Tele* OR Virtual OR Online OR Video OR Digital OR Multimedia OR Mobile OR Phone OR mHealth OR eHealth)
3. The search strategy proceeded as follows (after searching for each column individually):
   a. Column 1 AND column 3 AND column 4 AND column 5
   b. If there were too many hits based on a., column 6 wasto be added

For peer review only

   c. Column 2 was only to be used as an add-on after searches to keep a separate record of covid-related literature for the narrative synthesis
4. Unpublished literature searches used a combination of column 1 and 3 in the first instance, where searches were tailored according to individual database searching tools.
5. Date limiter: 2015 onwards.

For peer review only

Record of Online Searches

Database: PubMed
Date: 20/07/20

ID	Search Term(s)	Hits
1	(physical condition) OR (physical disability) OR (physical impairment)	641,602
2	(tele*) OR (virtual) OR (online) OR (video) OR (digital) OR (multimedia) OR (mobile) OR (phone) OR (mhealth) OR (ehealth)	862,859
3	(training) OR (guidance) OR (assessment) OR (tool*) OR (protocol) OR (battery)	5,791,855
4	(movement) OR (mobility) OR (function) OR (gait) OR (walking) OR (balance) OR (motor) OR (strength) OR (power) OR (tone) OR (range) OR (contracture) OR (fatigue) OR (stamina) OR (dexterity) OR (physical capacity)	15,600,817
5	(ID 1) AND (ID 2) AND (ID 3) AND (ID 4) with date limiter: 2015 onwards	5,322

Database: CINHAL
Date: 03/08/20

ID	Search Term(s)	Hits
1	(physical condition) OR (physical disability) OR (physical impairment)	52,385
2	(tele*) OR (virtual) OR (online) OR (video) OR (digital) OR (multimedia) OR (mobile) OR (phone) OR (mhealth) OR (ehealth)	267,357
3	(training) OR (guidance) OR (assessment) OR (tool*) OR (protocol) OR (battery)	1,100,793
4	(movement) OR (mobility) OR (function) OR (gait) OR (walking) OR (balance) OR (motor) OR (strength) OR (power) OR (tone) OR (range) OR (contracture) OR (fatigue) OR (stamina) OR (dexterity) OR (physical capacity)	902,661
5	(ID 1) AND (ID 2) AND (ID 3) AND (ID 4) with date limiter: 2015 onwards	197

Database: PsychInfo
Date: 03/08/20

ID	Search Term(s)	Hits
1	(physical condition) OR (physical disability) OR (physical impairment)	148,089
2	(tele*) OR (virtual) OR (online) OR (video) OR (digital) OR (multimedia) OR (mobile) OR (phone) OR (mhealth) OR (ehealth)	240,422

For peer review only

3	(training) OR (guidance) OR (assessment) OR (tool*) OR (protocol) OR (battery)	1,057,337
4	(movement) OR (mobility) OR (function) OR (gait) OR (walking) OR (balance) OR (motor) OR (strength) OR (power) OR (tone) OR (range) OR (contracture) OR (fatigue) OR (stamina) OR (dexterity) OR (physical capacity)	1,159,877
5	(ID 1) AND (ID 2) AND (ID 3) AND (ID 4) with date limiter: 2015 onwards	557

Database: Cochrane Library (Reviews Only)
Date: 03/08/20

ID	Search Term(s)	Hits
1	(physical condition OR physical disability OR physical impairment) AND<br><br>(tele* OR virtual OR online OR video OR digital OR multimedia OR mobile OR phone OR mhealth OR ehealth) AND<br><br>(training OR guidance OR assessment OR tool* OR protocol OR battery) AND<br><br>(movement OR mobility OR function OR gait OR walking OR balance OR motor OR strength OR power OR tone OR range OR contracture OR fatigue OR stamina OR dexterity OR physical capacity)<br><br>with date limiter: 2015 onwards	17

Database: EMBASE
Date: 05/08/20

ID	Search Term(s)	Hits
1	physical condition OR physical disability OR physical impairment	31,388
2	tele* OR virtual OR online OR video OR digital OR multimedia OR mobile OR phone OR mhealth OR ehealth	973,017
3	training OR guidance OR assessment OR tool* OR protocol OR battery	457,1408
4	movement OR mobility OR function OR gait OR walking OR balance OR motor OR strength OR power OR tone OR range OR contracture OR fatigue OR stamina OR dexterity OR physical capacity	700,1843
5	(ID 1) AND (ID 2) AND (ID 3) AND (ID 4) with date limiter: 2015 onwards	157

Database: Web of Science
Date: 05/08/20

ID	Search Term(s)	Hits
1	TS=(physical condition OR physical disability OR physical impairment)	277,584
2	TS=(tele* OR virtual OR online OR video OR digital OR multimedia OR mobile OR phone OR mhealth OR ehealth)	2,360,472
3	TS=(training OR guidance OR assessment OR tool* OR protocol OR battery)	4,974,907
4	TS=(movement OR mobility OR function OR gait OR walking OR balance OR motor OR strength OR power OR tone OR range OR contracture OR fatigue OR stamina OR dexterity OR physical capacity)	12,757,447
5	(ID 1) AND (ID 2) AND (ID 3) AND (ID 4) with date limiter: 2015 onwards	1,720

Database: PEDro
Date: 06/08/20

ID	Search Term(s)	Hits
1	Tele* AND training with date limiter: 2015 onwards	79
2	Tele* AND guidance with date limiter: 2015 onwards	12
3	mHealth AND training with date limiter: 2015 onwards	8
4	mHealth AND guidance with date limiter: 2015 onwards	1
5	eHealth AND training with date limiter: 2015 onwards	1
6	eHealth AND guidance with date limiter: 2015 onwards	0

Database: UK Health Forum
Date: 11/08/20

ID	Search Term(s)	Hits
1	Tele	0
2	Telehealth	0
3	Telemedicine	0
4	Telerehabilitation	0
5	Tele rehabilitation	0
6	mhealth	0
7	ehealth	1

For peer review only

8	Virtual	0
9	Online	4
10	Video	0
11	Digital	0
12	Multimedia	0
13	Mobile	0
14	Phone	0

Database: National Archives (UK)
Date: 06/08/20

ID	Search Term(s)	Hits
1	Tele* with date limiter: 2015 onwards	37
2	mhealth with date limiter: 2015 onwards	0
3	ehealth with date limiter: 2015 onwards	1

Within each search of this database, hits represent websites that can be searched using National Archives. Each database was searched using “training” or “guidance”; no hits were returned in any searches.

Database: WHO
Date: 11/08/20

ID	Search Term(s)	Hits
1	Telehealth AND training with date limiter: 2015 onwards	118
2	Telemedicine AND training with date limiter: 2015 onwards	353
3	Telemedicine AND guidance with date limiter: 2015 onwards	294
4	Telehealth AND guidance with date limiter: 2015 onwards	111
5	mHealth AND training with date limiter: 2015 onwards	384
6	mHealth AND guidance with date limiter: 2015 onwards	344

Database: NHS England
Date: 11/08/20

ID	Search Term(s)	Hits
1	Telehealth with date limiter: 2015 onwards	19

For peer review only

2	Telemedicine with date limiter: 2015 onwards	19
3	ehealth with date limiter: 2015 onwards	18
4	mhealth with date limiter: 2015 onwards	22

Database: Charted Society of Physiotherapy
Date: 19/08/20

ID	Search Term(s)	Hits
1	Tele*	12
2	mHealth	4
3	eHealth	23
4	Tele* AND training	3538
5	Tele* AND guidance	2849

For peer review only

Procedural Guidance for Care Delivery – Derived from Recommendations Analysis

While the information below has been derived from articles that explored movement impairment, none of the recommended actions were specific for movement impairment interventions.

Procedural Guidance	Description
Pre-Delivery<br>▪ Environment Considerations<br>▪ Session Planning<br>▪ Technology Considerations<br>▪ Preparations with Client	In preparation for a telerehabilitation session, including actions such as planning the session environment, preparing the required technology, contacting the client, etc.
During Delivery<br>▪ Start<br>▪ Throughout<br>▪ End	During a telerehabilitation session, including actions such as introducing the agenda for that session, noting technical and safety concerns, summarising the session, etc.
Post-Delivery	After a telerehabilitation session, including actions such as documenting key messages of the session, deciding whether the patient needs further/additional support, and taking breaks when needed.

Pre-Delivery

Thirteen articles[1-13] suggested actions to take before engaging in a telerehabilitation session. These actions can be divided into Preparations with the Client, Environment Considerations, Session Planning, and Technology Considerations.

- Preparations with the Client: Contact the client to assess whether they are clinically appropriate to engage in telerehabilitation (e.g. whether face-to-face or urgent care is needed instead). If telerehabilitation is deemed appropriate, ensure the client has all necessary equipment (including suitable data/WIFI accessibility) and, if required, test any equipment with the client. Provide any information the client needs to attend the telerehabilitation session (e.g. link to logon to session, passwords, times, private and well-lit room, or other resources.). Ensure the client's phone number is available in case of technological issues during the session. Consider the client's context (e.g. hard of hearing or anxious about using technology) and address the context as appropriate if possible.
- Environment Considerations: Ensure the location from which one provides the telerehabilitation is quiet, private, not cluttered and will not be disturbed. Curtains should be drawn to reduce any on-screen glare from the sun. Clients should ensure the location is well-lit without relying on the sun. Test any technology (especially video and audio) to check they are working properly. If conducting a group session, ensure the size of the class is appropriate for the activities planned.
- Session Planning: Evaluate the client's functional status prior to the session and develop a clear agenda. Consider whether a client would benefit from a group or one-to-one session. If planning a group session, ensure there is enough time to address everyone's technological issues (e.g. connectivity issues, audio issues, etc.). Consider how many other staff members you may need (e.g. an extra member for support during a group session).

For peer review only

- Technology Considerations: Ensure any technology used is appropriate for the agenda of that session. Ensure there are back-up devices that can be used (e.g. telephone) if the main technology fails. Professional and patient clients should only open the software needed for the session and all other software should be closed. Ensure internet-using technology is wired to or close to the WIFI router, and that all required equipment are close-by. Position any cameras so that the whole face is within direct view. Ensure that all providers know how to use the required technology (e.g. knowing how to record or take screen shots) prior to the session.

During Delivery

Seven articles[1, 5, 7-11] suggested actions that should occur during the telerehabilitation session, which can be divided into the Start of the session, During the session, and the End of the session.

- Start: Welcome the client and ensure audio and video are working correctly. Confirm the identity of everyone attending the session. If a trusted other (e.g. carer) is also attending, check that the client is comfortable with that trusted other. If possible, lock the meeting to prevent others from entering. During a group session, ensure everyone in the group introduces themselves (including all staff). Summarise the session's agenda and introduce the roles of any trusted other or staff (e.g. assisting with note taking). Ensure the client(s) know what to do if the session is cut. Ask the client if they have any questions or concerns before engaging in the telerehabilitation session. Ensure the client is still clinically appropriate to engage in telerehabilitation (i.e. they do not need face-to-face or urgent care). If taking screen shots or recording, ensure the client knows and you have their consent. Finally, ask the client to show their environment to ensure any rehabilitation activities can be conducted safely and accurately.
- During: Document activities as per your professional governing body requirements, yet also include any technical issues and safety concerns from the client. Be patient during technical glitches (e.g. blurred video or audio cutting out). Understand that communication is harder for the client and ensure communication is intentional (e.g. verbal cues to let the client know you are listening). Ensure that you encourage the client to engage (e.g. by asking questions). The professional should inform the client when and why they might be pre-occupied (e.g. taking notes or looking for a resource).
- End: Carefully and clearly summarise key messages from the session. Let the client know if another session is required and when that will take place. Similarly, let the client know the required details if they need to be referred to another service. Confirm and record that the client is comfortable in engaging in further telerehabilitation sessions. Ensure the client knows self-care activities and their regime. Ask the client if anything needs to be clarified. Provide the client contact details if they need to contact the provider again. When ending the call, let the client know explicitly and then end the call.

Post Delivery

Three articles[5, 10, 11] suggested actions that should occur after a telerehabilitation session. Re-evaluate the client's functional ability. Document key messages of the session as required. Consider if a follow-up call or other structural support is needed for the client. Ensure to take breaks between telerehabilitation sessions to reduce any fatigue and look after vocals.

For peer review only

References

1. AHPScot, *A guide to Musculoskeletal (MSK) Digital Health consultations during Coronavirus (COVID-19)*. 2020.
2. Anton, D., et al., *A Telerehabilitation System for the Selection, Evaluation and Remote Management of Therapies.* Sensors (Basel). **18**(5).
3. Block, V.A., et al., *Remote Physical Activity Monitoring in Neurological Disease: A Systematic Review.* PLoS One, 2016. **11**(4): p. e0154335.
4. Calvaresi, D., et al., *Real-time multi-agent systems for telerehabilitation scenarios.* Artif Intell Med. **96**: p. 217-231.
5. Health and Social Care Board, *AHP Virtual Consultation Guidance*. 2020.
6. Paneroni, M., et al., *Is Telerehabilitation a Safe and Viable Option for Patients with COPD? A Feasibility Study.* Copd. **12**(2): p. 217-25.
7. Charted Society of Physiotherapy, *COVID-19: guide for rapid implementation of remote consultations*. 2020.
8. Royal College of Occupational Therapists, *Virtual Assessments/Reviews: The Script.* 2020.
9. Royal College of Speech and Language Therapists, *MAINTAINING SECURITY FOR TELEHEALTH SESSIONS*. 2020.
10. East Lancashire Hospitals NHS Trust, *Telepractice dysphagia assessmentsGuidelines for use with a patient in their own home/nursing home*. 2020.
11. Middleton, A., et al., *COVID-19 Pandemic and Beyond: Considerations and Costs of Telehealth Exercise Programs for Older Adults With Functional Impairments Living at Home-Lessons Learned from a Pilot Case Study.* Phys Ther.
12. Charted Society of Physiotherapy, *Telephone guidance for musculoskeletal practice*. 2020.
13. Charted Society of Physiotherapy, *Digital tools to support service delivery*. 2020.

For peer review only

Preferred Reporting Items for Systematic reviews and Meta-Analyses extension for Scoping Reviews (PRISMA-ScR) Checklist

SECTION	ITEM	PRISMA-ScR CHECKLIST ITEM	REPORTED ON PAGE #
**TITLE**			
Title	1	Identify the report as a scoping review.	
**ABSTRACT**			
Structured summary	2	Provide a structured summary that includes (as applicable): background, objectives, eligibility criteria, sources of evidence, charting methods, results, and conclusions that relate to the review questions and objectives.	
**INTRODUCTION**			
Rationale	3	Describe the rationale for the review in the context of what is already known. Explain why the review questions/objectives lend themselves to a scoping review approach.	
Objectives	4	Provide an explicit statement of the questions and objectives being addressed with reference to their key elements (e.g., population or participants, concepts, and context) or other relevant key elements used to conceptualize the review questions and/or objectives.	
**METHODS**			
Protocol and registration	5	Indicate whether a review protocol exists; state if and where it can be accessed (e.g., a Web address); and if available, provide registration information, including the registration number.	
Eligibility criteria	6	Specify characteristics of the sources of evidence used as eligibility criteria (e.g., years considered, language, and publication status), and provide a rationale.	
Information sources*	7	Describe all information sources in the search (e.g., databases with dates of coverage and contact with authors to identify additional sources), as well as the date the most recent search was executed.	
Search	8	Present the full electronic search strategy for at least 1 database, including any limits used, such that it could be repeated.	
Selection of sources of evidence†	9	State the process for selecting sources of evidence (i.e., screening and eligibility) included in the scoping review.	
Data charting process‡	10	Describe the methods of charting data from the included sources of evidence (e.g., calibrated forms or forms that have been tested by the team before their use, and whether data charting was done independently or in duplicate) and any processes for obtaining and confirming data from investigators.	
Data items	11	List and define all variables for which data were sought and any assumptions and simplifications made.	
Critical appraisal of individual sources of evidence§	12	If done, provide a rationale for conducting a critical appraisal of included sources of evidence; describe the methods used and how this information was used in any data synthesis (if appropriate).	
Synthesis of results	13	Describe the methods of handling and summarizing the data that were charted.	

For peer review only

SECTION	ITEM	PRISMA-ScR CHECKLIST ITEM	REPORTED ON PAGE #
**RESULTS**			
Selection of sources of evidence	14	Give numbers of sources of evidence screened, assessed for eligibility, and included in the review, with reasons for exclusions at each stage, ideally using a flow diagram.	
Characteristics of sources of evidence	15	For each source of evidence, present characteristics for which data were charted and provide the citations.	
Critical appraisal within sources of evidence	16	If done, present data on critical appraisal of included sources of evidence (see item 12).	
Results of individual sources of evidence	17	For each included source of evidence, present the relevant data that were charted that relate to the review questions and objectives.	
Synthesis of results	18	Summarize and/or present the charting results as they relate to the review questions and objectives.	
**DISCUSSION**			
Summary of evidence	19	Summarize the main results (including an overview of concepts, themes, and types of evidence available), link to the review questions and objectives, and consider the relevance to key groups.	
Limitations	20	Discuss the limitations of the scoping review process.	
Conclusions	21	Provide a general interpretation of the results with respect to the review questions and objectives, as well as potential implications and/or next steps.	
**FUNDING**			
Funding	22	Describe sources of funding for the included sources of evidence, as well as sources of funding for the scoping review. Describe the role of the funders of the scoping review.	

JBI = Joanna Briggs Institute; PRISMA-ScR = Preferred Reporting Items for Systematic reviews and Meta-Analyses extension for Scoping Reviews.

* Where *sources of evidence* (see second footnote) are compiled from, such as bibliographic databases, social media platforms, and Web sites.

† A more inclusive/heterogeneous term used to account for the different types of evidence or data sources (e.g., quantitative and/or qualitative research, expert opinion, and policy documents) that may be eligible in a scoping review as opposed to only studies. This is not to be confused with *information sources* (see first footnote).

‡ The frameworks by Arksey and O'Malley (6) and Levac and colleagues (7) and the JBI guidance (4, 5) refer to the process of data extraction in a scoping review as data charting.

§ The process of systematically examining research evidence to assess its validity, results, and relevance before using it to inform a decision. This term is used for items 12 and 19 instead of "risk of bias" (which is more applicable to systematic reviews of interventions) to include and acknowledge the various sources of evidence that may be used in a scoping review (e.g., quantitative and/or qualitative research, expert opinion, and policy document).

From: Tricco AC, Lillie E, Zarin W, O'Brien KK, Colquhoun H, Levac D, et al. PRISMA Extension for Scoping Reviews (PRISMAScR): Checklist and Explanation. Ann Intern Med. 2018;169:467–473. doi: 10.7326/M18-0850.

For peer review only

BMJ Open

The scope, context, and quality of telerehabilitation guidelines for physical disabilities: a scoping review

Journal:	*BMJ Open*
Manuscript ID	bmjopen-2021-049603.R2
Article Type:	Original research
Date Submitted by the Author:	08-Jul-2021
Complete List of Authors:	Anil, Krithika; University of Plymouth, School of Health Professions<br>Freeman, Jennifer; Peninsula Allied Hlth Ctr, School of Health Professions<br>Buckingham, Sarah; University of Plymouth, School of Health Professions<br>Demain, Sara; Plymouth University, School of Health Professions<br>Gunn, H; University of Plymouth, School of Health Professions<br>Jones, Ray; Plymouth University, School of Nursing and Midwifery<br>Logan, Angela; Royal Devon and Exeter NHS Foundation Trust<br>Marsden, Jonathan; Plymouth University, School of Health Professions<br>Playford , Diane ; University of Warwick, Warwick Medical School<br>Sein, Kim; University of Plymouth, School of Health Professions<br>Kent, Bridie; Plymouth University, Nursing and Midwifery, Innovations in Health and Social Care: A JBI Centre of Excellence
**Primary Subject Heading**:	Health services research
Secondary Subject Heading:	Health services research, Rehabilitation medicine
Keywords:	COVID-19, Telemedicine < BIOTECHNOLOGY & BIOINFORMATICS, Physiology < NATURAL SCIENCE DISCIPLINES, Protocols & guidelines < HEALTH SERVICES ADMINISTRATION & MANAGEMENT, REHABILITATION MEDICINE

SCHOLARONE™
Manuscripts

I, the Submitting Author has the right to grant and does grant on behalf of all authors of the Work (as defined in the below author licence), an exclusive licence and/or a non-exclusive licence for contributions from authors who are: i) UK Crown employees; ii) where BMJ has agreed a CC-BY licence shall apply, and/or iii) in accordance with the terms applicable for US Federal Government officers or employees acting as part of their official duties; on a worldwide, perpetual, irrevocable, royalty-free basis to BMJ Publishing Group Ltd ("BMJ") its licensees and where the relevant Journal is co-owned by BMJ to the co-owners of the Journal, to publish the Work in this journal and any other BMJ products and to exploit all rights, as set out in our licence.

The Submitting Author accepts and understands that any supply made under these terms is made by BMJ to the Submitting Author unless you are acting as an employee on behalf of your employer or a postgraduate student of an affiliated institution which is paying any applicable article publishing charge ("APC") for Open Access articles. Where the Submitting Author wishes to make the Work available on an Open Access basis (and intends to pay the relevant APC), the terms of reuse of such Open Access shall be governed by a Creative Commons licence – details of these licences and which Creative Commons licence will apply to this Work are set out in our licence referred to above.

Other than as permitted in any relevant BMJ Author's Self Archiving Policies, I confirm this Work has not been accepted for publication elsewhere, is not being considered for publication elsewhere and does not duplicate material already published. I confirm all authors consent to publication of this Work and authorise the granting of this licence.

For peer review only

Title Page

Manuscript Title: The scope, context, and quality of telerehabilitation guidelines for physical disabilities: a scoping review

Word Count: 3958

Authors: Krithika Anil[1] (0000-0002-8027-1665), Jennifer A Freeman[2], Sarah Buckingham[3], Sara Demain[4], Hilary Gunn[5], Ray B Jones[6], Angela Logan[7], Jonathan Marsden[8], E Diane Playford[9], Kim Sein[10], Bridie Kent[11,12]

[1] Research Fellow; Peninsula Allied Health Centre, School of Health Professions, University of Plymouth, Derriford Road, Plymouth, PL6 8BH, United Kingdom; krithika.anil@plymouth.ac.uk

[2] Professor of Physiotherapy and Rehabilitation, Peninsula Allied Health Centre, School of Health Professions, University of Plymouth, Derriford Road, Plymouth, PL6 8BH, United Kingdom; jenny.freeman@plymouth.ac.uk; +44 1752 588835

[3] Research Fellow; Peninsula Allied Health Centre, School of Health Professions, University of Plymouth, Derriford Road, Plymouth, PL6 8BH, United Kingdom; sarah.buckingham@plymouth.ac.uk

[4] Head of School of Health Professions, Peninsula Allied Health Centre, School of Health Professions, University of Plymouth, Derriford Road, Plymouth, PL6 8BH, United Kingdom; sara.demain@plymouth.ac.uk; +44 1752 587570

[5] Associate Professor in Physiotherapy, Peninsula Allied Health Centre, School of Health Professions, University of Plymouth, Derriford Road, Plymouth, PL6 8BH, United Kingdom; hilary.gunn100@plymouth.ac.uk; +44 1752 588825

[6] Professor of Health Informatics, School of Nursing and Midwifery, University of Plymouth, Drake Circus, Plymouth, PL4 8AA, United Kingdom; ray.jones@plymouth.ac.uk; +44 1752 586532

[7] Consultant Physiotherapist, Stroke Rehabilitation, Royal Devon and Exeter NHS Foundation Trust, Barrack Road, Exeter, Devon, EX2 5DW, United Kingdom; angie.logan@nhs.net

[8] Professor and Chair in Rehabilitation, Peninsula Allied Health Centre, School of Health Professions, University of Plymouth, Derriford Road, Plymouth, PL6 8BH, United Kingdom; jonathan.marsden@plymouth.ac.uk; +44 1752 587590

[9] Honorary Professor of Neurological Rehabilitation, Warwick Medical School, Gibbet Hill Campus, University of Warwick, CV4 7AL, United Kingdom; d.playford@warwick.ac.uk

[10] Research Fellow; Peninsula Allied Health Centre, School of Health Professions, University of Plymouth, Derriford Road, Plymouth, PL6 8BH, United Kingdom; kim.sein@plymouth.ac.uk

[11] Professor of Leadership in Nursing, School of Nursing and Midwifery, University of Plymouth, Drake Circus, Plymouth PL4 8AA, United Kingdom; bridie.kent@plymouth.ac.uk; +44 1752 588817

[12] Professor of Leadership in Nursing, Director of the University of Plymouth Centre for Innovations in Health and Social Care: A JBI Centre of Excellence, University of Plymouth, Drake Circus, Plymouth PL4 8AA, United Kingdom; bridie.kent@plymouth.ac.uk; +44 1752 588817

For peer review only - http://bmjopen.bmj.com/site/about/guidelines.xhtml

Corresponding author: Krithika Anil
Contact email: krithika.anil@plymouth.ac.uk

ABSTRACT

Objective To identify the available guidance and training to implement telerehabilitation movement assessments for people (adults and children) with a physical disability, including those recovering from COVID-19

Design Rapid scoping review

Included Sources and Articles PubMed, CINAHL, PsychInfo, Cochrane, EMBASE, Web of Science, PEDro, UK Health Forum, WHO, National Archives, and NHS England, searched using the Participant-Concept-Context (PCC) framework from 2015 to August 2020. Primary studies that recruited individuals with physical disabilities and guidance documents aimed at providers to implement movement-related telerehabilitation were included.

Results 23 articles (11 primary research studies, 3 systematic reviews and 9 guidance documents) were included out of 7857 that were identified from the literature search. Two main issues were found: (1) telerehabilitation guidance (from both research studies and guidance documents) was not specific to movement-related assessment, and (1) most primary research studies provided neither guidance nor training of movement-specific assessment to practitioners. Of the COVID-19-related guidance, two articles reported COVID-19 management that only referred to identifying COVID-19 status without references to specific movement-related guidance.

Conclusions Telerehabilitation guidance and training have existed pre-COVID-19, yet the lack of specific movement-related information and provider support is surprising. This gap must be addressed to optimise effective implementation of remote assessments for those with physical disabilities.

Review Registration Open Science Framework: osf.io/vm6sp

Key words: Telerehabilitation, physical disability, remote consultation, COVID-19, scoping review, guidance

STRENGTHS AND LIMITATIONS

- A wide variety of articles were reviewed due to extensive searches for grey literature and no language restrictions
- Despite no language restrictions, included articles were biased towards the English language
- Some articles within this review may have been updated since the initial search and others have likely emerged since

For peer review only

INTRODUCTION

Since the outbreak of the COVID-19 pandemic, health services across the world have rapidly adapted to a new way of working. They have embraced the need to use a "digital first" approach to maintain delivery of care as best as possible, whilst protecting both staff and patients[1]. In the field of rehabilitation this approach is typically referred as "telerehabilitation", defined as: The delivery of rehabilitation services via information and communication technologies[2]. This includes a broad range of services from assessment and monitoring to education and consultation. There is a general acknowledgement that, beyond the pandemic, remote delivery of services will be an integral part of everyday practice within future health and social care systems. Ensuring that the delivery of such services is both effective and equitable is pertinent, given the identified "tsunami" of rehabilitation need[3 4] for people with a wide range of conditions from across the life-span (including those recovering from COVID-19[5]). It is especially important to note that the practical application of telerehabilitation will not be the same across all conditions. More complicated conditions (e.g. those with co-morbidity) will likely require additional support than less complicated conditions. This additional consideration further demonstrates the need for comprehensive training and guidance for telerehabilitation.

Key to targeting an effective and personalized rehabilitation plan is the need for a comprehensive, detailed, and valid assessment[6], with ongoing monitoring and evaluation as needs change. When the primary concerns relate to physical disability, an assessment of aspects such as dynamic posture, balance and movement is fundamental. This therefore raises important questions related to how these assessments can be undertake remotely, whilst ensuring they are both safe and effective. For instance, is it possible to adequately and safely assess the balance of an individual who is falling/at risk of falls? Or the source of shoulder pain? Or the relative impact of spasticity and weakness on movement? Do the additional practical challenges such as slow internet speeds and static and restricted camera angles mean that an assessment of dynamic movement is too restricted to inform management plans? Are the clinical skills required for this type of remote assessment different to those utilised for face-to-face assessment? And if so, is there specific guidance and training related to these aspects of remotely based physical assessments to assist practitioners with implementing this effectively and efficiently? It was our observation that questions such as these have been raised by rehabilitation organisations, professional networks and individual practitioners and require urgent answers, hence the need for this rapid scoping review.

This scoping review formed part of an overall research project aimed at developing and evaluating a training package to support the remote assessment and management of people with physical disability (UK RI-NIHR, MRC, Covid-19: MR/V021060/1). It was not appropriate to involve patients or the public in the design, or conduct, or reporting of this research.

Review question and objectives

What guidance and training is available to implement telerehabilitation movement assessment for people (children and adults) with a physical disability, including those recovering from COVID-19? The review objectives were to identify:

- The contexts and types of telerehabilitation assessments delivered in a health and social care setting for people with a physical disability (including those recovering from COVID-19)
- Any specific guidance and/or training available for delivering telerehabilitation movement-related assessments
- Specific recommendations regarding movement-related assessments for effective implementation of telerehabilitation guidance

For peer review only - http://bmjopen.bmj.com/site/about/guidelines.xhtml

METHODS

Type of Review

This scoping review used the JBI approach[7]. This approach seeks to explore the breadth or extent of the literature, map and summarise the evidence, and inform future research[8]. The PRISMA checklist for scoping reviews was used for the review (see supplementary material titled "SM – PRISMA Statement for Scoping Reviews"). There is no registration database specific for scoping reviews, thus the review was uploaded to Open Science Framework: osf.io/vm6sp.p9

Patient and Public Involvement

No patients involved.

Inclusion Criteria

The inclusion criteria were developed using the PCC (participant-concept-context) framework[9].

Participants

The review considered articles that included individuals of all ages with a physical disability. Physical disability was defined as any physical condition that affects a person's mobility, physical capacity, stamina, or dexterity. The review included studies of participants with a physical disability who had any duration and severity of physical disability. There was no age restriction. Guidance and policy documents aimed at undertaking telerehabilitation with individuals with physical disabilities were also included.

Concept

Studies and policies that examined or described guidance and training regarding implementation of telerehabilitation movement assessments were considered. *Guidance* included checklists, tools, outcome measurement batteries, protocols, guidelines, risk assessments, exemplars, and appraisals. *Training* included interactions, communications, and resources with the aim to improve and/or inform the process of delivering telerehabilitation. Guidance and training must have involved movement evaluation; that is, the evaluation of how people move around, perform, and function. This included self-administered and practitioner administered telerehabilitation. Studies and policies that did not involve movement evaluation were excluded.

Context

This review considered studies that provided guidance and training in health and social care contexts, that is, telerehabilitation must have been provided by a practitioner, system, or institute.

Types of Literature

The following study designs were included: randomised controlled trials (RCTs), non-RCTs, quasi-experimental, pre- and post- studies, case studies, observational studies, systematic reviews, and qualitative studies. Grey literature regarding policies from recognised UK-based national and international institutes were also considered. Opinion pieces, non-systematic literature reviews, and other grey literature were excluded. No language restrictions were implemented.

Date Restriction

Publications from 2015 to Aug 2020 were included in this review because technologies and related-guidance older than this period were considered out-of-date due to the rapid speed of technology development in areas such as robotics, artificial intelligence, and telepresence[10 11].

For peer review only - http://bmjopen.bmj.com/site/about/guidelines.xhtml

Search Strategy

An initial limited search of PubMed and CINAHL was undertaken to estimate the volume of relevant literature and to identify key words to assist in developing search terms. A second search using the developed search terms was undertaken (supported by a review-specialist librarian) and adapted across each included information source (PubMed, CINAHL, PsychInfo, Cochrane, EMBASE, Web of Science, PEDro, UK Health Forum, WHO, National Archives, NHS England). Due to the rapid nature of this review, study authors were not contacted for further information in cases of ongoing or uncompleted studies. Please see supplementary materials (SM1 and SM2) for search strategy terms.

Study Selection

Following the search, all identified references were imported into Endnote[12] (a reference management software). After removal of duplications within EndNote, references were uploaded to the online Rayyan tool[13] (a review organisation tool). Titles and abstracts were screened for assessment against the inclusion criteria for the review. The full text of potentially eligible studies were retrieved and assessed in detail against the inclusion criteria. Full text studies that did not meet the inclusion criteria were excluded and are displayed in a PRISMA flowchart (see Figure 1). Any disagreements that arose between the reviewers were resolved through discussion. As this review is a rapid scoping review no quality or risk of bias assessment was undertaken.

Data Extraction

Data extraction of included studies was conducted by the review team. Data was charted using a customised Excel spreadsheet. The data extracted specific information pertaining to the review objectives and key information for each article were charted as per JBI Guidance[7]. This included telerehabilitation contexts, types of telerehabilitation, guidance and training, and any recommendations for implementing telerehabilitation. Any disagreements between reviewers were resolved through discussion.

Data Synthesis

The narrative synthesis of the findings from included studies, policies, and guidance documents was structured according to the review objectives. Findings were presented narratively, aided by appropriate tables and figures. Recommendations in the literature were analysed using descriptive qualitative analysis[14] to identify common themes. Only recommendations focusing on clinical implementation or technological improvements were analysed (recommendations for future research within the included studies were not analysed).

Mentions of the 'provider' refer to the individual providing the telerehabilitation, such as health or social care practitioners. Mentions of the 'client' refer to the individual receiving the telerehabilitation, i.e. the patient.

RESULTS

Twenty-three articles were included in the scoping review analysis (Figure 1; see Table 1 for article characteristics). Eleven[15-25] of the included articles were original research studies and three were systematic review studies[26-28]. The remaining nine[29-37] were guidance documents produced by health institutions or health experts. Guidance documents are highlighted in grey and systematic review articles are highlighted in yellow in Table 1. Eleven articles were specified for adults[15-19 21-24 26 27] (≥18 years), one article was specified for children[20] (≤18 years), and 11 articles did not specify age[25 28-37].

[Insert Figure 1 (Legend: Figure 1 PRISMA flow diagram that charts the study identification process)]

For peer review only - http://bmjopen.bmj.com/site/about/guidelines.xhtml

Table 1 Characteristics of the included articles

Author(s)	Date	Aim	Country Origin	Design	Total N	Physical Condition
Al Rajeh et al[15]	2019	Explore the use of telehealth and assess the perceptions of clinicians employing telehealth and specifically alarm settings on technology	UK	Cross-sectional	65	Chronic obstructive pulmonary disease
Amatya et al[26]	2015	Investigate the effectiveness and safety of telerehabilitation	Europe and USA	Systematic review	564	Multiple sclerosis
Anton et al[17]	2018	Develop and examine the Kinext Telerehabilitation System for physical conditions	Spain and Australia	Feasibility	18	Shoulder disorder; Hip replacement
Block et al[27]	2016	Perform a systematic review of studies using remote physical activity monitoring in neurological diseases	USA	Systematic review	943	Neurological diseases
Calvaresi et al[18]	2019	Examine the feasibility of using Artificial Intelligence for telerehabilitation	Italy	Mixed-methods	10	General physical conditions
Giesbrecht & Miller[22]	2019	Evaluate the effect of a mHealth wheelchair skills training program on clinical outcomes among older adult manual wheelchair clients	Canada	RCT	18	Physical disability needing wheelchair
Grau-Pellicer et al[23]	2020	Investigate the effectiveness of a mHealth app	Spain	RCT	41	Ischaemic or haemorrhagic stroke
Guerra de Oliveira Gondim et al[24]	2017	Evaluate the effects of individualized orientation and monitoring by telephone in a self-supervised home therapeutic exercise program on signs and symptoms of Parkinson’s disease and quality of life	Brazil	RCT	28	Parkinson's disease

For peer review only - http://bmjopen.bmj.com/site/about/guidelines.xhtml

Hasenohrl et al[19]	2020	Assess the feasibility and acceptance of orthopaedists prescribing individualised therapeutic exercises via a smartphone app to patients	Austria	Qualitative	27	Back pain
Levac et al[20]	2018	Develop and conduct a usability evaluation of the Fun, Interactive Therapy Board (FITBoard), a movement toy for children with disabilities	USA	Observational	7	General movement conditions
Teleswallowing Ltd[31]	2020	Provide evidence-based guidance on designing and establishing telerehabilitation services for dysphagia	UK	Guidance	N/A	Dysphagia
Mani et al[28]	2017	Systematically explore and summarise the validity and reliability of a telerehabilitation -based physiotherapy assessment	Malaysia	Systematic review	NS	MSK conditions
Middleton et al[16]	2020	Describe the process and cost of delivering a synchronous telehealth exercise programme	USA	Observational	1	Stroke, hypertension, and diabetes
Paneroni et al[21]	2015	Test the feasibility, adherence and satisfaction of a home-based reinforcement telerehabilitation programme	Italy	Observational	36	Chronic obstructive pulmonary disease
Charted Society of Physiotherapy[33]	2020	Provide guidance for physiotherapists on digital tools to support service delivery	UK	Guidance	N/A	General physical conditions
Charted Society of Physiotherapy[32]	2020	Provide guidance for physiotherapists on telephone triage of patients with musculoskeletal conditions	UK	Guidance	N/A	MSK conditions

For peer review only - http://bmjopen.bmj.com/site/about/guidelines.xhtml

Charted Society of Physiotherapy[34]	2020	Provide practical advice for physiotherapists and support workers to implement remote consultations	UK	Guidance	N/A	General physical conditions
Royal College of Speech and Language Therapists[36]	2020	Provide brief tips for telerehabilitation implementation revolved around security	UK	Guidance	N/A	General physical conditions
East Lancashire Hospitals NHS Trust[37]	2020	Provide brief tips for telerehabilitation implementation for dysphagia	UK	Guidance	N/A	Dysphagia
Venkataraman et al[25]	2016	Compare Berg Balance Scale ratings using videos with differing transmission characteristics with direct in-person rating	USA	Psychometrics study	45	General motor control condition
Health and Social Care Board NI[30]	2020	Amalgamate evidence and guidance on the conduct of virtual consultations so that it is easily accessible for AHPs	UK	Guidance	N/A	General physical conditions
AHPScot[29]	2020	Guide AHPs in NHS Scotland during remote consultations (telephone or video) with MSK patients during the current COVID19 pandemic	UK	Guidance	N/A	MSK conditions
Royal College of Occupational Therapists[35]	2020	Guide occupational therapists regarding implementation of virtual assessments and reviews	UK	Guidance	N/A	General physical conditions

Grey highlighted rows indicate a guidance document
N/A = Not applicable
NS = Not specified
AHPs = Allied health professionals
MSK = Musculoskeletal
UK = United Kingdom
USA = United States of America

For peer review only - http://bmjopen.bmj.com/site/about/guidelines.xhtml

The 14 included research studies and systematic reviews had the following general aims: one study assessed use and perceptions of telerehabilitation[15]; one study examined costs of implementing telerehabilitation[16]; one study assessed the development of a telerehabilitation technology[17]; three studies systematically reviewed telerehabilitation literature[26-28]; four studies assessed the feasibility of a telerehabilitation technology[18-21]; and four studies examined the effectiveness of the telerehabilitation technology[22-25].

Contexts

Client setting was reported in 13 of the 23 articles, where only one article did not include a home setting and instead only used general clinical sites[20] (Table 2). The client support environment (i.e. who was with the client) was reported in 13 articles (Table 2), where most clients were alone (n=5). Only one article specifically reported that carers were present during the consultation or intervention. Table 3 shows the wide range of providers using telerehabilitation in people with physical disability, most being a physiotherapist. Of note, all non-qualified assistants were accompanied by a qualified health professional throughout the telerehabilitation sessions. This article[20] described the development and preliminary usability, from the provider's perspective, of a tablet-based interactive movement tool. Provider setting was reported in six articles and all were telerehabilitation technologies used by providers in secondary care (e.g. an out-patient clinic;[17 19 21-23 25]).

For peer review only - http://bmjopen.bmj.com/site/about/guidelines.xhtml

Table 2 Cross-tabulation of Client Support and Client Setting information extracted from included articles

		Article Type	**Client Support Environment** Not specified	Client with group	Client alone	Client with carer
**Client Setting**	*Not specified*	Primary Research	2[15 17]	-	1[25]	-
		Reviews	-	*1[28]	*1[28]	-
		Guidance Documents	6[31 32 34-37]	-	-	-
	At home	Primary Research	-	-	4[16 19 21 22]	1[24]
		Reviews	-	-	-	-
		Guidance Documents	-	-	-	-
	Home and community	Primary Research	1[18]	-	-	-
		Reviews	-	*2[26 27]	*2[26 27]	-
		Guidance Documents	1[33]	*2[29 30]	*2[29 30]	-
	General clinic sites	Primary Research	1[20]	-	-	-
		Reviews	-	-	-	-
		Guidance Documents	-	-	-	-
	Rehabilitation unit and at home	Primary Research	-	1[23]	-	-
		Reviews	-	-	-	-
		Guidance Documents	-	-	-	-

*Five articles (3 reviews[26-28] and 2 guidance documents[29 30]) had participants complete the telerehabilitation alone and in a group - these articles have been added to both the "Client was Alone" and "Client with Group" categories.

For peer review only

Table 3 List of telerehabilitation administrators extracted from included articles

Telerehabilitation Administrator*	N[a]	N[b]	N[c]
Physiotherapist	9[15-18 20 21 23-25]	1[28]	1[34]
Occupational therapist	3[20 22 25]	-	1[35]
Medic	3[15 19 21]	1[27]	-
Nurse	2[15 21]	-	-
Speech and language therapist	-	-	2[31 37]
General clinicians	-	-	2[32 33]
AHPs	-	-	2[29 30]
Not specified	1[36]	1[26]	-
Assistants	2[16 25]	-	-
Physiologist	1[15]	-	-

AHP = Allied Health Professional, specific profession not stated
*Telerehabilitation administrators overlap in articles, therefore total N does not sum to total included articles
[a]N for primary research studies
[b]N for systematic review studies
[c]N for guidance documents

Presence of COVID-19

Only articles published in 2020[16 19 23 29-37] (n = 12) were considered for COVID-19-specific information. Of these, two articles refer to COVID-19-related management; guidance documents developed by AHPScot [29] and the Health and Social Care Board Northern Ireland[30]. The COVID-19-related recommendations from these guides are detailed in the Recommendations section. It is noteworthy that these guidance documents refer to COVID-19 in general and are not specifically related to telerehabilitation use in people with movement impairment, despite these documents generally considering the use of telerehabilitation for this purpose. Teleswallowing Ltd.[31], Middleton et al[16], and two articles from the Charted Society of Physiotherapy[33 34] also refer to COVID-19 but only state that telerehabilitation is useful to decrease the spread of COVID-19; these articles do not refer to management of patients with COVID-19.

Types of Telerehabilitation

Table 4 shows the aims of the telerehabilitation interventions examined in the primary research studies. Nine guides[29-37], 3 systematic reviews[26-28], and 2 primary studies[18 25] are excluded from Table 4 as they did not evaluate a specific telerehabilitation technology. Most (5 of the 9 studies) telerehabilitation interventions aimed to assist in management of a movement condition; however, some cited additional aims such as optimising adherence[15], improving quality of life[24], monitoring, and assessing client satisfaction[23]. One intervention provided training in using a wheelchair[22].

We aimed to summarise the various platforms used for delivery of telerehabilitation but this proved difficult, as some articles did not have sufficient detail. For example, one article[16] reports a tablet app but does not specify whether video was involved or whether it just used a chat/telephone function. Accepting these limitations, the most common delivery system appeared to be via video (e.g. video call, Skype, video-recording, etc.), followed by apps (on tablets or mobiles) and telephone calls. Fifteen of the telerehabilitation platforms were synchronous[16 17 19 20 22-24 28-31 33 35-37] (i.e. in real-time) and seven

For peer review only - http://bmjopen.bmj.com/site/about/guidelines.xhtml

platforms used both synchronous and asynchronous[15 18 21 26 27 32 34] (i.e. not in real-time) methods. Only one article used only an asynchronous platform where providers assessed physical ability from a video-recording of the client[25].

Table 4 List of telerehabilitation aims and platforms reported in the included articles

Telerehabilitation Aim	N	References
MCM	5	[16 17 19-21]
MCM and adherence	1	[15]
MCM and quality of life	1	[24]
MCM and client satisfaction	1	[23]
Training for assistive device use	1	[22]

MCM = Movement condition management

Of the 11 primary research studies, five[15 17-19 22] specified neither number of sessions nor session duration, one specified just number and one specified just duration. The number of sessions ranged from 1 to 36 (n = 2 for 1 session[20 25]; n = 1 for 16 sessions[23]; n = 1 for 28 sessions[21]; n = 2 for 36 sessions[16 24]). Three studies included 60 minute sessions[20 23 24], one included 50 minute sessions[16], and one included 100 minute sessions[21]. The variability seen here is reflective of that seen in face-to-face rehabilitation sessions.

Guidance

Specific guidance on how to use telerehabilitation technology was supplied to providers in 12 articles[20 22 25 29-37], of which three were studies[20 22 25] and the remaining were guidance documents[29-37]. A guidebook or manual was provided in ten articles[20 22 25 29-31 33-35 37], while two provided a checklist[32 36]. No guidance was supplied in seven articles[15 16 19 21 23 24], and five studies did not mention whether guidance was provided or not[17 18 26-28].

Training

Guidance documents and systematic reviews were not included in training analysis as these articles had no participants to whom to supply training. Specific training on how to use telerehabilitation technology was supplied to providers in 4 out of 11 primary research studies[16 20 22 25]. The training was provided in-person in three of these studies [16 20 22], while the remaining study provided training via a video tutorial[25]. No training was supplied in five studies[15 19 21 23 24], and 2 studies did not mention training[17 18].

Recommendations

Six themes and 11 subthemes were identified within the recommendations: Administrative Advice, Challenges, Governance, Social Support, Technology Improvements, and COVID-19 (see Table 5). Recommendations were also analysed to extract procedural guidance that providers can perform in preparation for, during, and after a telerehabilitation session. This guidance is summarised in supplementary materials (SM3) as it extended the review beyond its key message.

For peer review only - http://bmjopen.bmj.com/site/about/guidelines.xhtml

Table 5 Summary of themes identified during qualitative analysis of article recommendations

Analysis Themes	Description
Administrative Recommendations<br>▪ General<br>▪ Client-Specific<br>▪ Technology-Specific<br>▪ Data Security	Advice revolved around administrative duties, such as record keeping, technology maintenance, or data security
Challenges<br>▪ Care Provision<br>▪ Social Factors<br>▪ Technology Issues	Challenges that providers may encounter when conducting telerehabilitation services
Governance	Ethical advice, such as client consent and/or assessing confidentiality
Support Mechanisms<br>▪ Providers<br>▪ Carers<br>▪ Provider Assistants<br>▪ Non-Clinical Staff	Advice that emphasised the important role of support from various sources involved in providing telerehabilitation care to the client
Technology Improvements	Suggestions that may improve the use and function of a telerehabilitation technology
COVID-19	Actions providers are advised to conduct to address COVID-19 risks

Administrative Recommendations

Three articles[30 32 36] provided advice on administrative duties that providers should perform when conducting telerehabilitation. The advice revolved around four sub-themes:

(1) General Advice involved general administrative duties such as maintaining record-keeping standards and identifying the specific resources a provider will need when engaging in telerehabilitation (e.g. number of sessions needed, appropriate length of session considering technology set-up, clinical outcome measures needed, etc.).

(2) Client-Specific Advice involved duties that required interactions with the client, such as documenting all client conversations and noting care that would have been provided if the intervention was delivered face to face but was not done so due to the restrictions of telerehabilitation.

(3) Technology-Specific Advice involved ensuring technology was up-to-date and that the latest version of any software was being used.

(4) Data Security emphasised that providers must minimise identifiable information that is shared digitally, ensure that all actions comply with information governance, and only use technology recognised by the institute through which they provide their care services.

Challenges

Six articles[15 19 23 25 28 30] highlighted challenges when conducting telerehabilitation, which involved three sub-themes:

(1) Care Provision considers the resources needed for telerehabilitation, highlighting that some clinical assessments may be too complicated to conduct effectively at a distance and that, if this complication is not recognised by inexperienced telerehabilitation providers, the quality of care provided may be reduced.

For peer review only - http://bmjopen.bmj.com/site/about/guidelines.xhtml

(2) Social Factors identified social challenges, where unsatisfactory video etiquette, minimal rapport-building, and socio-economic disadvantages have the potential to reduce the quality of care provided via telerehabilitation.
(3) Technology Issues relate to technology failures or interruptions, such as inadequate internet connection, low camera/audio quality, and false alarms (on wearable devices) that result in a reduced quality of care.

Governance
Six articles[29 30 34-37] provided recommendations relating to governance issues. The guidance highlighted that all clients must be informed about the following: telerehabilitation is voluntary; potential risks when engaging in telerehabilitation; what will happen with their data; and what reasonable actions they should take to ensure the security of their technology. This theme also emphasised the importance of consent, where informed consent must be taken explicitly from clients when video/audio recording or taking screen shots. If required, clients must be asked whether they consent for their trusted others (e.g. carer or family) to attend the session with them. However, one article [30] stated that consent was implied when clients enter the telerehabilitation session.

Support Mechanisms
Six articles[21 23 24 28 31 37] recommended support mechanisms to be set in place for clients when providing telerehabilitation. This support involved four sub-themes:
(1) Providers to offer support beyond the main goals of telerehabilitation, such as reassurance to clients who are anxious using technology for their rehabilitation.
(2) Carers to offer clients in-person support during delivery of telerehabilitation sessions. For instance, provision of physical assistance to safely enable the client to achieve correct posture or movement, or to assist the client with using the technology, such as positioning of the camera.
(3) Provider Assistants to assist qualified staff by visiting clients in person to assist telerehabilitation, such as undertaking outcome measures face-to-face, when required and safe to do so.
(4) Non-Clinical Staff, such as administrators and senior management, to provide essential support in successful coordination and implementation of telerehabilitation, emphasising the importance of effective communication between support staff and providers.

Technology Improvements
Eight articles[15 17-20 26-28] recommended technology improvements for telerehabilitation purposes. For instance, communications should be synchronous and technology must have a client-friendly interface that satisfies both the client and the provider. Wearable technologies relevant to posture and movement (e.g. to measure steps or heart rate) must be precise. Technology would especially benefit from improved monitoring capabilities, such as real-time movement tracking and activity adherence monitoring. If technology provided smart data (e.g. real-time measures of muscle strength), it should be accompanied by useful and actionable information. Reminders to engage in self-care activities would be useful for clients during their rehabilitation period. A built-in anatomical map to assist clients in showing the location of pain, tenderness, or other sensations was also suggested.

COVID-19
Two articles (both guidance documents[29 30]) made recommendations to address COVID-19 concerns. When establishing whether the client needs telerehabilitation or face-to-face care, professionals were advised to identify the client's COVID-19 status. Suggested identifications included: tested positive and self-isolating, has symptoms and self-isolating, has come into contact with someone who tested positive and is now self-isolating, shielding, or no current cautions outside of government restrictions. Of note, this is an area of rapid expansion in the literature.

For peer review only - http://bmjopen.bmj.com/site/about/guidelines.xhtml

DISCUSSION

The 23 articles included in this rapid scoping review revealed two main gaps in the literature: (1) telerehabilitation guidance (from research studies and guidance documents) is not specific to movement-related rehabilitation despite articles stating this explicitly as their aim, and (2) neither guidance nor training were given to providers in most research studies evaluating a telerehabilitation technology.

The Lack of Specific Guidance

The vast majority of the existing recommendations relate predominately to communication or governance elements of telerehabilitation, and lack specific guidance about issues pertinent to movement impairment. Several elements that are key to effectively implementing telerehabilitation for people with physical disability are missing. When assessing movement remotely, one would expect guidance on ensuring patient safety, triaging patients, and specific steps to decide whether a patient needs face-to-face care. More importantly, the identified guidance does not effectively address the limitations of undertaking physical assessments remotely; for example, if the patient is unable to move their body themselves if their movement is inadequate for assessment. Remote physical examinations are complex and need specific guidance on implementation; this is reflected in recent research that highlights physical examinations as one of the main challenges for remote assessments[38 39]. There was also minimal information on telerehabilitation related to COVID-19. This is unsurprising given the novelty of this infection. Overall, current telerehabilitation guidance for physical disabilities focuses on general tele-health guidance rather than being specific to movement impairments. Although issues such as data security, video etiquette, and organisation are important, they are not enough to assist providers in effective telerehabilitation implementation for people with movement impairments.

The Minimal Focus on Providers

Of the 11 included primary studies and 3 systematic reviews, only three[20 22 25] provided guidance to providers. Similarly, only four research studies[16 20 22 25] described the provision of training to providers. Neglecting providers' skills in using technology effectively (i.e. digital literacy) potentially undermines the usefulness of any telerehabilitation technology that was evaluated[40 41]. Furthermore, studies mainly report the clients' and not the providers' context of using telerehabilitation (e.g. whether the provider was from primary, secondary, or tertiary care). The generalisability of the telerehabilitation technology examined in the study is thus unclear, as certain technologies may only be effective in particular contexts[42]. Future studies should carefully consider providers' digital literacy and context to better understand the effectiveness of telerehabilitation technology. Digital literacy and context are not the only important factors for telerehabilitation implementation. Healthcare providers need appropriate organisational infrastructures (e.g. effective IT support) and a sufficient workflow integration to effectively implement technology[43]. They also reported that an easy-to-use interface is important, which aligns with the review's finding of needing a friendly client interface for technological improvements.

Digital Equity

Although digital equity was not part of this review's objectives, it is important to address. The included articles provide information based on the assumption that all clients (and providers) have both access and skills to engage in technology for remote assessments. Many of those currently digitally excluded are likely to become increasingly neglected from remote health services, thereby exacerbating existing health inequalities[44]. Future studies should acknowledge equity of access when examining telerehabilitation technologies. Future guidance documents should provide information for when clients do not have access to (or decline to use) telerehabilitation.

For peer review only - http://bmjopen.bmj.com/site/about/guidelines.xhtml

Comparisons with Other Studies

To our knowledge, this is the first scoping review of telerehabilitation guidance and training for assessing physical disabilities within daily clinical practice. Previous reviews mostly examine effectiveness within the context of research studies, and conclude that further provider and client training is needed to adequately implement telerehabilitation for physical assessments[45 46]. These studies conclude that telerehabiltiation technology has potential to deliver effective physical disability care, the current state of the art is insufficient for accurate remote movement assessment[47]. Our review confirms the need for further provider training, and additionally emphasises the lack of specific movement-related guidance.

Study Limitations

This scoping review has several limitations. Firstly, many telerehabilitation guidance documents for physical disabilities are likely unpublished. Although this review conducted an extensive search within grey literature sources, guidance documents and other relevant studies have likely been missed. Secondly, the search strategy was biased towards the English language. Despite no language restrictions during the search and the use of language translation software, relevant articles have likely been missed. This review also included a date restriction, where no articles prior to 2015 were included in the literature search in order to exclude outdated technological information. Yet, it should be noted that this may have also excluded some relevant aspects on guidance and training. Finally, this review was restricted by its rapid, cross-sectional approach. The authors acknowledge that some articles within this review may have been updated since the initial search and others have likely emerged since. This review may serve as a foundation for future research and development of guidance documents to provide information that addresses the gaps identified above.

Conclusion

There is a clear need for specific telerehabilitation guidance for physical disabilities, particularly for effective practice of remote physical assessments. Studies examining telerehabilitation technologies should deliver guidance and training to providers as well as document the context of providers if those technologies are to be effectively implemented. The catalyst that is the COVID-19 pandemic has forced the uptake of remote rehabilitation services, which are likely to persist beyond the pandemic. The development and maintenance of efficient telerehabilitation will not only require detailed guidance, but also active performance monitoring for on-going improvement of existing guidance, without which remote physical assessments may result in sub-optimal management.

Acknowledgments

We would like to sincerely thank Chris Johns from the University of Plymouth, who provided library support during the initial phase of this scoping review, and particularly assisted in developing the search strategy. Thanks to Sarah Chatfield who undertook the initial limited search of the literature.

Author Contributions

Krithika Anil planned and conducted the literature search, organised the review team, analysed and reported the results, and produced the initial manuscript. Jennifer Freeman conceptualised the study idea and supervised study processes. All authors (i.e. Krithika Anil, Jennifer Freeman, Sarah Buckingham, Sara Demain, Hilary Gunn, Ray Jones, Angela Logan, Jonathan Marsden, Diane Playford, Kim Sein, and Bridie Kent) reviewed the articles from the literature search, extracted data, contributed to data interpretation, and critically evaluated the manuscript. The corresponding author, Krithika Anil, attests that all listed authors meet authorship criteria and that no others meeting the criteria have been omitted.

For peer review only - http://bmjopen.bmj.com/site/about/guidelines.xhtml

Funding

This work was supported by UKRI-NIHR (MRC Section), Covid-19; Reference MR/V021060/1. The funder had no role in study design, data collection, data analysis, or writing of the report.

Competing Interests

None declared.

Data Sharing

No additional data available.

Dissemination to Participants and Related Patient and Public Communities

No participants were involved in this review. The research findings will be disseminated at relevant professional organisation and scientific conferences.

Ethics Statement

This study is a scoping literature review and did not involve participant data collection. Therefore, ethical approval was not required.

References

1. Peek N, Sujan M, Scott P. Digital health and care in pandemic times: impact of COVID-19. *BMJ Health and Care Informatics* 2020;27(1):e100166.
2. Brennan D, Tindall L, Theodoros D, et al. A blueprint for telerehabilitation guidelines. *Int J Telerehabil* 2010;2(2):31-34. doi: 10.5195/ijt.2010.6063
3. Charted Society of Physiotherapy. Rehabilitation and Covid-19 - CSP policy statement 2020 [Available from: https://www.csp.org.uk/professional-clinical/improvement-innovation/community-rehabilitation/rehab-covid-19-policy-statement.
4. British Society of Rehabilitation Medicine. Rehabilitation in the wake of Covid-19 - A phoenix from the ashes, 2020.
5. Wade DT. The future of rehabilitation in the United Kingdom National Health Service: Using the COVID-19 crisis to promote change, increasing efficiency and effectiveness: SAGE Publications Sage UK: London, England, 2020.
6. Wade DT. What attributes should a specialist in rehabilitation have? Seven suggested specialist Capabilities in Practice: SAGE Publications Sage UK: London, England, 2020.
7. Aromataris E, Munn Z. Chapter 1: JBI Systematic Reviews. In: Aromataris E, Munn Z, eds. JBI Manual for Evidence Synthesis: JBI 2020.
8. Tricco AC, Lillie E, Zarin W, et al. A scoping review on the conduct and reporting of scoping reviews. *BMC medical research methodology* 2016;16(1):15.
9. Peters MDJ, Marnie C, Tricco AC, et al. Updated methodological guidance for the conduct of scoping reviews. *JBI Evid Synth* 2020;18(10):2119-26. doi: 10.11124/jbies-20-00167 [published Online First: 2020/10/11]
10. Meyerson B. Top 10 emerging technologies of 2015 World Economic Forum2015 [Available from: https://www.weforum.org/agenda/2015/03/top-10-emerging-technologies-of-2015-2.
11. Wood L. Global Telepresence Robots Market (2020 to 2025) - Growth, Trends, and Forecast Business Wire2020 [Available from: https://www.businesswire.com/news/home/20201005005668/en/Global-Telepresence-Robots-Market-2020-to-2025---Growth-Trends-and-Forecast---ResearchAndMarkets.com.
12. Reuters T. EndNote X9. 2013
13. Ouzzani M, Hammady H, Fedorowicz Z, et al. Rayyan — a web and mobile app for systematic reviews. *Syst Rev* 2016(5):210. doi: 10.1186/s13643-016-0384-4
14. Kim H, Sefcik JS, Bradway C. Characteristics of Qualitative Descriptive Studies: A Systematic Review. *Res Nurs Health* 2017;40(1):23-42. doi: 10.1002/nur.21768 [published Online First: 2016/09/30]

For peer review only - http://bmjopen.bmj.com/site/about/guidelines.xhtml

15. Al Rajeh A, Steiner MC, Aldabayan Y, et al. Use, utility and methods of telehealth for patients with COPD in England and Wales: a healthcare provider survey. *BMJ Open Respir Res* 2019;6(1):e000345.
16. Middleton A, Simpson KN, Bettger JP, et al. COVID-19 Pandemic and Beyond: Considerations and Costs of Telehealth Exercise Programs for Older Adults With Functional Impairments Living at Home-Lessons Learned from a Pilot Case Study. *Phys Ther*
17. Anton D, Berges I, Bermúdez J, et al. A Telerehabilitation System for the Selection, Evaluation and Remote Management of Therapies. *Sensors (Basel)*;18(5)
18. Calvaresi D, Marinoni M, Dragoni AF, et al. Real-time multi-agent systems for telerehabilitation scenarios. *Artif Intell Med*;96:217-31.
19. Hasenohrl T, Windschnurer T, Dorotka R, et al. Prescription of individual therapeutic exercises via smartphone app for patients suffering from non-specific back pain A qualitative feasibility and quantitative pilot study. *Wien Klin Wochen*;132(5):115-23.
20. Levac D, Dumas HM, Meleis W. A Tablet-Based Interactive Movement Tool for Pediatric Rehabilitation: Development and Preliminary Usability Evaluation. *JMIR Rehabil Assist Technol*;5(2):e10307.
21. Paneroni M, Colombo F, Papalia A, et al. Is Telerehabilitation a Safe and Viable Option for Patients with COPD? A Feasibility Study. *Copd*;12(2):217-25.
22. Giesbrecht EM, Miller WC. Effect of an mHealth wheelchair skills training program for older adults: a feasibility randomized controlled trial. *Archives of Physical Medicine and Rehabilitation 2019 Nov;100(11):2159-2166* 2019
23. Grau-Pellicer M, Lalanza JF, Jovell-Fernandez E, et al. Impact of mHealth technology on adherence to healthy PA after stroke: a randomized study. *Topics in Stroke Rehabilitation 2020;27(5):354-368* 2020
24. Guerra de Oliveira Gondim IT, Cabral dos Santos Accioly Lins C, Jorge Asano NM, et al. Individualized guidance and telephone monitoring in a self-supervised home-based physiotherapeutic program in Parkinson. *Fisioterapia em Movimento [Physical Therapy in Movement] 2017 Jul-Sep;30(3):559-568* 2017
25. Venkataraman K, Morgan M, Amis KA, et al. Tele-Assessment of the Berg Balance Scale: Effects of Transmission Characteristics. *Arch Phys Med Rehabil*;98(4):659-64.e1.
26. Amatya B, Galea MP, Kesselring J, et al. Effectiveness of telerehabilitation interventions in persons with multiple sclerosis: A systematic review. *Mult Scler Relat Disord*;4(4):358-69.
27. Block VA, Pitsch E, Tahir P, et al. Remote Physical Activity Monitoring in Neurological Disease: A Systematic Review. *PLoS One* 2016;11(4):e0154335.
28. Mani S, Sharma S, Omar B, et al. Validity and reliability of Internet-based physiotherapy assessment for musculoskeletal disorders: a systematic review. *J Telemed Telecare*;23(3):379-91.
29. AHPScot. A guide to Musculoskeletal (MSK) Digital Health consultations during Coronavirus (COVID-19), 2020.
30. Health and Social Care Board. AHP Virtual Consultation Guidance, 2020.
31. Teleswallowing Ltd. Telehealth Service Delivery Guide, 2020.
32. Charted Society of Physiotherapy. Telephone guidance for musculoskeletal practice, 2020.
33. Charted Society of Physiotherapy. Digital tools to support service delivery, 2020.
34. Charted Society of Physiotherapy. COVID-19: guide for rapid implementation of remote consultations, 2020.
35. Royal College of Occupational Therapists. Virtual Assessments/Reviews: The Script. 2020
36. Royal College of Speech and Language Therapists. MAINTAINING SECURITY FOR TELEHEALTH SESSIONS, 2020.
37. East Lancashire Hospitals NHS Trust. Telepractice dysphagia assessmentsGuidelines for use with a patient in their own home/nursing home, 2020.
38. Seuren LM, Wherton J, Greenhalgh T, et al. Physical Examinations via Video for Patients With Heart Failure: Qualitative Study Using Conversation Analysis. *J Med Internet Res* 2020;22(2):e16694. doi: 10.2196/16694
39. Shaw SE, Seuren LM, Wherton J, et al. Video Consultations Between Patients and Clinicians in Diabetes, Cancer, and Heart Failure Services: Linguistic Ethnographic Study of Video-Mediated Interaction. *J Med Internet Res* 2020;22(5):e18378. doi: 10.2196/18378
40. Kuek A, Hakkennes S. Healthcare staff digital literacy levels and their attitudes towards information systems. *Health Informatics Journal* 2019;26(1):592-612. doi: 10.1177/1460458219839613

For peer review only - http://bmjopen.bmj.com/site/about/guidelines.xhtml

41. Poncette A-S, Glauert DL, Mosch L, et al. Undergraduate Medical Competencies in Digital Health and Curricular Module Development: Mixed Methods Study. *J Med Internet Res* 2020;22(10):e22161. doi: 10.2196/22161
42. Shaw J, Shaw S, Wherton J, et al. Studying Scale-Up and Spread as Social Practice: Theoretical Introduction and Empirical Case Study. *J Med Internet Res* 2017;19(7):e244. doi: 10.2196/jmir.7482
43. Palacholla RS, Fischer N, Coleman A, et al. Provider- and Patient-Related Barriers to and Facilitators of Digital Health Technology Adoption for Hypertension Management: Scoping Review. *JMIR cardio* 2019;3(1):e11951-e51. doi: 10.2196/11951
44. Majeed A, Maile EJ, Coronini-Cronberg S. Covid-19 is magnifying the digital divide: BMJ; 2020 [Available from: https://blogs.bmj.com/bmj/2020/09/01/covid-19-is-magnifying-the-digital-divide/ accessed Dec 2020.
45. Hosseiniravandi M, Kahlaee AH, Karim H, et al. Home-based telerehabilitation software systems for remote supervising: a systematic review. *International Journal of Technology Assessment in Health Care* 2020;36(2):113-25.
46. Peretti A, Amenta F, Tayebati SK, et al. Telerehabilitation: Review of the State-of-the-Art and Areas of Application. *JMIR rehabilitation and assistive technologies* 2017;4(2):e7.
47. Aderonmu JA. Emerging challenges in meeting physiotherapy needs during COVID-19 through telerehabilitation. *Bulletin of Faculty of Physical Therapy* 2020;25(1):16. doi: 10.1186/s43161-020-00018-4

For peer review only

For peer review only - http://bmjopen.bmj.com/site/about/guidelines.xhtml

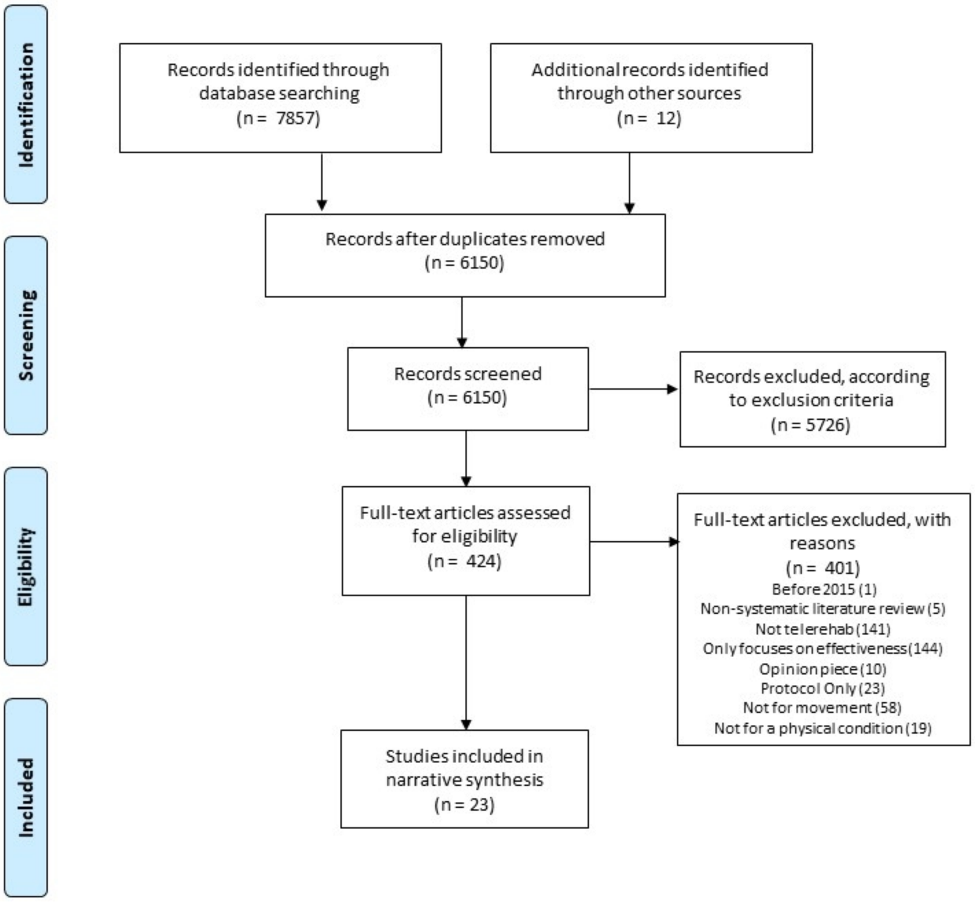

PRISMA flow diagram that charts the study identification process

192x173mm (96 x 96 DPI)

Search Strategy Terms

The below search strategy uses a logic grid and is based on the PCC framework; additional columns were added to aid in search sensitivity. As advised by an expert librarian, "concept 3" column was only used to narrow searches that produced an overwhelming quantity of results.

Population 1	*(Population 2)*	*Concept 1*	*Concept 2*	*Concept 3*	*Context*
Physical condition	Covid*	Tele*	Training	Movement	Healthcare
Physical disability	Coronavirus	Virtual	Guidance	Mobility	Health care
Physical impairment	2019-nCoV	Online	Assessment	Function	Clinic
	SARS-CoV-2	Video	Tool*	Gait	Social care
	Severe acute respiratory syndrome coronavirus 2	Digital	Protocol	Walking	Hospital
		Multimedia	Battery	Balance	Practice
		Mobile		Motor	
		Phone		Strength	
		mHealth		Power	
		eHealth		Tone	
				Range	
				Contracture	
				Fatigue	
				Stamina	
				Dexterity	
				Physical capacity	

Search term strategy:

1. Each column was searched individually, e.g. physical condition OR physical disability OR physical impairment
2. All individual searches based on each column were combined, e.g. (physical condition OR physical disability OR physical impairment)AND (Tele* OR Virtual OR Online OR Video OR Digital OR Multimedia OR Mobile OR Phone OR mHealth OR eHealth)
3. The search strategy proceeded as follows (after searching for each column individually):
    a. Column 1 AND column 3 AND column 4 AND column 5
    b. If there were too many hits based on a., column 6 wasto be added

   c. Column 2 was only to be used as an add-on after searches to keep a separate record of covid-related literature for the narrative synthesis
4. Unpublished literature searches used a combination of column 1 and 3 in the first instance, where searches were tailored according to individual database searching tools.
5. Date limiter: 2015 onwards.

For peer review only

Record of Online Searches

Database: PubMed
Date: 20/07/20

ID	Search Term(s)	Hits
1	(physical condition) OR (physical disability) OR (physical impairment)	641,602
2	(tele*) OR (virtual) OR (online) OR (video) OR (digital) OR (multimedia) OR (mobile) OR (phone) OR (mhealth) OR (ehealth)	862,859
3	(training) OR (guidance) OR (assessment) OR (tool*) OR (protocol) OR (battery)	5,791,855
4	(movement) OR (mobility) OR (function) OR (gait) OR (walking) OR (balance) OR (motor) OR (strength) OR (power) OR (tone) OR (range) OR (contracture) OR (fatigue) OR (stamina) OR (dexterity) OR (physical capacity)	15,600,817
5	(ID 1) AND (ID 2) AND (ID 3) AND (ID 4) with date limiter: 2015 onwards	5,322

Database: CINHAL
Date: 03/08/20

ID	Search Term(s)	Hits
1	(physical condition) OR (physical disability) OR (physical impairment)	52,385
2	(tele*) OR (virtual) OR (online) OR (video) OR (digital) OR (multimedia) OR (mobile) OR (phone) OR (mhealth) OR (ehealth)	267,357
3	(training) OR (guidance) OR (assessment) OR (tool*) OR (protocol) OR (battery)	1,100,793
4	(movement) OR (mobility) OR (function) OR (gait) OR (walking) OR (balance) OR (motor) OR (strength) OR (power) OR (tone) OR (range) OR (contracture) OR (fatigue) OR (stamina) OR (dexterity) OR (physical capacity)	902,661
5	(ID 1) AND (ID 2) AND (ID 3) AND (ID 4) with date limiter: 2015 onwards	197

Database: PsychInfo
Date: 03/08/20

ID	Search Term(s)	Hits
1	(physical condition) OR (physical disability) OR (physical impairment)	148,089
2	(tele*) OR (virtual) OR (online) OR (video) OR (digital) OR (multimedia) OR (mobile) OR (phone) OR (mhealth) OR (ehealth)	240,422

For peer review only

3	(training) OR (guidance) OR (assessment) OR (tool*) OR (protocol) OR (battery)	1,057,337
4	(movement) OR (mobility) OR (function) OR (gait) OR (walking) OR (balance) OR (motor) OR (strength) OR (power) OR (tone) OR (range) OR (contracture) OR (fatigue) OR (stamina) OR (dexterity) OR (physical capacity)	1,159,877
5	(ID 1) AND (ID 2) AND (ID 3) AND (ID 4) with date limiter: 2015 onwards	557

Database: Cochrane Library (Reviews Only)
Date: 03/08/20

ID	Search Term(s)	Hits
1	(physical condition OR physical disability OR physical impairment) AND<br>(tele* OR virtual OR online OR video OR digital OR multimedia OR mobile OR phone OR mhealth OR ehealth) AND<br>(training OR guidance OR assessment OR tool* OR protocol OR battery) AND<br>(movement OR mobility OR function OR gait OR walking OR balance OR motor OR strength OR power OR tone OR range OR contracture OR fatigue OR stamina OR dexterity OR physical capacity)<br>with date limiter: 2015 onwards	17

Database: EMBASE
Date: 05/08/20

ID	Search Term(s)	Hits
1	physical condition OR physical disability OR physical impairment	31,388
2	tele* OR virtual OR online OR video OR digital OR multimedia OR mobile OR phone OR mhealth OR ehealth	973,017
3	training OR guidance OR assessment OR tool* OR protocol OR battery	457,1408
4	movement OR mobility OR function OR gait OR walking OR balance OR motor OR strength OR power OR tone OR range OR contracture OR fatigue OR stamina OR dexterity OR physical capacity	700,1843
5	(ID 1) AND (ID 2) AND (ID 3) AND (ID 4) with date limiter: 2015 onwards	157

Database: Web of Science
Date: 05/08/20

For peer review only

ID	Search Term(s)	Hits
1	TS=(physical condition OR physical disability OR physical impairment)	277,584
2	TS=(tele* OR virtual OR online OR video OR digital OR multimedia OR mobile OR phone OR mhealth OR ehealth)	2,360,472
3	TS=(training OR guidance OR assessment OR tool* OR protocol OR battery)	4,974,907
4	TS=(movement OR mobility OR function OR gait OR walking OR balance OR motor OR strength OR power OR tone OR range OR contracture OR fatigue OR stamina OR dexterity OR physical capacity)	12,757,447
5	(ID 1) AND (ID 2) AND (ID 3) AND (ID 4) with date limiter: 2015 onwards	1,720

Database: PEDro
Date: 06/08/20

ID	Search Term(s)	Hits
1	Tele* AND training with date limiter: 2015 onwards	79
2	Tele* AND guidance with date limiter: 2015 onwards	12
3	mHealth AND training with date limiter: 2015 onwards	8
4	mHealth AND guidance with date limiter: 2015 onwards	1
5	eHealth AND training with date limiter: 2015 onwards	1
6	eHealth AND guidance with date limiter: 2015 onwards	0

Database: UK Health Forum
Date: 11/08/20

ID	Search Term(s)	Hits
1	Tele	0
2	Telehealth	0
3	Telemedicine	0
4	Telerehabilitation	0
5	Tele rehabilitation	0
6	mhealth	0
7	ehealth	1

For peer review only

8	Virtual	0
9	Online	4
10	Video	0
11	Digital	0
12	Multimedia	0
13	Mobile	0
14	Phone	0

Database: National Archives (UK)
Date: 06/08/20

ID	Search Term(s)	Hits
1	Tele* with date limiter: 2015 onwards	37
2	mhealth with date limiter: 2015 onwards	0
3	ehealth with date limiter: 2015 onwards	1

Within each search of this database, hits represent websites that can be searched using National Archives. Each database was searched using "training" or "guidance"; no hits were returned in any searches.

Database: WHO
Date: 11/08/20

ID	Search Term(s)	Hits
1	Telehealth AND training with date limiter: 2015 onwards	118
2	Telemedicine AND training with date limiter: 2015 onwards	353
3	Telemedicine AND guidance with date limiter: 2015 onwards	294
4	Telehealth AND guidance with date limiter: 2015 onwards	111
5	mHealth AND training with date limiter: 2015 onwards	384
6	mHealth AND guidance with date limiter: 2015 onwards	344

Database: NHS England
Date: 11/08/20

ID	Search Term(s)	Hits
1	Telehealth with date limiter: 2015 onwards	19

For peer review only

2	Telemedicine with date limiter: 2015 onwards	19
3	ehealth with date limiter: 2015 onwards	18
4	mhealth with date limiter: 2015 onwards	22

Database: Charted Society of Physiotherapy
Date: 19/08/20

ID	Search Term(s)	Hits
1	Tele*	12
2	mHealth	4
3	eHealth	23
4	Tele* AND training	3538
5	Tele* AND guidance	2849

For peer review only

Procedural Guidance for Care Delivery – Derived from Recommendations Analysis

While the information below has been derived from articles that explored movement impairment, none of the recommended actions were specific for movement impairment interventions.

Procedural Guidance	Description
Pre-Delivery<br>▪ Environment Considerations<br>▪ Session Planning<br>▪ Technology Considerations<br>▪ Preparations with Client	In preparation for a telerehabilitation session, including actions such as planning the session environment, preparing the required technology, contacting the client, etc.
During Delivery<br>▪ Start<br>▪ Throughout<br>▪ End	During a telerehabilitation session, including actions such as introducing the agenda for that session, noting technical and safety concerns, summarising the session, etc.
Post-Delivery	After a telerehabilitation session, including actions such as documenting key messages of the session, deciding whether the patient needs further/additional support, and taking breaks when needed.

Pre-Delivery

Thirteen articles[1-13] suggested actions to take before engaging in a telerehabilitation session. These actions can be divided into Preparations with the Client, Environment Considerations, Session Planning, and Technology Considerations.

- Preparations with the Client: Contact the client to assess whether they are clinically appropriate to engage in telerehabilitation (e.g. whether face-to-face or urgent care is needed instead). If telerehabilitation is deemed appropriate, ensure the client has all necessary equipment (including suitable data/WIFI accessibility) and, if required, test any equipment with the client. Provide any information the client needs to attend the telerehabilitation session (e.g. link to logon to session, passwords, times, private and well-lit room, or other resources.). Ensure the client's phone number is available in case of technological issues during the session. Consider the client's context (e.g. hard of hearing or anxious about using technology) and address the context as appropriate if possible.
- Environment Considerations: Ensure the location from which one provides the telerehabilitation is quiet, private, not cluttered and will not be disturbed. Curtains should be drawn to reduce any on-screen glare from the sun. Clients should ensure the location is well-lit without relying on the sun. Test any technology (especially video and audio) to check they are working properly. If conducting a group session, ensure the size of the class is appropriate for the activities planned.
- Session Planning: Evaluate the client's functional status prior to the session and develop a clear agenda. Consider whether a client would benefit from a group or one-to-one session. If planning a group session, ensure there is enough time to address everyone's technological issues (e.g. connectivity issues, audio issues, etc.). Consider how many other staff members you may need (e.g. an extra member for support during a group session).

For peer review only

- Technology Considerations: Ensure any technology used is appropriate for the agenda of that session. Ensure there are back-up devices that can be used (e.g. telephone) if the main technology fails. Professional and patient clients should only open the software needed for the session and all other software should be closed. Ensure internet-using technology is wired to or close to the WIFI router, and that all required equipment are close-by. Position any cameras so that the whole face is within direct view. Ensure that all providers know how to use the required technology (e.g. knowing how to record or take screen shots) prior to the session.

During Delivery

Seven articles[1, 5, 7-11] suggested actions that should occur during the telerehabilitation session, which can be divided into the Start of the session, During the session, and the End of the session.

- Start: Welcome the client and ensure audio and video are working correctly. Confirm the identity of everyone attending the session. If a trusted other (e.g. carer) is also attending, check that the client is comfortable with that trusted other. If possible, lock the meeting to prevent others from entering. During a group session, ensure everyone in the group introduces themselves (including all staff). Summarise the session's agenda and introduce the roles of any trusted other or staff (e.g. assisting with note taking). Ensure the client(s) know what to do if the session is cut. Ask the client if they have any questions or concerns before engaging in the telerehabilitation session. Ensure the client is still clinically appropriate to engage in telerehabilitation (i.e. they do not need face-to-face or urgent care). If taking screen shots or recording, ensure the client knows and you have their consent. Finally, ask the client to show their environment to ensure any rehabilitation activities can be conducted safely and accurately.
- During: Document activities as per your professional governing body requirements, yet also include any technical issues and safety concerns from the client. Be patient during technical glitches (e.g. blurred video or audio cutting out). Understand that communication is harder for the client and ensure communication is intentional (e.g. verbal cues to let the client know you are listening). Ensure that you encourage the client to engage (e.g. by asking questions). The professional should inform the client when and why they might be pre-occupied (e.g. taking notes or looking for a resource).
- End: Carefully and clearly summarise key messages from the session. Let the client know if another session is required and when that will take place. Similarly, let the client know the required details if they need to be referred to another service. Confirm and record that the client is comfortable in engaging in further telerehabilitation sessions. Ensure the client knows self-care activities and their regime. Ask the client if anything needs to be clarified. Provide the client contact details if they need to contact the provider again. When ending the call, let the client know explicitly and then end the call.

Post Delivery

Three articles[5, 10, 11] suggested actions that should occur after a telerehabilitation session. Re-evaluate the client's functional ability. Document key messages of the session as required. Consider if a follow-up call or other structural support is needed for the client. Ensure to take breaks between telerehabilitation sessions to reduce any fatigue and look after vocals.

For peer review only

References

1. AHPScot, *A guide to Musculoskeletal (MSK) Digital Health consultations during Coronavirus (COVID-19)*. 2020.
2. Anton, D., et al., *A Telerehabilitation System for the Selection, Evaluation and Remote Management of Therapies.* Sensors (Basel). **18**(5).
3. Block, V.A., et al., *Remote Physical Activity Monitoring in Neurological Disease: A Systematic Review.* PLoS One, 2016. **11**(4): p. e0154335.
4. Calvaresi, D., et al., *Real-time multi-agent systems for telerehabilitation scenarios.* Artif Intell Med. **96**: p. 217-231.
5. Health and Social Care Board, *AHP Virtual Consultation Guidance*. 2020.
6. Paneroni, M., et al., *Is Telerehabilitation a Safe and Viable Option for Patients with COPD? A Feasibility Study.* Copd. **12**(2): p. 217-25.
7. Charted Society of Physiotherapy, *COVID-19: guide for rapid implementation of remote consultations*. 2020.
8. Royal College of Occupational Therapists, *Virtual Assessments/Reviews: The Script.* 2020.
9. Royal College of Speech and Language Therapists, *MAINTAINING SECURITY FOR TELEHEALTH SESSIONS*. 2020.
10. East Lancashire Hospitals NHS Trust, *Telepractice dysphagia assessmentsGuidelines for use with a patient in their own home/nursing home*. 2020.
11. Middleton, A., et al., *COVID-19 Pandemic and Beyond: Considerations and Costs of Telehealth Exercise Programs for Older Adults With Functional Impairments Living at Home-Lessons Learned from a Pilot Case Study.* Phys Ther.
12. Charted Society of Physiotherapy, *Telephone guidance for musculoskeletal practice*. 2020.
13. Charted Society of Physiotherapy, *Digital tools to support service delivery*. 2020.

For peer review only

Preferred Reporting Items for Systematic reviews and Meta-Analyses extension for Scoping Reviews (PRISMA-ScR) Checklist

SECTION	ITEM	PRISMA-ScR CHECKLIST ITEM	REPORTED ON PAGE #
**TITLE**			
Title	1	Identify the report as a scoping review.	
**ABSTRACT**			
Structured summary	2	Provide a structured summary that includes (as applicable): background, objectives, eligibility criteria, sources of evidence, charting methods, results, and conclusions that relate to the review questions and objectives.	
**INTRODUCTION**			
Rationale	3	Describe the rationale for the review in the context of what is already known. Explain why the review questions/objectives lend themselves to a scoping review approach.	
Objectives	4	Provide an explicit statement of the questions and objectives being addressed with reference to their key elements (e.g., population or participants, concepts, and context) or other relevant key elements used to conceptualize the review questions and/or objectives.	
**METHODS**			
Protocol and registration	5	Indicate whether a review protocol exists; state if and where it can be accessed (e.g., a Web address); and if available, provide registration information, including the registration number.	
Eligibility criteria	6	Specify characteristics of the sources of evidence used as eligibility criteria (e.g., years considered, language, and publication status), and provide a rationale.	
Information sources*	7	Describe all information sources in the search (e.g., databases with dates of coverage and contact with authors to identify additional sources), as well as the date the most recent search was executed.	
Search	8	Present the full electronic search strategy for at least 1 database, including any limits used, such that it could be repeated.	
Selection of sources of evidence†	9	State the process for selecting sources of evidence (i.e., screening and eligibility) included in the scoping review.	
Data charting process‡	10	Describe the methods of charting data from the included sources of evidence (e.g., calibrated forms or forms that have been tested by the team before their use, and whether data charting was done independently or in duplicate) and any processes for obtaining and confirming data from investigators.	
Data items	11	List and define all variables for which data were sought and any assumptions and simplifications made.	
Critical appraisal of individual sources of evidence§	12	If done, provide a rationale for conducting a critical appraisal of included sources of evidence; describe the methods used and how this information was used in any data synthesis (if appropriate).	
Synthesis of results	13	Describe the methods of handling and summarizing the data that were charted.	

For peer review only - http://bmjopen.bmj.com/site/about/guidelines.xhtml

SECTION	ITEM	PRISMA-ScR CHECKLIST ITEM	REPORTED ON PAGE #
**RESULTS**			
Selection of sources of evidence	14	Give numbers of sources of evidence screened, assessed for eligibility, and included in the review, with reasons for exclusions at each stage, ideally using a flow diagram.	
Characteristics of sources of evidence	15	For each source of evidence, present characteristics for which data were charted and provide the citations.	
Critical appraisal within sources of evidence	16	If done, present data on critical appraisal of included sources of evidence (see item 12).	
Results of individual sources of evidence	17	For each included source of evidence, present the relevant data that were charted that relate to the review questions and objectives.	
Synthesis of results	18	Summarize and/or present the charting results as they relate to the review questions and objectives.	
**DISCUSSION**			
Summary of evidence	19	Summarize the main results (including an overview of concepts, themes, and types of evidence available), link to the review questions and objectives, and consider the relevance to key groups.	
Limitations	20	Discuss the limitations of the scoping review process.	
Conclusions	21	Provide a general interpretation of the results with respect to the review questions and objectives, as well as potential implications and/or next steps.	
**FUNDING**			
Funding	22	Describe sources of funding for the included sources of evidence, as well as sources of funding for the scoping review. Describe the role of the funders of the scoping review.	

JBI = Joanna Briggs Institute; PRISMA-ScR = Preferred Reporting Items for Systematic reviews and Meta-Analyses extension for Scoping Reviews.

* Where *sources of evidence* (see second footnote) are compiled from, such as bibliographic databases, social media platforms, and Web sites.

† A more inclusive/heterogeneous term used to account for the different types of evidence or data sources (e.g., quantitative and/or qualitative research, expert opinion, and policy documents) that may be eligible in a scoping review as opposed to only studies. This is not to be confused with *information sources* (see first footnote).

‡ The frameworks by Arksey and O'Malley (6) and Levac and colleagues (7) and the JBI guidance (4, 5) refer to the process of data extraction in a scoping review as data charting.

§ The process of systematically examining research evidence to assess its validity, results, and relevance before using it to inform a decision. This term is used for items 12 and 19 instead of "risk of bias" (which is more applicable to systematic reviews of interventions) to include and acknowledge the various sources of evidence that may be used in a scoping review (e.g., quantitative and/or qualitative research, expert opinion, and policy document).

From: Tricco AC, Lillie E, Zarin W, O'Brien KK, Colquhoun H, Levac D, et al. PRISMA Extension for Scoping Reviews (PRISMAScR): Checklist and Explanation. Ann Intern Med. 2018;169:467–473. doi: 10.7326/M18-0850.

For peer review only - http://bmjopen.bmj.com/site/about/guidelines.xhtml
